# SULBA: A Task-Agnostic Data Augmentation Framework for Deep Learning in Medical Image Analysis

**DOI:** 10.3390/diagnostics16101546

**Published:** 2026-05-19

**Authors:** Ayomide Adeyemi Abe, Mpumelelo Nyathi

**Affiliations:** 1 Department of Medical Physics, School of Medicine, Sefako Makgatho Health Sciences University, Pretoria 0028, South Africa; 2 AureXida Inc., Toronto, ON M4M 1Y3, Canada

**Keywords:** data augmentation, medical imaging, medical diagnosis, deep learning, artificial intelligence, medical image analysis

## Abstract

**Background/Objectives**: Data augmentation is a foundational component of modern deep learning for enhancing robustness and generalization. However, medical imaging lacks a universally reliable augmentation strategy, forcing researchers into an inefficient “augmentation lottery” that hinders experimental progress and reproducibility. To address this challenge, we introduce Stepwise Upper and Lower Boundaries Augmentation (SULBA), a simple, parameter-free framework designed to eliminate per-task augmentation tuning. **Methods**: SULBA generates training variations through stepwise cyclic shifts applied along data dimensions, making it inherently applicable to 2D, 3D, and higher-dimensional medical imaging data. To evaluate the efficacy of SULBA as a default DA strategy, we performed benchmarking across 27 publicly available datasets spanning classification and segmentation tasks and 10 convolutional and transformer-based architectures using standard deep learning performance metrics. **Results**: The results demonstrate that SULBA achieves the highest overall performance and consistently outperforms 16 widely used standard augmentation techniques while delivering robust and reliable improvements without task- or parameter-specific tuning **Conclusions**: SULBA establishes a principled universal default for data augmentation in medical imaging, with the potential to accelerate the development of generalizable and reproducible medical AI systems.

## 1. Introduction

Deep learning has become a cornerstone of modern medical image analysis, enabling substantial advances in tasks such as disease classification, lesion detection, and anatomical segmentation [1,2,3]. Architectures such as convolutional neural networks (CNNs) have demonstrated strong performance across a wide range of imaging modalities by learning hierarchical representations directly from pixel or voxel data [4]. More recently, transformer-based and hybrid architectures have further extended modeling capacity through enhanced long-range contextual reasoning and hierarchical attention mechanisms [5,6]. These advances hold promise for improved diagnostic accuracy, reduced clinician workload, and enhanced patient outcomes. However, achieving robust and generalizable performance with such data-hungry models fundamentally depends on access to large, diverse, and well-annotated datasets [7].

The availability of robust medical imaging datasets is fundamentally constrained by several factors. Expert annotation is expensive and time-consuming, class imbalance is pervasive, and data sharing is restricted by privacy, ethical, and regulatory requirements [8,9]. As a result, many medical imaging studies operate in data-limited regimes, where overfitting and poor generalization remain persistent challenges [10,11]. These limitations have made data augmentation (DA) an indispensable component of medical deep learning pipelines, providing a mechanism to expand training datasets through diverse mechanisms [12].

Existing DA approaches broadly fall into two paradigms: data generation and data transformation. Generative methods such as variational autoencoders, generative adversarial networks, and diffusion models synthesize new samples that approximate the statistical distribution of the original training data [13,14,15]. Transformation-based approaches instead apply predefined operations such as rotation, flipping, cropping, intensity perturbation, or elastic deformation to existing images [12,16]. While both paradigms have shown benefits, they present critical challenges when applied to medical imaging.

A central requirement of medical data augmentation is the preservation of diagnostic validity [12,17]. Generative approaches may exhibit hidden failure modes, leading to the generation of anatomically implausible structures or subtle artifacts that are difficult to detect without expert review, thereby compromising model reliability and clinical trust [18,19]. Transformation-based techniques can similarly undermine diagnostic integrity [17]. For instance, mixing-based augmentations may introduce biologically implausible tissue combinations that obscure true anatomy and degrade learning, while occlusion-based and aggressive cropping can eliminate clinically salient regions [20]. Additionally, commonly used geometric transformations may disrupt anatomical context or alter spatial relationships that are diagnostically meaningful, particularly when applied without domain-specific considerations [12,21].

These data constraints are compounded by domain-specific challenges that distinguish medical from natural image analysis [18]. Effective augmentation must preserve anatomical and pathological fidelity, avoiding transformations that generate biologically implausible tissue structures or relationships. Furthermore, diagnostic features in medical images often rely on subtle variations in low-texture contrast and shape, rather than the high-frequency textures and colors dominant in natural images [12]. This is coupled with vast inter-patient anatomical variability and heterogeneity in acquisition protocols across clinical sites [21]. Consequently, augmentation strategies developed for natural images may be inappropriate or even detrimental when applied directly to medical data [20].

These limitations create a persistent tension between inducing sufficient variability aimed at improving model generalization and preserving the anatomical and pathological fidelity required for clinical relevance, exposing a deeper systemic inefficiency in current practice [13]. In the absence of a universally reliable augmentation strategy, researchers are often compelled to empirically evaluate numerous augmentation techniques and hyperparameter configurations for each new task, imaging modality, and network architecture, which impedes reproducibility and constitutes a significant experimental bottleneck [22]. Although many augmentation methods are standardized and readily accessible through widely adopted deep learning frameworks such as PyTorch [23], TorchIO [24], and TensorFlow [25], the lack of a principled, universally applicable strategy continues to necessitate empirical, task-specific and architecture-dependent selection [26]. This phenomenon, often described as an “augmentation lottery”, represents a significant bottleneck in medical AI research, slowing progress, increasing experimental uncertainty, and hindering reproducibility [22,27].

To address this challenge, we introduce Stepwise Upper and Lower Boundaries Augmentation (SULBA), a simple, parameter-free, perfectly reversible, and dimension-agnostic data augmentation framework. SULBA generates novel training samples via stepwise cyclic shifts applied along data dimensions (e.g., height, width, or depth), making it inherently invariant to data dimensionality and feature composition. It can be applied seamlessly to 2D, 3D, and higher-dimensional data, including single- and multi-channel images without architectural modifications or per-task hyperparameter tuning. While each shift operation is deterministic, diversity arises from stochastic selection of shift offsets during training. This structured reordering aligns with principles explored in permutation-invariant and equivariant learning, where robustness arises from controlled input reordering rather than content corruption [28,29,30].

**Figure 1:**
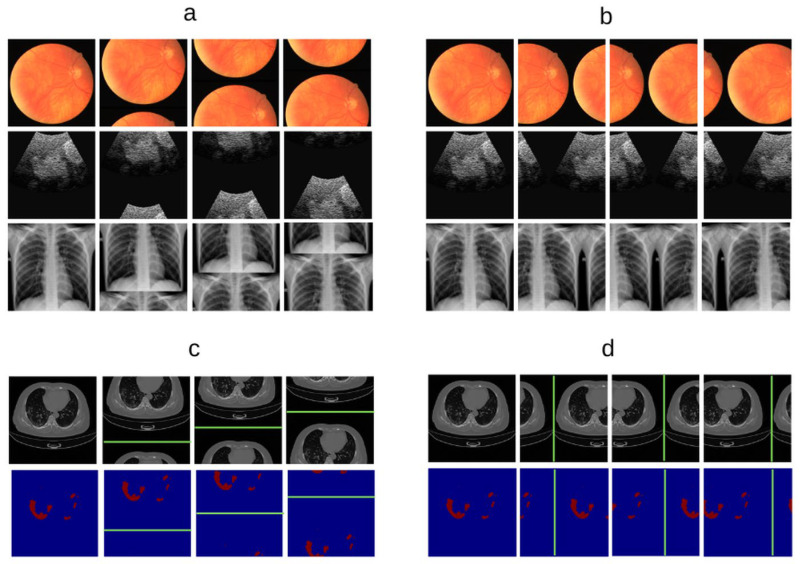
Stepwise Upper and Lower Boundaries Augmentation (SULBA). (**a**) Illustration of the SULBA operation applied along the height dimension of a 2D image. (**b**) Illustration of the operation applied along the width dimension. (**c**) Paired example of a 2D chest CT scan (**top**) and its corresponding tumor segmentation mask (**bottom**) after SULBA is applied along the height dimension. (**d**) Paired example showing the same transformation applied along the width dimension. For all panels, the first column shows the original data, green lines depict the complementary partial views created by the cyclic shift, and columns 2–4 show three distinct augmented outputs generated using different random step sizes.

Unlike conventional augmentation strategies that interpolate, corrupt, or replace image content, SULBA preserves all original information by systematically repositioning contiguous regions through cyclic shifts. This transformation produces complementary partial views within a single image, preserving pixel intensities as well as the integrity of corresponding local tissue structure and pathology while introducing coherent global variation (Figure 1). As a result, SULBA exposes models to anatomically plausible reconfiguration in which salient features appear in altered spatial or feature contexts, reducing reliance on absolute position and encouraging robust, position-invariant representation learning. These properties are particularly advantageous in medical imaging, where preserving diagnostic information and maintaining pathological fidelity are essential [19,31].

While the concept of a cyclic shift is mathematically straightforward, SULBA distinguishes itself from related ideas in several key aspects. SULBA does not follow a general random permutation or patch reordering, which can disrupt clinically vital local anatomical structures [17]. Instead, SULBA’s stepwise cyclic shift preserves *contiguous segments* of the original data, maintaining the integrity of local features while systematically varying their global context. Furthermore, unlike equivariant networks, which build transformation invariance into the model architecture, SULBA is an input-space strategy that encourages robust, position-invariant feature learning in any standard model. This design is specifically motivated by the need for anatomically plausible variations in medical data, ensuring all original diagnostic information is retained in a perfectly reversible manner.

This work makes two key contributions. First, we propose SULBA as a universal, parameter-free augmentation framework that is reversible, dimension-agnostic, and directly applicable across imaging modalities, data dimensions, and task types. Second, to our knowledge, we present the most comprehensive empirical evaluation of data augmentation methods in medical imaging to date, benchmarking 27 publicly available datasets across 10 convolutional and transformer-based architectures for both classification and segmentation tasks in 2D and 3D domains. Across this diverse experimental landscape, SULBA consistently achieves superior performance, outperforming 16 widely used augmentation strategies while eliminating the need for task-specific augmentation tuning, thereby enabling more robust, reproducible, and scalable medical AI development.

## 2. Materials and Methods

### 2.1. SULBA Framework

Stepwise Upper and Lower Boundaries Augmentation (SULBA) is a deterministic, dimension-agnostic data augmentation framework. The framework comprises a stochastic application protocol that is specifically optimized for medical imaging with inherent synchronization for segmentation tasks. For any input image tensor $X\;\in R^{C{\times D}_{1}\times D_{2}\times \ldots \times D_{N}}$ defined over feature (C) and spatial dimensions (D), SULBA generates a novel sample by applying a cyclic shift along a randomly selected data dimension (k). A SULBA transformation along dimension k, (k ∈ {0, …, N}) is defined as a cyclic shift operation governed by the modulo function described by

(1) $$SULBA(X)=X[:,\ldots ,(d_{k}+s_{k})\;mod\;D_{k,}\ldots ]$$

where mod denotes the modulo operation,

- $d_{k\;}$ ∈ {0,…,${\;D}_{k\;}-\;1$} is a specific location along dimension k.
- $s_{k}\in \{1,\ldots ,D_{k}-1$} is a randomly sampled step size.

As shown in Equation (1), the cyclic shift operation is deterministic and perfectly reversible. During training, diversity is introduced by stochastically sampling both the dimension (k) to be shifted and the step size $\left(s_{k}\right)$ at each application. This design preserves all original voxel intensities and local structures while systematically reconfiguring the global spatial or feature context of the input. For segmentation tasks, identical shift parameters $\left({k,\;s}_{k}\right)$ are applied synchronously to both the input image and its corresponding label mask (Figure 1c), ensuring pixel- or voxel-perfect alignment. The complete SULBA procedure is summarized in Algorithm 1.
**Algorithm 1** Stepwise Upper and Lower Boundaries Augmentation (SULBA)Input:    Data tensor $X\;\in R^{C{\times D}_{1}\times D_{2}\times \ldots \times D_{N}}$; stride $s_{k\;}\;\in \{1,\ldots ,.\;D_{k\;}-\;1$} **Output:**    Transformed tensor $X^{\prime }$
Procedure: 1. **Initialize:** For each dimension k, determine the set of possible cyclic shift offsets $s_{k\;}\;\in \{1,2,\ldots ,\;D_{k\;}-\;1$} **2. For each selected dimension** k:              1.Randomly select a shift $s_{k\;}$ from the possible offsets.              2.For each index $D_{k\;}\;\in \{0,\;D_{k\;}-\;1$}, compute the shifted index:              3.$d_{k}^{\prime }\;=\;(d_{k\;}+s_{k\;})\;mod\;D_{k\;}$              4.Rearrange X along dimension k according to the shifted indices $d_{k}^{\prime }$.
**3. Return:** The transformed tensor $X^{\prime }$.

### 2.2. Scaling of Generated Samples

Each term $D_{k\;}-\;1$ corresponds to the set of all valid step sizes along k, and shifts are applied along one dimension at a time. The resulting sample generation is additive across dimensions and automatically adapts to the complexity of the input data. Consequently, larger or higher-dimensional images yield a greater diversity of training samples without requiring manual parameter tuning, and the number of novel samples generated by SULBA scales with both image resolution and dimensionality. For an input tensor X, $X\;\in R^{C{\times D}_{1}\times D_{2}\times \ldots \times D_{N}}$; the total number of possible novel configurations is given by

(2) $$Total\;samples=\sum_{k-0}^{N}\left(D_{k}-1\right)$$

### 2.3. SULBA Perfect Reversibility

SULBA transformation guarantees perfect reversibility due to the invertible nature of cyclic shifts [28,29,30]. Let X denote an input tensor, and let $X^{\prime }$ represent a cyclic shift of X along dimension k by a step size s. The original input can be exactly recovered by applying a complementary shift of $D_{k\;}-\;s$ as shown in Equations (3) and (4). Additionally, SULBA does not perform interpolation, cropping, or any pixel-level modification. This deterministic and reversible property ensures the preservation of anatomical and pathological content within each transformed view, facilitating reliable and reproducible model training. Formally, if

(3) X’=CyclicShift(X,k,s)

Then the inverse operation is given by

(4) $$X=CyclicShift(X’,k,D_{k}-s)$$

where $D_{k\;}$ denotes the size of dimension k.

### 2.4. Datasets and Preprocessing

Benchmarking was conducted across 27 publicly available medical imaging datasets spanning four task categories: 2D classification, 3D classification, 2D segmentation, and 3D segmentation.

For 2D classification, the BloodMNIST, BreastMNIST, DermaMNIST, OctMNIST, OrganAMNIST, OrganCMNIST, OrganSMNIST, PathMNIST, PneumoniaMNIST, and TissueMNIST were selected from the MedMNIST v2 [32] suite. Six volumetric MedMNIST v2 datasets—AdrenalMNIST3D, FractureMNIST3D, NoduleMNIST3D, OrganMNIST3D, SynapseMNIST3D, and VesselMNIST3D—were used for 3D classification.

For 2D segmentation, seven datasets were analyzed from the MedSegBench datasets [33]: AbdomenUSMSBench, Bkai-Igh-MSBench, CystoFluidMSBench, DeepbacsMSBench, FHPsAOPMSBench, MosMedPlusMSBench, and Promise12MSBench. For 3D segmentation, experiments were conducted on IXITiny [24] and the Medical Segmentation Decathlon (MSD) datasets [34] for the Heart and Hippocampus tasks. A complete dataset description is provided in Supplementary Table S30.

For 2D data, preprocessing included normalization using ImageNet [35] statistics and conversion of grayscale images to three channels to enable the use of pretrained weights. For 3D data, images were standardized to canonical orientation, normalized using Z-score normalization, and rescaled to an intensity range of [−1, 1]. When native image sizes varied, inputs were resized to match the architectural requirements of each model.

For cross-dataset generalization, models trained on PneumoniaMNIST were evaluated on a publicly available chest X-ray pneumonia dataset [36] using identical preprocessing pipelines. A summary of dataset characteristics, including resolution, sample count, class distribution, modality and preprocessing details, is provided in Supplementary Table S30.

### 2.5. Network Architectures

To evaluate robustness across diverse model architectures, we considered both convolutional and transformer-based networks. For 2D classification tasks, we employed ResNet-18 [37] and Swin Transformer Tiny [38] models initialized with ImageNet-pretrained weights. For 3D classification, we used R(2 + 1)D − 18 [39] and 3D Swin Transformer [40]. Tiny models initialized with Kinetics-400 pretrained weights [41]. All classification models were implemented using standard architectures through the PyTorch TorchVision framework.

For 2D segmentation, we evaluated a U-Net [42] with an ImageNet-pretrained ResNet-18 encoder and a SegFormer [43] model with an ImageNet-pretrained MiT-B1 backbone, both implemented using the Segmentation Models PyTorch library. For 3D segmentation, we employed a standard 3D U-Net [44] and SwinUNETR [45] implemented through MONAI [46] and trained from randomly initialized weights.

Cross-dataset generalization experiments additionally included both pretrained and randomly initialized variants of ResNet-18, Swin Transformer Tiny, MobileNetV3 (small) [47], and MobileViT-xxs [48], all implemented using standard architectures through the PyTorch TorchVision. The selected network architectures represent foundational and established convolutional and modern transformer-based paradigms commonly used in medical image analysis benchmarks.

### 2.6. 2D and 3D Data Augmentation

For 2D augmentation baselines, we evaluated commonly used transformations, including random horizontal flip, random vertical flip, random rotation, random erasing [49], Cutout [50], CutMix [51], and MixUp [52]. Random flipping and rotation were applied with a fixed application probability (ap = 0.5) consistent with standard practice. All other augmentation methods, including SULBA, were evaluated at two application probabilities (ap = 0.5 and ap = 1.0). Unless otherwise specified, standard implementations and default parameters were used through the PyTorch TorchVision parameters to facilitate reproducibility. Cutout was implemented using a custom implementation following the standard formulation [50].

For 3D experiments, volumetric augmentations, including spike noise and gamma adjustment [24], anisotropy [53], bias field distortion [54], elastic deformation [55], blurring [56], ghosting [57], random flipping, and additive noise, were implemented using standard transformations through the TorchIO library. All transformations were evaluated at application probabilities of 0.5 and 1.0. Augmentation parameters for all baseline methods were set to their standard, widely used defaults from PyTorch TorchVision and TorchIO libraries to simulate realistic out-of-the-box usage. However, for elastic deformation, the number of control points (5, 5, 5), maximum displacement (3, 3, 3), and border locking (set to 2) were configured to preserve anatomical plausibility. Traditional spatial transformations (flipping, rotation) were applied with a probability of *p* = 0.5, consistent with standard practice in the field [17].

### 2.7. Training and Implementation Details

Models were trained using AdamW optimization with mixed-precision training. Cross-entropy loss was minimized using standard mini-batch gradient descent with gradient clipping (ℓ_2_ norm = 1.0). Batch sizes were adjusted according to dataset size and data dimensionality. After evaluating multiple learning rates, values that consistently yielded optimal performance over 100 training epochs were selected for both classification $\left(1{\;\times \;10}^{-4}\right)$ and segmentation tasks $1{\;\times 10\;}^{-3}.$ Extending training beyond this point resulted in overfitting and performance degradation. Models were trained using the standard training splits and evaluated on the corresponding test splits provided with each dataset. The model with the highest validation accuracy was retained.

To assess reproducibility, three independent runs were performed on a randomly selected dataset and model architecture across all augmentation techniques using random seeds 1, 42, and 100. After confirming consistent trends, the random seed was fixed to 42 for all subsequent experiments. All experiments were implemented in Python 3.13 using PyTorch 2.7.1+cu126 within Jupyter Notebook 7.3.2. Analysis of the results was performed using SciPy (v1.15.2), Pandas (v2.2.3), scikit-learn (v1.6.1), and NumPy (v2.2.6). Experiments were conducted on an Intel(R) Core(TM) i7-9850H CPU @ 2.60 GHz (2.59 GHz), 32 GB of RAM, and an NVIDIA Quadro RTX 3000 GPU (6 GB VRAM).

### 2.8. Evaluation Protocol and Statistical Analysis

The primary objective of this study is to identify a robust, high-performing default augmentation strategy across the highly heterogeneous medical imaging domain. Therefore, our evaluation and analysis focus on aggregate performance profiling and comparative ranking. Performance was evaluated using task-standard metrics. For classification, we report accuracy, sensitivity, specificity, area under the receiver operating characteristic curve (AUROC), and F1-score. For segmentation, we report intersection over union (IoU), precision, recall, and F1-score.

To enable holistic comparison across models, architectures, and datasets, we adopted a cumulative ranking system based on aggregated metric performance [17], with minor modifications. This approach reduces sensitivity to outliers and provides a clear, unified order of performance. The cumulative score was computed as

(5) $$C=\sum_{M=1}^{n}Technique(M)$$

where “C” is the cumulative score, “M” is the evaluation metric, “n” is the total number of evaluation metrics, and “technique” is the data augmentation method.

Relative improvement over a non-augmented baseline was calculated as

(6) Relative Improvement=cumulative technique score−cumulative baseline score

We computed 95% confidence intervals for the mean improvements in classification and segmentation benchmarks using the standard error of the mean across datasets. To evaluate cross-dataset generalization, we calculated a composite score by taking the arithmetic mean of standard performance metrics, including accuracy, sensitivity, specificity, AUROC, IoU, and F1-score. This approach was used to assess the overall reliability and ranking of methods across a broad experimental landscape, directly measuring each method’s central effect size and its variability across diverse tasks.

Training overhead was assessed by measuring training time for the selected augmentation strategy over 100 training epochs relative to a non-augmented baseline. The relative time overhead was calculated as:

(7) $$\mathrm{Overhead}=\overset{n}{\underset{n\;=\;1}{\Sigma }}(\overset{100}{\underset{e\;=\;1}{\Sigma }}\mathrm{Technique}\;-\;\overset{100}{\underset{e\;=\;1}{\Sigma }}\mathrm{Baseline})$$

where “*n*” is the total number of experiments, “e” is the epochs, “technique” is a data augmentation technique, and “baseline” is a non-augmented baseline.

Statistical significance of performance differences was determined using paired *t*-tests, with *p* ≤ 0.05 considered statistically significant.

## 3. Results

### 3.1. Benchmark Performance on 2D Medical Image Classification

To evaluate the efficacy of SULBA in 2D medical image classification, we performed an extensive benchmark across ten diverse 2D medical image datasets from the MedMNISTv2 suite, spanning dermatology, pathology, radiology, and histology. Performance was assessed using two widely adopted model architectures—a convolutional neural network (ResNet-18) and a vision transformer (Swin Transformer Tiny)—both initialized with ImageNet-pretrained weights. A baseline model trained without data augmentation served as the reference for all comparisons. For all augmentation experiments, transformations were applied stochastically with the predefined application probability. SULBA and four conventional augmentation techniques—CutMix, Cutout, MixUp, and random erasing—were implemented with application probabilities ap = 0.5 and ap = 1.0 while commonly used spatial augmentations, including rotation, horizontal flip, and vertical flip, were implemented with ap = 0.5, consistent with standard practice.

#### 3.1.1. SULBA Provides Robust Performance Gains Across Diverse Datasets and Model Architectures

**Figure 2:**
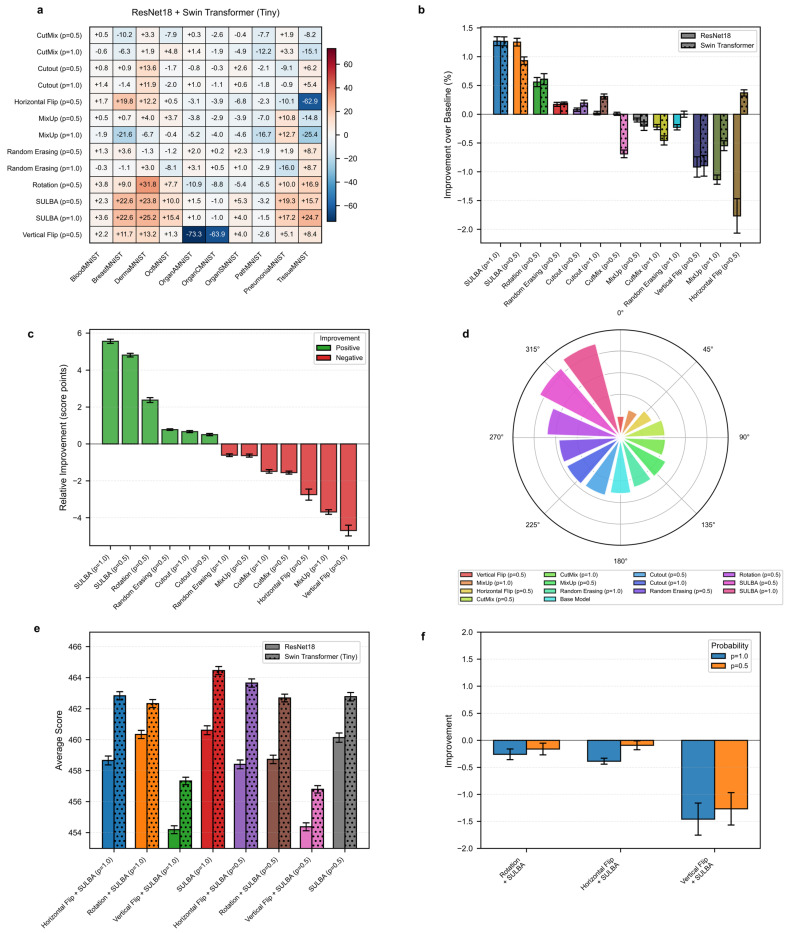
2D medical image classification benchmark. (**a**) Heatmap of the aggregated performance improvement for each data augmentation method relative to a non-augmented baseline across ten 2D MedMNIST datasets. (**b**) Percentage improvement over the baseline for ResNet−18 (solid bars) and Swin Transformer (hatched bars) architectures. (**c**) Mean relative improvement for each method across all datasets and architectures. (**d**) Overall performance ranking based on the sum of scores across all datasets and architectures. (**e**) Performance comparison of SULBA variants and their combinations with traditional spatial augmentations. (**f**) Impact on performance (percentage change) when combining SULBA with traditional techniques.

The aggregate improvement heatmap (Figure 2a) indicates that SULBA (ap = 1.0) achieved positive performance gains in 8 out of 10 datasets when averaged across both model architectures, ranking highest among all evaluated augmentation techniques. Only OrganCMNIST (−0.99) and PathMNIST (−1.54) exhibited modest declines. Notably, SULBA was not uniquely disadvantaged but was among the most robust methods, demonstrating its superior failure tolerance. These declines likely stem from inherent dataset challenges, such as severe class imbalance, which constrain any augmentation technique operating on the existing sample distribution. Specifically, large improvements were observed on DermaMNIST (+25.16), PneumoniaMNIST (+17.16), and TissueMNIST (+24.71) (Supplementary Tables S1–S7). Overall, SULBA (ap = 1.0) attained a mean relative improvement of +5.56 score points over the non-augmented baseline (95% CI ± 2.61), the highest among all evaluated augmentation methods (Figure 2c). In a per-architecture analysis, SULBA (ap = 1.0) yielded the strongest mean percentage improvement for both ResNet-18 (+1.27%, 95% CI ± 0.88) and the Swin Transformer (+1.27%, 95% CI ± 0.89), followed by SULBA (ap = 0.5). Among competing techniques, random rotation achieved the best performance, with mean percentage improvements of +0.56% (95% CI ± 0.91) for ResNet-18 and +0.61% (95% CI ± 1.10) for the Swin Transformer. Collectively, these results demonstrate the robustness of SULBA across both convolutional and attention-based architectures (Figure 2b).

#### 3.1.2. SULBA Demonstrates Superior and Consistent Performance Improvements

The benchmark analysis revealed that SULBA variants ranked highest among all tested data augmentation methods in the aggregate performance ranking. Across all datasets and both model architectures, SULBA (ap = 1.0) achieved the top cumulative score (9355.24), followed closely by SULBA (ap = 0.5) with a score of 9340.43 (Figure 2d; Supplementary Table S10). The performance margin between SULBA (ap = 1.0) and the strongest conventional augmentation, rotation (ap = 0.5), was 63 points, increasing to 95 points relative to the runner-up method (random erasing, ap = 0.5) and 205 points compared with the lowest-performing technique (vertical flip). Importantly, both SULBA variants consistently yielded positive performance gains across architectures, whereas several standard augmentations, including MixUp (ap = 1.0) and horizontal flip (ap = 0.5), exhibited negative mean relative improvements (Figure 2c), underscoring SULBA’s robustness and reliability.

#### 3.1.3. The Integration of SULBA with Traditional Augmentations Does Not Confer Synergistic Benefits

We investigated whether combining SULBA with foundational spatial transforms (horizontal flip, vertical flip, rotation) could yield complementary effects. Contrary to expectation, these combinations consistently underperformed compared to SULBA applied alone (Figure 2e,f). For instance, combining SULBA (ap = 1.0) with horizontal flip resulted in a mean performance decrease of 0.39% compared to standalone SULBA, while combinations with vertical flip showed a more pronounced detrimental effect (−1.46%) (Figure 2f; Supplementary Tables S8 and S9). This suggests that SULBA’s learned, saliency-guided transformations may subsume or conflict with the benefits of heuristic, label-agnostic spatial modifications, establishing SULBA as a performant standalone augmentation strategy.

### 3.2. Benchmark Performance on 3D Medical Image Classification

We extended our benchmark to the 3D domain using six volumetric medical imaging datasets from the 3D MedMNISTv2 (AdrenalMNIST, FractureMNIST, NoduleMNIST, OrganMNIST, SynapseMNIST, VesselMNIST) suite. The evaluation included nine volumetric augmentation techniques pertinent to 3D data, such as anisotropic scaling, bias field simulation, 3D elastic deformation, blurring, noise injection, bias field transformation, flipping and ghost artifacts alongside the baseline and SULBA. Each DA method was assessed with R(2 + 1)D − 18 and 3D Swin Transformer models, both initialized with Kinetics-400 natural video dataset pretrained weights. The selected augmentation strategies were implemented with application probabilities of 0.5 and 1.0.

#### 3.2.1. SULBA Delivers Consistent and Exceptionally Large Improvements Across All 3D Datasets

**Figure 3:**
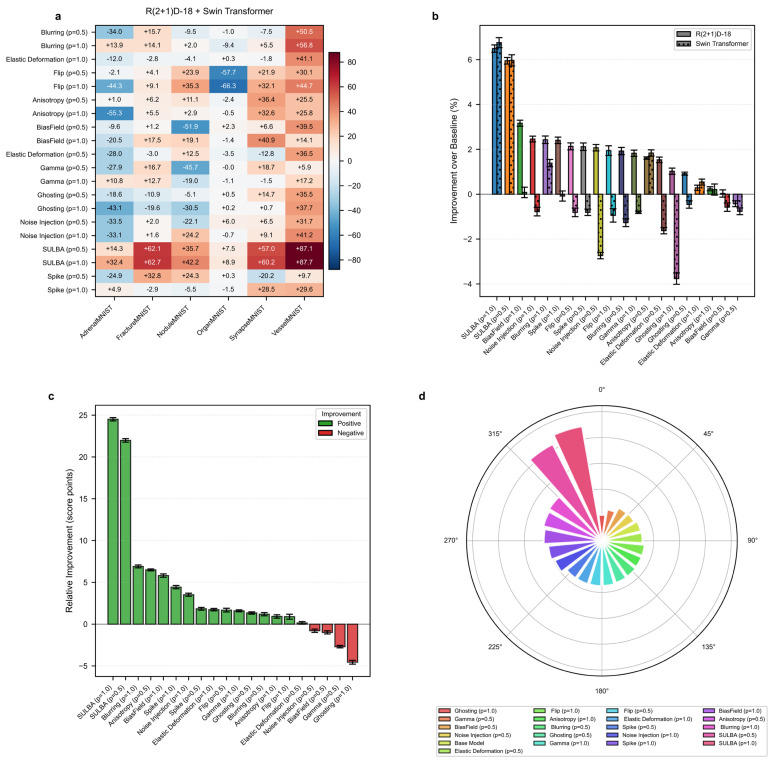
3D medical image classification benchmark. (**a**) Heatmap of the aggregated performance improvement for each volumetric augmentation method across six 3D MedMNIST datasets. (**b**) Percentage improvement for R(2 + 1)D − 18 and 3D Swin Transformer architectures. (**c**) Mean relative improvement for each method. (**d**) Circular ranking plot of the total aggregated score for each method.

The improvement heatmap (Figure 3a) reveals that SULBA (ap = 1.0) provided the highest positive gains for all datasets. The improvements were most pronounced on VesselMNIST (+87.73), FractureMNIST (+62.67), and SynapseMNIST (+60.24). The mean relative improvement for SULBA (ap = 1.0) was +24.52 score points (95% CI ± 9.45), surpassing all other DA techniques (Figure 3c). Architecturally, SULBA (ap = 1.0) showed robust gains for both backbones, with a mean percentage improvement of +6.49% (95% CI ± 3.29) for R(2 + 1)D − 18 and +6.76% (95% CI ± 4.42) for the 3D Swin Transformer (Figure 3b).

#### 3.2.2. Traditional 3D Augmentations Show High Dataset-Specific Variance and Inconsistent Effects

While techniques like blurring and anisotropy provided moderate aggregate benefits, their effects varied widely—and sometimes severely negatively—across datasets. Other techniques demonstrated overall degradation, such as flipping (ap = 1.0) on OrganMNIST (−66.26) (Figure 3a; Supplementary Tables S11–S14). In contrast, SULBA’s saliency-guided approach generated uniformly high, positive impacts, underscoring its generalizability and reliability for 3D medical image analysis.

#### 3.2.3. SULBA Substantially Outperforms Standard Volumetric Augmentation Techniques in 3D Classification

In the aggregate performance ranking across 3D classification benchmarks, SULBA (ap = 1.0) achieved the highest cumulative score (5011.45), followed closely by SULBA (ap = 0.5) with a score of 4980.96 (Figure 3d; Supplementary Tables S15 and S16). Both SULBA variants outperformed all conventional volumetric augmentation techniques. SULBA (ap = 1.0) exceeded the best-performing traditional method (blurring, ap = 1.0) by 211 points and the lowest-performing technique (ghosting, ap = 1.0) by 348 points. These results highlight SULBA’s pronounced and consistent superiority in the volumetric classification setting.

### 3.3. Benchmark Performance on 2D Medical Image Segmentation

To validate SULBA’s efficacy on dense prediction tasks, we benchmarked against seven augmentation methods across seven diverse 2D medical image segmentation datasets from the MedSegBench using both CNN (U-Net with an ImageNet-pretrained ResNet-18 encoder) and Transformer (SegFormer model with an ImageNet-pretrained MiT-B1 backbone) segmentation architectures. Standard data augmentation techniques were evaluated at application probabilities ap = 0.5 and ap = 1.0. Flipping and rotation techniques were evaluated at ap = 0.5.

#### 3.3.1. SULBA Provides Robust, Positive Improvements Across Diverse Segmentation Datasets

**Figure 4:**
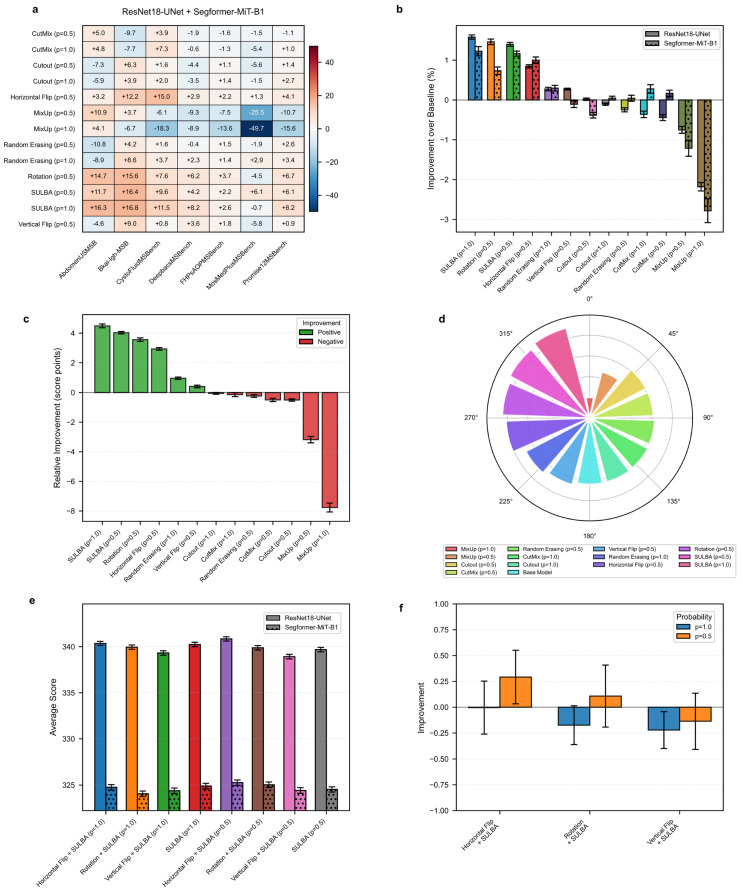
2D medical image segmentation benchmark. (**a**) Heatmap of performance improvement for each augmentation method across seven 2D segmentation datasets. (**b**) Architecture-specific percentage improvement for a ResNet−18-based U-Net and SegFormer. (**c**) Mean relative improvement for each method. (**d**) Overall performance ranking of all methods. (**e**) Performance of SULBA variants and their combinations with traditional augmentations. (**f**) Impact on performance from these combinations.

The improvement heatmap shows SULBA (ap = 1.0) delivered strong gains on AbdomenUSMSBench (+16.28), Bkai-Igh-MSBench (+16.83), and CystoFluidMSBench (+11.47) (Figure 4a). The mean relative improvement for SULBA (ap = 1.0) was +4.49 score points (95% CI ± 2.15), surpassing all other methods (Figure 4c). Architecturally, SULBA showed consistent gains for both backbones, with mean percentage improvements of +1.58% (ResNet-18, 95% CI ± 0.64) and +1.22% (Segformer, 95% CI ± 1.39) (Figure 4b; Supplementary Table S21).

#### 3.3.2. SULBA Ranks as the Top-Performing Augmentation Strategy for 2D Segmentation

In the aggregate performance ranking across seven 2D segmentation datasets and two model architectures, SULBA (ap = 1.0) achieved the highest cumulative score (4655.95), closely followed by SULBA (ap = 0.5) (4649.50) (Figure 4d; Supplementary Table S22). SULBA (ap = 1.0) outperformed the best-performing conventional augmentation (rotation) by 13 points, exceeded the runner-up method (horizontal flip) by 21 points, and surpassed the weakest-performing technique (MixUp, ap = 1.0) by 171 points. Notably, SULBA and its variant were among only three augmentation methods that improved performance in more than 85% of dataset-architecture combinations, alongside random erasing and rotation (Supplementary Tables S17–S20). This consistent superiority demonstrates that SULBA’s advantages extend robustly beyond classification to the more challenging task of dense, pixel-wise segmentation.

#### 3.3.3. Combining SULBA with Spatial Augmentations Provides Marginal and Inconsistent Benefits

When SULBA was combined with traditional spatial augmentations, the effects were small and varied by probability setting. For SULBA (ap = 0.5), pairing with horizontal flip yielded a slight improvement of +0.29%, and with rotation +0.11%, while vertical flip resulted in a minor decline of –0.14%. In contrast, SULBA (ap = 1.0) combinations led to small decreases across all spatial transforms: horizontal flip (–0.004%), rotation (–0.17%), and vertical flip (−0.22%) (Figure 4f).

The magnitude of these changes was minimal, all below 0.30% and inconsistent across probability settings, indicating no reliable synergistic gain (Supplementary Tables S21 and S22). These results reinforce that SULBA alone provides near-optimal augmentation for 2D segmentation, with traditional spatial transforms offering little complementary benefit. This observation further simplifies pipeline design by eliminating the need to combine multiple augmentation strategies.

### 3.4. Benchmark Performance on 3D Medical Image Segmentation

We evaluated SULBA on 3D segmentation tasks using three volumetric datasets: IXITiny, the Medical Segmentation Decathlon (MSD) comprising the MSD-Heart dataset, and the MSD-Hippocampus dataset. The benchmark included nine volumetric augmentation techniques spanning geometric transformations, intensity perturbations, and artifact-related augmentations, each applied with application probabilities of ap = 0.5 and 1.0. Experiments were conducted using two representative and randomly initialized segmentation architectures: a convolutional 3D U-Net and the transformer-based SwinUNETR. A baseline model trained without augmentation was used as the reference for all comparisons.

#### 3.4.1. SULBA Delivers Consistent Improvements Across 3D Segmentation Datasets

**Figure 5:**
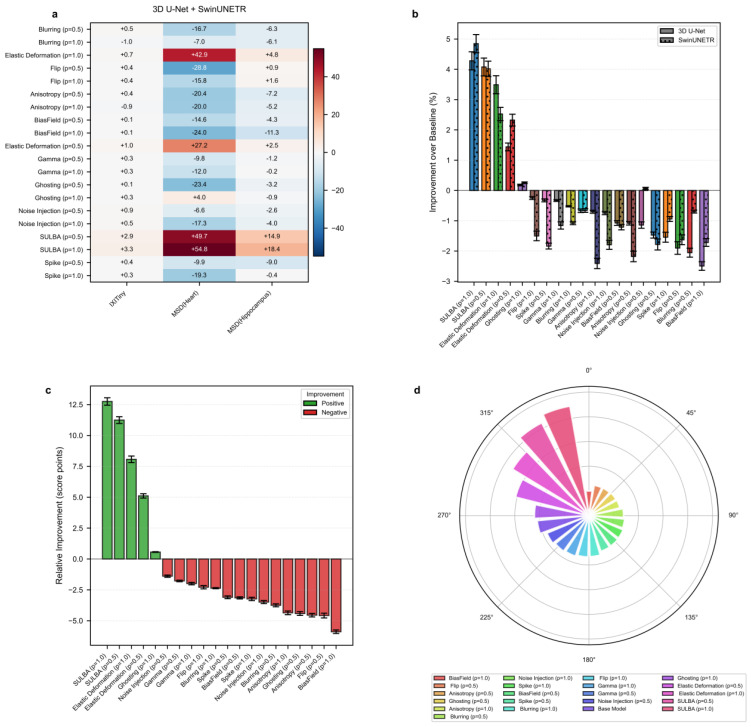
3D medical image segmentation benchmark. (**a**) Heatmap of performance improvement for each volumetric augmentation method across three 3D segmentation datasets. (**b**) Percentage improvement for 3D U−Net and SwinUNETR architectures. (**c**) Mean relative improvement for each method. (**d**) Overall performance ranking based on total aggregated score.

The improvement heatmap shows that SULBA (ap = 1.0) provided the highest gains on all three datasets, with particularly pronounced improvements on MSD-Heart (+54.82) and MSD-Hippocampus (+18.44) (Figure 5a). The mean relative improvement for SULBA (ap = 1.0) was +12.75 score points (95% CI ± 9.56), resulting in a 4.68 improvement over the best competing method (elastic deformation, ap = 1.0) (Figure 5c). Architecturally, SULBA demonstrated consistent benefits, with mean percentage improvements of +4.28% (95% CI ± 5.89) for 3D U-Net and +4.85% (95% CI ± 5.91) for SwinUNETR (Figure 5b).

#### 3.4.2. Conventional 3D Augmentation Methods Exhibit Pronounced Dataset-Dependent Variability

Many established volumetric augmentation techniques produced highly variable and often negative effects across datasets and model architectures. On the MSD-Heart dataset, all augmentation method-probability combinations reduced performance except SULBA and elastic deformation, with several techniques inducing relative declines exceeding 28 points. Although certain methods, such as elastic deformation, yielded moderate improvements on specific datasets, their effects were inconsistent and strongly dependent on both the dataset and the applied probability (0.5 or 1.0) (Figure 5a; Supplementary Tables S23 and S24). Comparable instability was observed on IXITiny and MSD-Hippocampus, where performance gains varied widely across augmentation strategies and experimental settings. Together, these findings highlight the limited reliability of conventional 3D augmentation methods, which may improve performance in isolated cases but frequently degrade it in others, underscoring the need for more robust and task-agnostic augmentation strategies.

#### 3.4.3. SULBA Achieved the Highest Overall Ranking Among 3D Augmentation Strategies

Across all three volumetric datasets and both evaluated architectures, SULBA consistently delivered positive performance gains and achieved the strongest overall results. SULBA (ap = 1.0) attained the highest cumulative score (2023.55), followed closely by SULBA (ap = 0.5) with a score of 2014.51. The leading SULBA variant outperformed the best competing conventional method, random elastic deformation (ap = 1.0), by 28 points; exceeded the runner-up technique, random ghosting (ap = 1.0), by 73 points; and surpassed the lowest-performing method, bias field (ap = 1.0), by 111 points (Figure 5d; Supplementary Tables S25 and S26). Collectively, these results establish SULBA as the most effective and robust augmentation strategy among all evaluated 3D methods, delivering reliable improvements across datasets and architectural paradigms.

### 3.5. Generalization Performance Across Diverse Architectures

**Figure 6:**
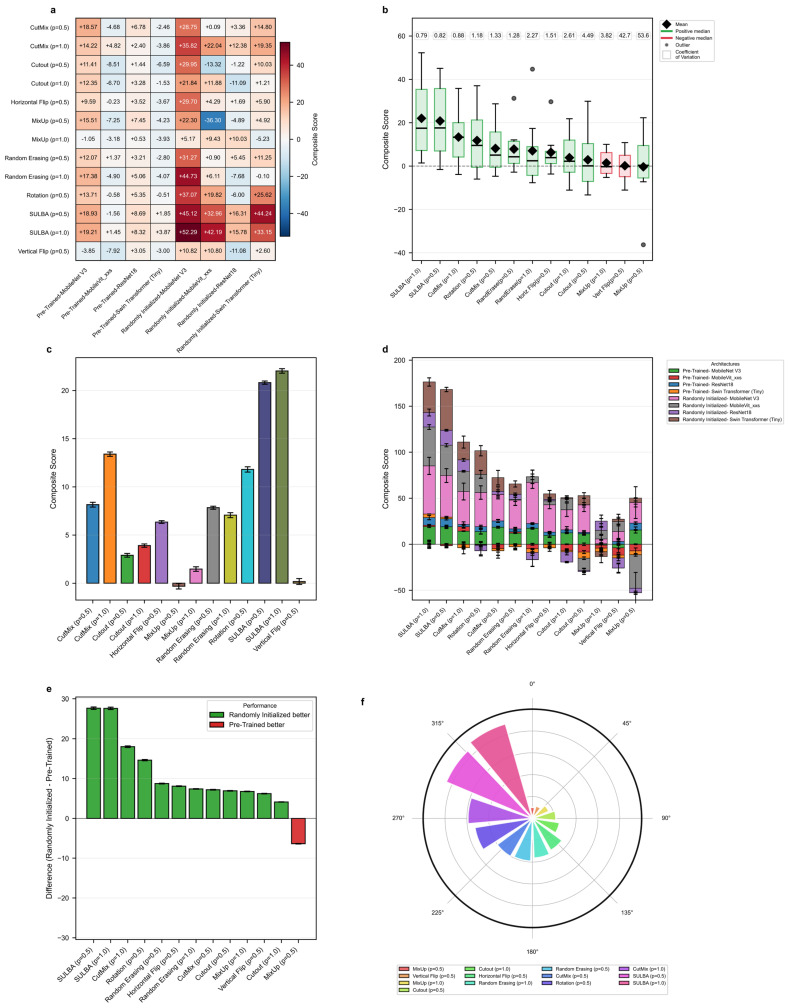
Cross−dataset generalization benchmark. (**a**) Heatmap of the composite performance score for each augmentation method across eight neural network architectures in a pneumonia classification task. (**b**) Distribution of composite scores for each method (box plots) with annotated coefficient of variation (CV). (**c**) Mean composite score for each method. (**d**) Contribution of each architecture to the total composite score for each method. (**e**) Performance difference between randomly initialized and pretrained models for each method. (**f**) Circular ranking plot of the total composite score for each method.

To evaluate SULBA’s ability to generalize beyond the training distribution, models were trained on PneumoniaMNIST and tested on an independent chest X-ray pneumonia dataset [36]. Seven augmentation strategies (CutMix, Cutout, MixUp, random erasing, horizontal flip, rotation, and vertical flip) were benchmarked on four architectures (ResNet-18, Swin Transformer (Tiny), MobileNet V3 (small variant), and MobileViT_xxs), each implemented with both randomly initialized and ImageNet-pretrained weights. Traditional augmentations were applied at application probabilities of 0.5 and 1.0, while spatial augmentations used a fixed probability of 0.5, consistent with standard practice. Comparisons were made across all selected data augmentation strategies across all eight model variants (Figure 6; Supplementary Tables S27 and S28).

#### 3.5.1. SULBA Delivers Superior Cross-Dataset Generalization

SULBA demonstrated robust and consistent cross-dataset generalization across all eight evaluated model variants, achieving positive composite gains in every case (Figure 6a). The largest improvements were observed for randomly initialized models, including MobileNet V3 (+52.29) and Swin Transformer (+33.15) (Supplementary Table S28). Overall, SULBA (ap = 1.0) achieved the highest mean composite score (22.03; 95% CI ± 12.92), an 8.63 improvement over the best competing method, CutMix (ap = 1.0; 13.40; 95% CI ± 8.71) (Figure 6c). In the cumulative performance ranking, SULBA (ap = 1.0) attained the highest total score (3703.84), followed closely by SULBA (ap = 0.5) with a score of 3694.12, outperforming all seven evaluated augmentation strategies across both application probabilities. The leading SULBA variant exceeded the strongest competing method, CutMix (ap = 1.0), by 69 points, rotation by 82 points, and the lowest-performing method, MixUp (ap = 0.5), by 179 points (Figure 6f; Supplementary Table S29). Notably, while conventional augmentations exhibited negative or inconsistent effects when applied to pretrained models, SULBA maintained uniformly positive gains across both randomly initialized and pretrained training regimes, underscoring its reliability for cross-dataset generalization as well as for models trained de novo.

#### 3.5.2. SULBA Provides Consistent Improvements Across Architectures

SULBA demonstrated remarkably stable performance across architectural families, with low variability quantified by a coefficient of variation (CV) of 0.79 for SULBA (ap = 1.0), the lowest among all methods (Figure 6b). In contrast, conventional augmentations exhibited high instability. Vertical flip (ap = 0.5) demonstrated a CV of 42.67, and MixUp (ap = 0.5) showed a CV of 53.63, reflecting unpredictable and frequent detrimental effects on individual architectures (Figure 6b; Supplementary Tables S27 and S28). SULBA’s consistency, coupled with its uniformly positive contributions across both randomly initialized and pretrained models (Figure 6d), underscores its reliability as an architecture-agnostic augmentation strategy.

#### 3.5.3. Training with Randomly Initialized Weights Amplifies SULBA’s Benefits

Analysis of training paradigms revealed that SULBA delivered substantially larger gains in models trained with randomly initialized weights than in those initialized with ImageNet-pretrained weights. The performance increase in randomly initialized models over pretrained models reached +27.68 for SULBA (ap = 0.5) and +27.64 for SULBA (ap = 1.0), the largest differential observed among all augmentation methods (Figure 6e). This advantage was consistent across architectural families, with the randomly initialized variants of MobileNet V3 (+52.29) and Swin Transformer (+33.15) showing the most pronounced absolute improvements under SULBA (ap = 1.0) (Figure 6a). In contrast, traditional augmentation methods such as MixUp and vertical flip exhibited inconsistent or negative effects in the pretrained setting, further highlighting SULBA’s reliability (Supplementary Table S27). Together, these results underscore SULBA’s particular value in data-scarce regimes or when transfer learning from natural images is infeasible or suboptimal for the target medical domain.

### 3.6. Average Training Time Overhead

To evaluate the practical efficiency of SULBA, we measured the average training time overhead over 100 epochs for all augmentation methods relative to a non-augmented baseline. This analysis encompassed both 2D and 3D classification and segmentation benchmarks.

#### 3.6.1. Average Training Time of 2D Augmentation

**Figure 7:**
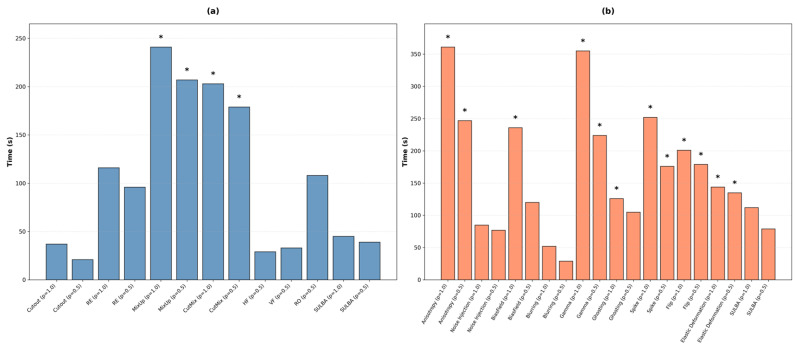
Comparative training time overhead of augmentation strategies. (**a**) Training time overhead for 2D augmentation techniques. (**b**) Training time overhead for 3D volumetric augmentation techniques. * indicates statistical significance (*p* < 0.05).

The mean training time overhead for 2D augmentation techniques is summarized in Figure 7a. Among the evaluated methods, SULBA introduced minimal computational cost. SULBA (ap = 1.0) incurred an average overhead of 45 s per 100 epochs, while SULBA (ap = 0.5) incurred 39 s. These values were among the lowest recorded, comparable to the overhead of simple spatial transforms like horizontal flip (29 s) and significantly lower than that of resource-intensive methods such as MixUp (ap = 1.0; 241 s) and CutMix (ap = 1.0; 203 s). The statistical analysis results indicated that only CutMix (ap = 1.0 (*p* = 0.007) and ap = 0.5 (*p* = 0.018)) and MixUp (ap = 1.0 (*p* = 0.021) and ap = 0.5 (*p* = 0.028)) showed statistical significance compared to the non-augmented baseline. The average training time overhead for SULBA and other lightweight transforms was not statistically distinguishable from the baseline.

#### 3.6.2. Average Training Time Overhead of 3D Volumetric Augmentation

For 3D volumetric data, the computational cost of augmentation was generally higher, as shown in Figure 7b. Consistent with the 2D results, SULBA remained highly efficient. The overhead for SULBA (ap = 1.0) was 112 s per 100 epochs, and for SULBA (ap = 0.5) it was 79 s. In contrast, geometrically complex transformations like anisotropy (ap = 1.0; 361 s) and elastic deformation (ap = 1.0; 355 s) imposed the greatest costs, exceeding SULBA’s average training time overhead. Statistical analysis revealed that transformation methods, including anisotropy (*p* = 0.033), bias field (*p* = 0.002), elastic deformation (*p* = 0.024), ghosting (*p* = 0.016), and spike (*p* = 0.041) at their ap = 1.0 settings, demonstrated a significant increase in average training time relative to the baseline.

## 4. Discussion

Data augmentation is a cornerstone of contemporary deep learning, enhancing model robustness and generalization. In medical imaging, however, no established augmentation approach has demonstrated reliable cross-task, cross-modal, or cross-architectural transferability [18,19,20]. As a result, augmentation pipelines are typically constructed through extensive trial-and-error tuning, which increases experimental burden and undermines reproducibility [13,22]. Our large-scale benchmarks across classification, segmentation, and cross-dataset generalization tasks demonstrate that SULBA offers a simple, deterministic, and parameter-free alternative. Unlike conventional augmentation strategies, SULBA systematically transforms data along intrinsic dimensions without modifying pixel intensities, interpolating content, or altering local structure (Figure 1). Its consistent performance across convolutional and transformer-based architectures, as well as across diverse imaging domains, establishes SULBA as a reliable default augmentation strategy that removes the need for task-specific parameter tuning and directly addresses long-standing inefficiencies in medical data augmentation practice.

Relative to existing concepts, such as permutation-based augmentations and equivariant learning. General random permutation or patch-shuffling techniques often introduce biologically implausible discontinuities, violating the structural consistency required in medical images [17]. SULBA avoids this by employing a structured, whole-axis cyclic shift that guarantees the preservation of all local neighborhoods and tissue continuities within each transformed view. Compared to methods designed for equivariant representation learning, which impose architectural constraints, SULBA provides a data-centric regularization that is compatible with any standard network. Its deterministic and reversible nature ensures no loss or synthetic generation of image content, addressing a core limitation of many generative and corruption-based augmentation methods in the clinical domain [19]. These properties present SULBA as a principled, domain-aware operationalization of cyclic transformations, tailored to the constraints and requirements of medical image analysis. Consequently, a user can apply SULBA as a default augmentation without conducting a hyperparameter search. Furthermore, SULBA’s contribution lies in the demonstration that its structured application forms a universally effective and reliable augmentation framework for medical imaging.

Conventional augmentation approaches typically rely on stochastic deformation, interpolation, partial occlusion, or content mixing [49,50,51,52,53,54,55,56]. While such methods can improve performance in specific settings, their effectiveness is often highly sensitive to anatomical orientation, acquisition protocol, label structure, and model architecture [12,13]. Mixing-based techniques may generate biologically implausible tissue combinations, while aggressive cropping or erasure risks removing clinically salient regions [17], and widely adopted geometric transformations can disrupt critical spatial relationships [26]. Consequently, prior studies have emphasized that augmentation performance is difficult to predict a priori and frequently requires dataset-specific tuning [57,58]. Consistent with these observations, our benchmarks reveal substantial dataset-dependent variability among conventional methods (Figure 2, Figure 3, Figure 4 and Figure 5). In contrast, SULBA exhibits stable performance across architectures, dimensionalities (2D and 3D), and task types (classification and segmentation), indicating that its benefits do not depend on narrow inductive biases but instead arise from a more general, architecture-agnostic regularization mechanism.

The robustness of SULBA stems from its mechanistic design. By applying cyclic shifts along data dimensions, SULBA systematically repositions existing image content while preserving pixel information (Equation (1)). Repeated application across training epochs exposes models to structured yet diverse views of the same underlying sample, reinforcing feature representations without introducing artifacts or synthetic content. This encourages distributed, position-tolerant feature learning and yields consistent gains across both classification and segmentation tasks. In segmentation tasks, exact voxel-level correspondence between inputs and labels is preserved, enabling reliable dense prediction without auxiliary heuristics. Experimental analysis of shift offsets further demonstrates that even a limited number of cyclic shifts can generate sufficient diversity to improve model generalization, particularly for small or low-resolution volumetric inputs (e.g., 3D benchmark using 32 × 32 × 32 voxel images; Figure 5).

SULBA’s impact is most pronounced in data-scarce regimes, including training from random initialization without pretrained representations (Figure 5b and Figure 6e). In such settings, early feature learning is especially vulnerable to spurious correlations and dataset-specific biases due to the absence of strong inductive priors [59,60]. By systematically reconfiguring input structure, SULBA mitigates this vulnerability and promotes the learning of position-invariant features that generalize beyond the training distribution. Across cross-dataset evaluations, SULBA consistently improved performance on independent test sets, indicating enhanced robustness under distributional shift. In the generalization experiment, SULBA-augmented models achieved nearly twofold higher mean composite scores compared with competing augmentation strategies across four architectures trained without pretrained representation, spanning both convolutional and transformer-based models (Figure 6c). Similarly, on 3D volumetric datasets such as MSD-Heart and IXITiny, SULBA produced uniformly strong gains for both 3D U-Net and SwinUNETR models initialized with random weights (Figure 5a). Together, these results indicate that SULBA regularizes early feature learning and mitigates the risks associated with limited labeled data across modalities and dimensionalities.

Across extensive benchmarks, SULBA demonstrated highly stable performance under diverse experimental settings (Supplementary Tables S10, S16, S22 and S26). Performance remained robust to the choice of application probability, with both evaluated settings yielding consistent gains (Figure 2, Figure 3, Figure 4 and Figure 5). When combined with conventional spatial transformations, it maintained high performance. However, these combinations provided no systematic advantage in classification tasks and only modest, dataset-specific improvements in select 2D segmentation benchmarks. These results suggest that SULBA’s intrinsic transformation accounts for the dominant augmentation effect (Figure 2e and Figure 4e). Supporting this interpretation, cross-dataset analyses revealed that SULBA exhibited the lowest coefficients of variation across all evaluated architectures, irrespective of whether models were trained de novo or with pre-activated weights (Figure 6b). This low variability underscores SULBA’s predictability and reliability as a default augmentation strategy, beyond improvements in mean performance alone.

The quantitative analysis of computational overhead (Section 3.6) provides empirical validation that SULBA is a computationally lightweight framework. Its core operation, a deterministic, non-interpolative cyclic shift, is fundamentally cheaper than transformations requiring intensive pixel-level computation, such as interpolation for elastic deformation or blending for mixing-based techniques. This mechanistic efficiency is reflected in the results, where the per-epoch training time overhead for SULBA was negligible and statistically indistinguishable from the non-augmented baseline compared to the significant runtime penalties incurred by resource-intensive methods such as CutMix, MixUp, elastic deformation, etc. (Figure 7). Furthermore, SULBA operates as an online, in-place transformation, generating variations on-the-fly during data loading. This design eliminates the need for pre-computation or the storage of augmented samples, thereby introducing zero persistent memory overhead, a critical advantage for processing high-resolution 3D volumes. Consequently, SULBA delivers the robust performance gains demonstrated in Section 3.1, Section 3.2, Section 3.3, Section 3.4 and Section 3.5 with minimal runtime penalty, establishing it not only as an effective but also as an efficient default choice for both 2D and 3D medical imaging deep learning analysis pipelines. Together, these findings position SULBA as a reliable, universal default for data augmentation in medical image analysis with the potential to improve experimental reproducibility and accelerate the development of robust, generalizable, clinically translatable AI models.

Despite these strengths, our evaluation primarily relies on curated, publicly available datasets. Although these datasets span a wide range of modalities, tasks, and architectures, they cannot fully capture the heterogeneity encountered in prospective clinical environments, including site-specific acquisition protocols, scanner variability, and population-level differences [61,62]. Consequently, while the observed gains strongly suggest generalizability, real-world deployment may introduce additional challenges. Future work will therefore focus on multi-institutional validation, longitudinal evaluation, and deployment under heterogeneous clinical conditions to more comprehensively assess robustness and reproducibility in operational settings.

## 5. Conclusions

This study presents SULBA: a simple, parameter-free, and dimension-agnostic framework based on deterministic cyclic shifts designed to directly address the augmentation lottery in medical imaging deep learning. Comprehensive benchmarks across 27 datasets establish SULBA as a consistently top-performing default strategy. It demonstrates robust efficacy across architectures (convolutional and transformer), dimensionalities (2D and 3D), tasks (classification and segmentation), and diverse modalities, all while introducing minimal computational overhead. For practitioners, applying SULBA with a probability of 1.0 offers a reliable, tuning-free starting point, eliminating the need for task-specific augmentation search.

## Data Availability

All datasets used in this study are publicly available. The MedMNIST v2 datasets were obtained from their official public repository. Additional 2D and 3D segmentation datasets, including MSBench datasets, IXITiny and Medical Segmentation Decathlon (MSD) tasks, are accessible from their respective public sources. The independent chest X-ray pneumonia dataset used for cross-dataset generalization is also publicly available. All datasets were accessed from their original repositories. Detailed dataset descriptions, access information, and preprocessing settings are provided in Supplementary Table S31. No new datasets were generated during this study. The SULBA data augmentation code (Version 1.0), including evaluation, benchmark data augmentation implemetation and figure generation, is available at the GitHub repository: https://github.com/Saintcodded/SULBA-Stepwise-Upper-and-Lower-Boundaries-Augmention.git. assessed on 15 January 2026.

## Supplementary Materials

The following supporting information can be downloaded at https://www.mdpi.com/article/10.3390/diagnostics16101546/s1, Tables S1–S10: Benchmark performance on 2D medical image classification. Tables S11–S16: Benchmark performance on 3D medical image classification. Tables S17–S22: Benchmark performance on 2D medical image segmentation. Tables S23–S26: Benchmark performance on 3D medical image segmentation. Tables S27–S29: Generalization performance of data augmentation techniques across diverse neural architectures using the PneumoniaMNIST (training set) and Chest X-ray Pneumonia dataset (test set). Tables S30–S31: Datasets and training details.

## Abbreviations

The following abbreviations are used in this manuscript:

n	Number
C	Cumulative
M	Metric
2D	Two-Dimensional
3D	Three-Dimensional
ap	Application Probability
DA	Data Augmentation
AI	Artificial Intelligent
CNN	Convolutional Neural Network
MSD	Medical Segmentation Decathlon
SULBA	Stepwise Upper and Lower Boundaries Augmentation
AUROC	Area Under the Receiver Operating Characteristic Curve
