# Supplementary material for: SULBA: A Task-Agnostic Data Augmentation Framework for Deep Learning in Medical Image Analysis
Source: Diagnostics (Basel). 2026 May 19;16(10):1546. doi: 10.3390/diagnostics16101546 (PMC13206679; doi:10.3390/diagnostics16101546)
Supplement: Supplementary file 1 [file diagnostics-16-01546-s001.zip › diagnostics-4126732-supplementary.pdf]

SULBA: A task-agnostic data augmentation framework for deep learning in medical image analysis

Authors: Ayomide A. Abe[1,2], Mpumelelo Nyathi[2]

Affiliations

[1]Sefako Makgatho Health Sciences University, Pretoria, South Africa

[2]AureXida

Correspondence: saintcodded@gmail.com

## Supplementary Tables 1-9: Benchmark performance on 2D medical image classification

(a) **DA =** Data Augmentation, **P** = Augmentation probability of 0.5, **BM** = Base Model, **RE** = Random Erasing, **HF** = Random Horizontal Flip, **VF** = Random Vertical Flip, **RO** = Random Rotation (up to 360°), **SULBA** = Stepwise Upper and Lower Boundaries Augmentation, **A** = Accuracy, **Sen** = Sensitivity, **Sp** = Specificity, **AU** = AUROC, **F1** = F1 Score.
(b) Best performing method is represented in bold values
(c) Second best performing method is represented in underlined values

**Supplementary Table 1: Benchmark performance on 2D medical image classification**

**ResNet-18 (Pretrained on ImageNet)**

**(image size = 64 x 64)**

| **DA** | **BreastMNIST** | | | | | **TissueMNIST** | | | | | **PneumoniaMNIST** | | | | |
|---|---|---|---|---|---|---|---|---|---|---|---|---|---|---|---|
| | **A** | **Sen** | **Sp** | **AU** | **F1** | **A** | **Sen** | **Sp** | **AU** | **F1** | **A** | **Sen** | **Sp** | **AU** | **F1** |
| BM | 90.38 | 94.74 | 78.57 | 93.88 | 93.51 | 71.26 | 61.32 | 95.54 | 94.14 | 70.83 | 93.57 | 97.95 | 85.47 | 98.65 | 94.79 |
| Cutout | 91.03 | **98.25** | 71.43 | 93.61 | 94.12 | 72.41 | 63.59 | 95.73 | 94.50 | 72.05 | 93.27 | 98.21 | 85.04 | 98.67 | 94.80 |
| Cutout-p | 91.67 | 96.49 | 78.57 | 92.94 | 94.42 | 72.10 | 62.25 | 95.72 | 94.42 | 71.69 | 92.47 | 98.21 | 82.91 | 98.06 | 94.22 |
| RE | 90.38 | 95.61 | 76.19 | 92.42 | 93.56 | 72.49 | 63.52 | 95.74 | 94.64 | 72.23 | 91.83 | 98.46 | 80.77 | 97.59 | 93.77 |
| RE-p | 92.31 | 96.49 | 80.95 | 89.91 | 94.83 | 72.64 | 63.37 | 95.78 | 94.51 | 72.27 | 93.75 | 96.92 | 88.46 | 97.15 | 95.09 |
| CutMix | 91.67 | 94.74 | 83.33 | 93.55 | 94.32 | 70.30 | 61.38 | 95.37 | 93.94 | 69.80 | 94.07 | 99.23 | 85.47 | 97.86 | 95.44 |
| CutMix-p | 91.67 | 96.49 | 78.57 | 91.21 | 94.42 | 72.00 | 62.11 | 95.65 | 94.47 | 71.36 | 93.75 | **99.49** | 84.19 | 98.74 | 95.21 |
| MixUp | 88.46 | 94.74 | 71.43 | 87.76 | 92.31 | 69.13 | 60.16 | 95.21 | 93.07 | 68.59 | 94.87 | 98.46 | 88.89 | 98.56 | 96.00 |
| MixUp-p | 91.67 | 97.37 | 76.19 | 90.64 | 94.47 | 71.46 | 61.34 | 95.62 | 94.20 | 71.06 | 94.71 | 98.72 | 88.03 | 98.46 | 95.89 |
| HF-p | 92.95 | 96.49 | 83.33 | **94.70** | 95.24 | 72.43 | 63.02 | 95.72 | 94.63 | 72..06 | 92.31 | 98.72 | 81.62 | 98.59 | 94.13 |
| VF-p | 92.31 | 96.49 | 80.95 | 93.11 | 94.83 | 72.48 | 62.61 | 95.70 | 94.71 | 72.03 | 94.07 | 98.72 | 86.32 | 98.45 | 95.42 |
| RO-p | 92.95 | 96.94 | 83.33 | 92.40 | 95.24 | 73.41 | 65.04 | 95.91 | 94.79 | 73.16 | 94.55 | 97.44 | 89.74 | 97.99 | 95.72 |
| SULBA | **94.23** | 96.49 | **88.10** | 92.93 | 96.07 | **74.44** | **66.71** | **96.12** | **95.32** | **74.39** | 95.35 | **99.49** | 88.46 | **99.31** | 96.40 |
| SULBA-p | **94.23** | 97.37 | 85.71 | 93.13 | **96.10** | 73.46 | 64.33 | 95.92 | 94.89 | 73.17 | **95.67** | 98.97 | **90.17** | 98.67 | **96.62** |
| | | | | | | | | | | | | | | | |
| HF-p + SULBA | 93.59 | 96.49 | 85.71 | 94.07 | 95.65 | 74.39 | 64.85 | 96.04 | 95.36 | 73.95 | **95.67** | 97.95 | **91.88** | 98.88 | 96.59 |
| HF-p + SULBA-p | 93.59 | 96.49 | 85.71 | 91.50 | 95.65 | 74.15 | 64.85 | 96.02 | 95.10 | 73.84 | 94.39 | **99.23** | 86.32 | 98.96 | 95.63 |
| VF-p + SULBA | 93.59 | 96.49 | 85.71 | 93.15 | 95.65 | 74.88 | 66.04 | 96.13 | 95.56 | 74.55 | **95.67** | 98.72 | 90.60 | 98.93 | **96.61** |
| VF-p + SULBA-p | **94.23** | 96.49 | **88.10** | 93.09 | 96.07 | 74.54 | 65.90 | 96.08 | 95.33 | 74.25 | 94.39 | 98.21 | 88.03 | 98.94 | 95.67 |
| RO-p + SULBA | **94.23** | 96.49 | **88.10** | 93.57 | 96.07 | **75.70** | **66.61** | **96.23** | **95.67** | **75.33** | 95.03 | 98.97 | 88.46 | **99.06** | 96.14 |
| RO-p + SULBA-p | **94.23** | **97.37** | 85.71 | **94.59** | **96.10** | 75.37 | 66.47 | 96.19 | 95.55 | 75.04 | 93.75 | 97.95 | 86.75 | 98.71 | 95.14 |

**Supplementary Table 2: Benchmark performance on 2D medical image classification**

**Swin Transformer Tiny (Pretrained on ImageNet)**

**(image size = 64 x 64)**

| DA | BreastMNIST | | | | | TissueMNIST | | | | | PneumoniaMNIST | | | | |
|---|---|---|---|---|---|---|---|---|---|---|---|---|---|---|---|
| | A | Sen | Sp | AU | F1 | A | Sen | Sp | AU | F1 | A | Sen | Sp | AU | F1 |
| BM | 93.59 | 97.37 | 83.33 | 94.20 | 95.69 | 73.63 | 64.77 | 95.93 | 95.09 | 73.28 | 94.23 | 98.21 | 87.61 | 98.49 | 95.51 |
| Cutout | 93.59 | 97.37 | 83.33 | 95.43 | 95.69 | 73.71 | 65.00 | 95.98 | 94.81 | 73.44 | 94.87 | 98.46 | 88.89 | 97.99 | 96.00 |
| Cutout-p | 92.95 | 96.49 | 83.33 | 94.04 | 95.24 | 74.48 | 65.86 | 96.05 | 95.26 | 74.14 | 94.55 | 99.23 | 86.75 | 96.58 | 95.79 |
| RE | 93.59 | 96.49 | 85.71 | 94.51 | 95.65 | 74.59 | 65.60 | 96.10 | 95.27 | 74.26 | 94.07 | **99.49** | 85.04 | 98.47 | 95.45 |
| RE-p | 92.95 | 96.49 | 83.33 | **96.32** | 95.24 | 74.30 | 66.16 | 96.08 | 95.35 | 74.05 | 94.07 | 98.21 | 87.18 | 98.59 | 95.39 |
| CutMix | 90.38 | 93.86 | 80.95 | 92.67 | 93.45 | 70.09 | 61.07 | 95.40 | 93.80 | 69.56 | 94.55 | 96.67 | **91.03** | 98.05 | 95.69 |
| CutMix-p | 91.67 | 96.49 | 78.57 | 91.50 | 94.42 | 70.66 | 61.85 | 95..38 | 94.17 | 69.98 | 92.63 | 96.92 | 85.47 | 97.34 | 94.26 |
| MixUp | 92.95 | 97.37 | 80.95 | 92.40 | 95.28 | 68.78 | 58.99 | 95.17 | 93.10 | 68.21 | 92.95 | 96.15 | 87.61 | 96.93 | 94.46 |
| MixUp-p | 93.59 | 97.37 | 83.33 | 95.68 | 95.69 | 69.78 | 59.37 | 95.30 | 93.73 | 69.13 | 93.27 | 97.95 | 85.47 | 95.78 | 94.79 |
| HF-p | **94.87** | 96.49 | **90.48** | 94.05 | 96.49 | 74.66 | 66.62 | 96.10 | 95.38 | 74.36 | 92.47 | 98.72 | 82.05 | 97.98 | 94.25 |
| VF-p | **94.87** | 97.37 | 88.10 | 92.38 | 96.52 | 74.59 | 66.28 | 96.11 | 95.39 | 74.31 | 94.55 | 98.97 | 87.18 | 98.44 | 95.78 |
| RO-p | 93.59 | 93.49 | 85.71 | 94.95 | 95.65 | 75.30 | 67.99 | 96.23 | 95.59 | 75.22 | 93.75 | 99.23 | 84.62 | 98.53 | 95.20 |
| SULBA | **94.87** | **98.25** | 85.71 | 94.70 | **96.55** | **76.36** | **68.66** | **96.40** | **95.92** | **76.18** | 94.87 | 98.72 | 88.46 | 98.53 | 96.01 |
| SULBA-p | **94.87** | 97.37 | 88.10 | 94.45 | 96.52 | 75.50 | 67.18 | 96.20 | 95.62 | 75.20 | **95.35** | 98.97 | 89.32 | **98.99** | **96.38** |
| | | | | | | | | | | | | | | | |
| HF-p + SULBA | 94.87 | 97.37 | 88.10 | 93.63 | 96.52 | 76.28 | 67.89 | 96.33 | 95.88 | 75.00 | 95.19 | 98.46 | 89.74 | 99.14 | 96.24 |
| HF-p + SULBA-p | **95.51** | 97.37 | **90.48** | 95.59 | 96.94 | 75.72 | 67.53 | 96.25 | 95.69 | 75.49 | 94.71 | 98.46 | 88.46 | 98.88 | 95.88 |
| VF-p + SULBA | 94.87 | 96.49 | **90.48** | 95.51 | 96.49 | 76.24 | 67.95 | 96.31 | 95.94 | 75.87 | **95.83** | **99.23** | 90.17 | **99.22** | **96.75** |
| VF-p + SULBA-p | **95.51** | **98.25** | 88.10 | 95.65 | **96.97** | 75.81 | 68.22 | 96.27 | 95.76 | 75.58 | 94.39 | 98.97 | 86.75 | 99.07 | 95.66 |
| RO-p + SULBA | 94.23 | 97.37 | 85.71 | **96.14** | 96.10 | **76.80** | **68.94** | **96.45** | **96.13** | **76.51** | **95.83** | 97.18 | **93.59** | 98.79 | 96.68 |
| RO-p + SULBA-p | 94.87 | 96.49 | **90.48** | 94.48 | 96.04 | 76.60 | 68.73 | 96.40 | 96.03 | 76.34 | 94.87 | **99.23** | 87.61 | 98.57 | 96.03 |

**Supplementary Table 3: Benchmark performance on 2D medical image classification**

**ResNet-18 (Pretrained on ImageNet)**

**(image size = 64 x 64)**

| DA | OrganAMNIST | | | | | OrganCMNIST | | | | | OrganSMNIST | | | | |
|---|---|---|---|---|---|---|---|---|---|---|---|---|---|---|---|
| | A | Sen | Sp | AU | F1 | A | Sen | Sp | AU | F1 | A | Sen | Sp | AU | F1 |
| BM | 97.20 | 96.36 | 99.72 | 99.90 | 97.18 | 95.33 | 94.77 | 99.53 | 99.79 | 95.30 | 84.32 | 79.96 | 98.43 | 98.09 | 84.18 |
| Cutout | 97.38 | 97.00 | 99.74 | 99.89 | 97.38 | 95.03 | 94.33 | 99.50 | 99.77 | 95.02 | 84.28 | 80.04 | 98.43 | 98.04 | 84.14 |
| Cutout-p | 97.02 | 96.35 | 99.70 | 99.93 | 97.00 | 94.96 | 94.27 | 99.50 | **99.80** | 94.93 | 84.51 | 80.48 | 98.45 | 98.03 | 84.30 |
| RE | **97.73** | 97.31 | **99.77** | **99.96** | **97.73** | **95.44** | 94.72 | **99.54** | 99.78 | **95.42** | 84.63 | 80.17 | 98.46 | 98.20 | 84.37 |
| RE-p | 97.55 | 97.05 | 99.75 | 99.95 | 97.54 | 95.24 | 94.59 | 99.53 | 99.79 | 95.24 | 84.54 | 80.08 | 98.44 | 98.13 | 84.09 |
| CutMix | 97.52 | **97.36** | 99.75 | 99.91 | 97.51 | 94.86 | 94.61 | 99.49 | 99.76 | 94.88 | 83.35 | 78.58 | 98.34 | 97.01 | 82.97 |
| CutMix-p | 97.44 | **97.36** | 99.74 | 99.93 | 97.43 | 94.86 | 94.61 | 99.49 | 99.76 | 94.88 | 84.07 | 79.80 | 98.41 | 97.88 | 83.82 |
| MixUp | 95.79 | 94.82 | 99.57 | 99.84 | 95.77 | 94.85 | 94.60 | 99.49 | 99.67 | 94.84 | 82.77 | 78.55 | 98.26 | 97.97 | 82.54 |
| MixUp-p | 95.98 | 95.07 | 99.59 | 99.80 | 95.96 | 94.83 | 94.72 | 99.49 | 99.76 | 94.84 | 83.26 | 79.00 | 98.32 | 98.12 | 83.06 |
| HF-p | 96.54 | 96.06 | 99.65 | 99.88 | 96.52 | 94.62 | 93.93 | 99.46 | 99.54 | 94.62 | 82.85 | 77.85 | 98.27 | 97.55 | 82.63 |
| VF-p | 86.48 | 83.25 | 98.64 | 98.70 | 86.38 | 86.49 | 83.16 | 98.67 | 98.63 | 86.58 | **85.35** | **80.87** | **98.54** | **98.27** | **85.07** |
| RO-p | 95.53 | 94.88 | 99.55 | 99.86 | 95.48 | 93.94 | 93.08 | 99.40 | 99.73 | 93.94 | 82.84 | 78.59 | 98.28 | 97.92 | 82.74 |
| SULBA | 97.25 | 97.27 | 99.72 | 99.89 | 97.23 | 95.13 | 94.63 | 99.52 | 99.79 | 95.13 | 84.66 | 80.49 | 98.47 | 98.26 | 84.54 |
| SULBA-p | 97.53 | 97.18 | 99.75 | 99.92 | 97.53 | 95.31 | **94.89** | 99.53 | 99.73 | 95.30 | 85.30 | 80.80 | 98.53 | 98.15 | 85.02 |
| | | | | | | | | | | | | | | | |
| HF-p + SULBA | 96.70 | **96.39** | 99.67 | **99.92** | 96.69 | **94.28** | **93.69** | **99.43** | 99.68 | **94.25** | 83.77 | 79.30 | 98.37 | 98.05 | 83.30 |
| HF-p + SULBA-p | **96.87** | 96.36 | **99.68** | **99.92** | **96.86** | 94.19 | 93.60 | 99.42 | **99.71** | 94.21 | 84.01 | 79.33 | 98.40 | 98.09 | 83.70 |
| VF-p + SULBA | 87.54 | 85.28 | 98.75 | 98.88 | 87.46 | 86.32 | 82.50 | 98.65 | 98.76 | 85.91 | **85.18** | **80.98** | **98.53** | 98.15 | **85.09** |
| VF-p + SULBA-p | 87.74 | 85.58 | 98.77 | 98.85 | 87.55 | 87.39 | 84.16 | 98.75 | 98.74 | 87.36 | 85.14 | 80.91 | 98.52 | **98.40** | 85.00 |
| RO-p + SULBA | 95.80 | 95.82 | 99.58 | 99.82 | 95.76 | 93.59 | 92.98 | 99.36 | 99.60 | 93.60 | 83.58 | 79.97 | 98.36 | 98.15 | 83.48 |
| RO-p + SULBA-p | 95.90 | 95.67 | 99.59 | 99.84 | 95.86 | 94.02 | 93.46 | 99.40 | 99.64 | 94.03 | 83.77 | 79.84 | 93.38 | 97.97 | 83.71 |

| **Supplementary Table 4: Benchmark performance on 2D medical image classification** | | | | | | | | | | | | | | | |
|---|---|---|---|---|---|---|---|---|---|---|---|---|---|---|---|
| **Swin Transformer Tiny (Pretrained on ImageNet) (image size = 64 x 64)** | | | | | | | | | | | | | | | |
| **DA** | **OrganAMNIST** | | | | | **OrganCMNIST** | | | | | **OrganSMNIST** | | | | |
| | **A** | **Sen** | **Sp** | **AU** | **F1** | **A** | **Sen** | **Sp** | **AU** | **F1** | **A** | **Sen** | **Sp** | **AU** | **F1** |
| BM | 97.75 | 97.60 | 99.77 | 99.93 | 97.75 | 95.96 | 95.35 | 99.60 | 99.80 | 95.95 | 84.97 | 80.18 | 98.49 | 97.90 | 84.75 |
| Cutout | 97.74 | 97.62 | 99.77 | 99.92 | 97.75 | 96.01 | 95.44 | 99.60 | 99.78 | 95.99 | 85.08 | 80.44 | 98.51 | 98.05 | 84.91 |
| Cutout-p | 97.68 | 97.35 | 99.77 | 99.92 | 97.67 | **96.23** | **95.78** | **99.62** | 99.77 | **96.22** | 85.49 | 80.97 | 98.55 | 98.01 | 85.11 |
| RE | **98.02** | **97.98** | **99.80** | **99.95** | **98.02** | 96.05 | 95.55 | 99.60 | 99.78 | 96.01 | 85.31 | 80.47 | 98.44 | **98.40** | 83.83 |
| RE-p | 97.87 | 97.87 | 99.78 | 99.94 | 97.87 | 96.13 | 95.56 | 99.61 | 99.80 | 96.13 | 85.28 | 81.18 | 98.53 | 98.13 | 85.22 |
| CutMix | 97.63 | 97.53 | 99.76 | 99.92 | 97.63 | 95.76 | 95.04 | 99.58 | 99.76 | 95.76 | 85.11 | 80.62 | 98.51 | 98.39 | 83.48 |
| CutMix-p | 97.38 | 97.11 | 99.73 | 99.93 | 97.39 | 95.46 | 94.90 | 99.55 | **99.81** | 95.47 | 85.09 | 80.76 | 98.50 | 98.16 | 84.41 |
| MixUp | 97.55 | 97.35 | 99.75 | 99.94 | 97.54 | 95.11 | 94.55 | 99.51 | 99.66 | 95.11 | 84.77 | 80.61 | 98.48 | 98.19 | 84.53 |
| MixUp-p | 97.59 | 97.44 | 99.76 | 99.90 | 97.59 | 95.41 | 94.93 | 99.54 | 99.62 | 95.39 | 84.57 | 80.42 | 98.45 | 98.11 | 84.08 |
| HF-p | 97.36 | 97.06 | 99.73 | 99.94 | 97.35 | 95.44 | 95.07 | 99.55 | 99.80 | 95.44 | 84.37 | 80.20 | 98.44 | 98.10 | 84.18 |
| VF-p | 87.26 | 84.72 | 98.72 | 98.58 | 87.09 | 86.96 | 83.18 | 98.71 | 98.32 | 86.80 | 85.11 | 80.85 | 98.52 | 97.92 | 84.78 |
| RO-p | 95.89 | 95.78 | 99.59 | 99.86 | 95.87 | 94.62 | 94.05 | 99.46 | 99.80 | 94.58 | 84.35 | 80.47 | 98.44 | **98.40** | 83.83 |
| SULBA | 97.77 | 97.55 | 99.77 | 99.94 | 97.77 | 95.75 | 95.38 | 99.57 | 99.74 | 95.75 | **85.62** | 81.05 | **98.57** | 98.23 | **85.40** |
| SULBA-p | 97.71 | 97.63 | 99.77 | 99.93 | 97.71 | 95.65 | 95.03 | 99.56 | 99.80 | 95.63 | 85.54 | **81.26** | 98.56 | 98.13 | 85.24 |
| | | | | | | | | | | | | | | | |
| HF-p + SULBA | **97.10** | **97.04** | 99.71 | 99.88 | 97.07 | 94.80 | 94.24 | 99.48 | 99.64 | 94.79 | 84.75 | **80.82** | 98.48 | 98.29 | 84.65 |
| HF-p + SULBA-p | **97.23** | 97.03 | **99.72** | **99.93** | **97.21** | **95.18** | **94.80** | **99.52** | 99.61 | **95.18** | **85.11** | 80.56 | **98.52** | 98.28 | **84.89** |
| VF-p + SULBA | 86.92 | 84.27 | 98.69 | 98.71 | 86.75 | 86.81 | 83.25 | 98.70 | 98.76 | 86.75 | 84.80 | 80.42 | 98.48 | 98.19 | 84.64 |
| VF-p + SULBA-p | 86.76 | 84.20 | 98.67 | 98.49 | 86.60 | 86.64 | 82.79 | 98.68 | 98.75 | 86.62 | 84.94 | 80.79 | 98.50 | 98.27 | 84.73 |
| RO-p + SULBA | 96.26 | 95.92 | 99.62 | 99.88 | 96.25 | 94.33 | 93.81 | 99.43 | 99.76 | 94.32 | 84.20 | 80.14 | 98.42 | **98.48** | 83.99 |
| RO-p + SULBA-p | 96.09 | 96.11 | 99.61 | 99.80 | 96.06 | 94.63 | 93.90 | 99.46 | **99.79** | 94.59 | 84.49 | 79.86 | 98.44 | 98.35 | 84.11 |

| **Supplementary Table 5: Benchmark performance on 2D medical image classification** | | | | | | | | | | | | | | | |
|---|---|---|---|---|---|---|---|---|---|---|---|---|---|---|---|
| **ResNet-18 (Pretrained on ImageNet) (image size = 64 x 64)** | | | | | | | | | | | | | | | |
| **DA** | **OctMNIST** | | | | | **DermaMNIST** | | | | | **BloodMNIST** | | | | |
| | **A** | **Sen** | **Sp** | **AU** | **F1** | **A** | **Sen** | **Sp** | **AU** | **F1** | **A** | **Sen** | **Sp** | **AU** | **F1** |
| BM | 91.30 | 91.30 | 97.10 | 98.66 | 91.29 | 82.94 | 66.74 | 95.39 | 94.31 | 82.41 | 98.22 | 98.30 | 99.74 | 99.88 | 98.22 |
| Cutout | 90.30 | 90.30 | 96.77 | 98.72 | 90.11 | 83.09 | 66.82 | 95.48 | 94.62 | 82.66 | 98.51 | 98.74 | 99.78 | 99.92 | 98.51 |
| Cutout-p | 90.90 | 90.90 | 96.97 | 99.28 | 90.79 | 83.04 | 68.40 | 95.48 | 95.14 | 82.63 | 98.39 | 98.49 | 99.76 | 99.91 | 98.39 |
| RE | 90.40 | 90.40 | 96.80 | 99.17 | 90.42 | 81.80 | 64.09 | 95.19 | 94.68 | 81.30 | 98.13 | 98.12 | 99.72 | 99.89 | 98.13 |
| RE-p | 91.30 | 91.30 | 97.10 | 99.35 | 91.37 | 82.59 | 63.17 | 94.98 | 94.53 | 81.61 | 98.48 | 98.55 | 99.77 | 99.88 | 98.48 |
| CutMix | 91.10 | 91.10 | 97.03 | **99.58** | 90.78 | 81.50 | 66.37 | 94.84 | 95.45 | 80..64 | 97.98 | 97.96 | 99.70 | 99.89 | 97.48 |
| CutMix-p | 91.60 | 91.60 | 97.20 | 99.17 | 91.44 | 82.84 | 66.38 | 95.12 | 95.30 | 82.07 | 98.33 | 98.29 | 99.75 | 99.91 | 98.33 |
| MixUp | 91.70 | 91.70 | 97.23 | 98.55 | 91.62 | 81.05 | 61.82 | 94.97 | 94.37 | 80.39 | 97.49 | 97.43 | 99.63 | 99.87 | 97.98 |
| MixUp-p | 91.10 | 91.10 | 97.03 | 98.97 | 91.00 | 83.09 | 68.19 | 95.54 | 94.83 | 82.67 | 98.28 | 98.31 | 99.75 | 99.91 | 98.27 |
| HF-p | 90.30 | 90.30 | 96.77 | 99.14 | 90.27 | 83.39 | 69.67 | 95.24 | 94.58 | 82.59 | 98.51 | 98.47 | 99.78 | 99.89 | 98.51 |
| VF-p | 91.20 | 91.20 | 97.07 | 98.99 | 91.04 | 83.39 | 68.27 | 95.91 | 94.96 | 83.28 | 98.60 | 98.73 | 99.79 | **99.94** | 98.62 |
| RO-p | 91.50 | 91.50 | 97.17 | 98.95 | 91.14 | **85.59** | **70.36** | **96.21** | **96.22** | **85.25** | **98.86** | **98.91** | **99.83** | 99.93 | **98.86** |
| SULBA | 92.80 | 92.80 | 97.60 | 99.27 | 92.70 | **84.49** | 69.66 | 95.66 | 95.48 | 83.85 | 98.74 | 98.81 | 99.81 | 99.92 | 98.74 |
| SULBA-p | **93.60** | **93.60** | **97.87** | 99.21 | **93.51** | 84.29 | **71.30** | 95.80 | 95.82 | 83.94 | 98.51 | 98.53 | 99.78 | 99.92 | 98.51 |
| | | | | | | | | | | | | | | | |
| HF-p + SULBA | 90.20 | 90.20 | 96.73 | 99.12 | 89.92 | 84.64 | 70.19 | 95.96 | 95.97 | 84.41 | 98.68 | 98.83 | 99.80 | 99.94 | 98.69 |
| HF-p + SULBA-p | 91.50 | 91.50 | 97.17 | 99.20 | 91.37 | 83.89 | 72.70 | 96.18 | **97.07** | 83.92 | **98.98** | **99.18** | **99.85** | 99.94 | **98.98** |
| VF-p + SULBA | 90.20 | 90.20 | 96.73 | 98.84 | 91.70 | 84.64 | 70.06 | 95.89 | 96.10 | 84.31 | 98.66 | 98.68 | 99.80 | 99.94 | 98.66 |
| VF-p + SULBA-p | **91.90** | **91.90** | **97.30** | 99.23 | **91.79** | 84.24 | 69.78 | 96.00 | 95.82 | 84.05 | 98.74 | 98.76 | 99.81 | 99.91 | 98.74 |
| RO-p + SULBA | 91.80 | 91.80 | 97.27 | 99.40 | 91.76 | **85.84** | **73.04** | **96.36** | 96.47 | **85.65** | 98.83 | 98.85 | 99.82 | 99.93 | 98.83 |

| | | | | | | | | | | | | | | | |
|---|---|---|---|---|---|---|---|---|---|---|---|---|---|---|---|
| RO-p + SULBA-p | 91.00 | 91.00 | 97.00 | **99.45** | 90.79 | 85.19 | 70.99 | 96.12 | 96.51 | 84.95 | 98.83 | 98.96 | 99.83 | **99.95** | 98.83 |

**Supplementary Table 6: Benchmark performance on 2D medical image classification**

**Swin Transformer Tiny (Pretrained on ImageNet)**
**(image size = 64 x 64)**

| DA | OctMNIST | | | | | MNIST | | | | | BloodMNIST | | | | |
|---|---|---|---|---|---|---|---|---|---|---|---|---|---|---|---|
| | A | Sen | Sp | AU | F1 | A | Sen | Sp | AU | F1 | A | Sen | Sp | AU | F1 |
| BM | 89.20 | 89.20 | 96.40 | 99.05 | 89.34 | 84.79 | 68.86 | 96.03 | 94.97 | 84.43 | 98.51 | 98.61 | 99.78 | 99.94 | 98.51 |
| Cutout | 89.60 | 89.60 | 96.53 | 99.35 | 89.60 | 85.39 | 76.33 | 96.40 | 96.69 | 85.28 | 98.63 | 98.71 | 99.79 | 99.94 | 98.63 |
| Cutout-p | 88.90 | 88.90 | 96.30 | 99.32 | 88.89 | 85.54 | 75.66 | 96.50 | 96.52 | 85.56 | 98.57 | 98.69 | 99.79 | 99.92 | 98.57 |
| RE | 87.70 | 87.70 | 95.90 | 99.00 | 87.23 | 85.54 | 73.03 | 96.35 | 96.61 | 85.29 | 98.48 | 98.73 | 99.77 | **99.95** | 98.48 |
| RE-p | 88.60 | 88.60 | 96.20 | 99.16 | 88.63 | 85.74 | 68.78 | 96.24 | 96.45 | 85.45 | 98.66 | 98.74 | 99.80 | **99.95** | 98.66 |
| CutMix | 90.70 | 90.70 | 96.90 | 99.15 | 90.64 | 84.79 | 72.15 | 96.03 | 96.54 | 84.43 | 98.71 | 98.91 | 99.80 | 99.93 | 98.71 |
| CutMix-p | 86.60 | 86.60 | 95.53 | 99.06 | 86.11 | 84.39 | 72.06 | 95.95 | 96.09 | 83.98 | 98.57 | 98.70 | 99.79 | 99.94 | 98.57 |
| MixUp | 88.90 | 88.90 | 96.30 | 98.55 | 89.00 | 83.14 | 73.11 | 96.11 | 95.96 | 83.23 | 98.57 | 98.71 | 99.79 | 99.83 | 98.56 |
| MixUp-p | 90.50 | 90.50 | 96.83 | 99.23 | 90.29 | 84.04 | 70.33 | 96.06 | 96.13 | 83.97 | 98.60 | 98.72 | 99.79 | 99.94 | 98.60 |
| HF-p | 90.20 | 90.20 | 96.73 | 99.35 | 90.06 | 85.99 | 72.75 | 96.37 | 96.68 | 85.78 | 98.83 | 98.89 | 99.82 | 99.92 | 98.83 |
| VF-p | 89.60 | 89.60 | 96.53 | 99.24 | 89.67 | 85.84 | 73.76 | 96.28 | 96.88 | 85.55 | 98.80 | 98.88 | 99.82 | 99.92 | 98.80 |
| RO-p | 91.30 | 91.30 | 97.10 | 99.29 | 91.30 | 87.48 | **79.98** | 96.77 | 97.40 | 87.40 | 99.04 | 99.20 | 99.86 | **99.95** | 99.04 |
| SULBA | **92.10** | **92.10** | **97.37** | **99.51** | **91.96** | **87.68** | 77.10 | **96.91** | **97.62** | **87.58** | **99.09** | **99.28** | **99.87** | **99.95** | **99.10** |
| SULBA-p | 89.90 | 89.90 | 96.63 | 99.12 | 89.54 | 87.43 | 74.87 | 96.73 | 97.29 | 87.18 | 98.92 | 99.11 | 99.84 | 99.94 | 98.92 |
| | | | | | | | | | | | | | | | |
| HF-p + SULBA | 90.40 | 90.40 | 96.80 | 99.35 | 90.11 | 87.78 | 75.42 | 96.83 | 97.47 | 87.65 | 99.04 | 99.22 | **99.86** | 99.94 | 99.03 |
| HF-p + SULBA-p | **92.60** | **92.60** | **97.53** | **99.67** | **92.49** | 87.13 | 76.78 | 96.78 | 97.19 | 87.07 | 99.06 | **99.24** | **99.86** | 99.92 | 99.06 |
| VF-p + SULBA | 89.30 | 89.30 | 96.43 | 99.39 | 89.17 | 87.83 | 77.73 | 96.67 | 97.14 | 87.56 | 98.98 | 99.04 | 99.85 | 99.94 | 98.98 |
| VF-p + SULBA-p | 89.00 | 89.00 | 96.33 | 99.22 | 89.01 | 87.58 | 79.12 | **96.95** | **98.58** | 87.60 | 98.92 | 99.12 | 99.84 | **99.96** | 98.92 |
| RO-p + SULBA | 87.60 | 87.60 | 95.87 | 99.30 | 87.46 | 87.98 | 76.51 | 96.77 | 97.54 | 87.77 | **99.09** | 99.21 | **99.86** | 99.95 | **99.09** |
| RO-p + SULBA-p | 89.60 | 89.60 | 96.53 | 99.65 | 89.41 | **88.13** | **79.01** | 96.92 | 97.84 | **88.00** | 99.06 | 99.22 | **99.86** | 99.94 | 99.06 |

**Supplementary Table 7: Benchmark performance on 2D medical image classification**

| DA | ResNet-18 (Pretrained on ImageNet) (image size = 64 x 64) | | | | | SwinTransformer Tiny (Pretrained on ImageNet) (image size = 64 x 64) | | | | |
|---|---|---|---|---|---|---|---|---|---|---|
| | PathMNIST | | | | | PathMNIST | | | | |
| | A | Sen | Sp | AU | F1 | A | Sen | Sp | AU | F1 |
| BM | 95.36 | **94.09** | 99.43 | 99.61 | 95.36 | **96.89** | **95.98** | **99.61** | 99.77 | **96.87** |
| Cutout | **95.45** | 93.70 | **99.44** | 99.51 | **95.41** | 96.62 | 95.17 | 99.58 | 99.73 | 96.58 |
| Cutout-p | 95.14 | 93.66 | 99.41 | **99.70** | 95.16 | 96.56 | 95.32 | 99.57 | 99.85 | 96.53 |
| RE | 95.17 | 93.21 | 99.41 | 99.65 | 95.12 | 96.48 | 95.13 | 99.56 | **99.87** | 96.44 |
| RE-p | 95.35 | 94.06 | 99.42 | 99.28 | 95.30 | 96.60 | 95.16 | 99.58 | 99.73 | 96.55 |
| CutMix | 93.19 | 91.86 | 99.14 | 99.29 | 93.16 | 95.86 | 93.56 | 99.49 | 99.52 | 95.70 |
| CutMix-p | 93.79 | 92.06 | 99.23 | 99.10 | 93.75 | 96.27 | 95.47 | 99.54 | 99.79 | 96.26 |
| MixUp | 92.03 | 88.85 | 99.01 | 99.23 | 91.68 | 96.03 | 94.31 | 99.51 | 99.69 | 95.94 |
| MixUp-p | 94.26 | 92.19 | 99.29 | 99.50 | 94.18 | 96.20 | 95.13 | 99.53 | 99.56 | 96.17 |
| HF-p | 95.15 | 93.32 | 99.40 | 99.56 | 95.03 | 96.66 | 95.54 | 99.59 | 99.74 | 96.65 |
| VF-p | 95.06 | 93.59 | 99.39 | 99.65 | 95.06 | 96.55 | 95.33 | 99.58 | 99.67 | 96.51 |
| RO-p | 95.19 | 93.08 | 99.41 | 99.54 | 95.13 | 95.63 | 93.91 | 99.46 | 99.62 | 95.50 |
| SULBA | **95.39** | 93.99 | 99.43 | 99.42 | **95.39** | 96.64 | 95.24 | 99.59 | 99.70 | 96.64 |
| SULBA-p | 95.06 | 93.48 | 99.40 | 99.56 | 95.14 | 96.43 | 94.90 | 99.56 | 99.81 | 96.44 |
| | | | | | | | | | | |
| HF-p + SULBA | 95.10 | 93.61 | 99.40 | 99.60 | 95.09 | **96.88** | **95.83** | **99.62** | 99.84 | **96.86** |
| HF-p + SULBA-p | 94.50 | 93.32 | 99.33 | 99.60 | 94.56 | 96.14 | 95.05 | 99.53 | **99.86** | 96.16 |
| VF-p + SULBA | **95.42** | **94.34** | **99.44** | 99.58 | **95.45** | 96.57 | 95.50 | 99.58 | 99.39 | 96.54 |
| VF-p + SULBA-p | 94.86 | 93.18 | 99.37 | 99.60 | 94.87 | 96.49 | 95.19 | 99.57 | 99.81 | 96.50 |
| RO-p + SULBA | 95.25 | 93.84 | 99.42 | **99.67** | 95.24 | 96.63 | 95.30 | 99.59 | 99.79 | 96.61 |
| RO-p + SULBA-p | 95.13 | 93.79 | 99.40 | 99.52 | 95.15 | 96.14 | 94.08 | 99.52 | 99.74 | 96.04 |

| Supplementary Table 8: Per Dataset Cumulative Scores of Benchmark performance on 2D medical image classification | | | | | | | | | | | | |
|---|---|---|---|---|---|---|---|---|---|---|---|---|
| ResNet18 Pretrained on ImageNet | | | | | | | | | | | | |
| **DA** | **R** | **Breast MNIST** | **Tissue MNIST** | **Pneumonia MNIST** | **OrganA MNIST** | **OrganC MNIST** | **OrganS MNIST** | **Oct MNIST** | **Blood MNIST** | **Derma MNIST** | **Path MNIST** | **Total** |
| BM | 8 | 451.08 | 393.09 | 470.43 | 490.36 | 484.72 | 444.98 | 469.65 | 494.36 | 421.79 | **483.85** | 4604.31 |
| Cutout | 6 | 448.44 | 398.28 | 469.99 | 491.39 | 483.46 | 444.93 | 466.20 | 495.46 | 422.67 | 483.51 | 4604.33 |
| Cutout-p | 5 | 454.09 | 396.18 | 465.87 | 490.00 | 483.46 | 445.77 | 468.84 | 494.94 | 424.69 | 483.07 | 4606.91 |
| RE | 10 | 448.16 | 398.62 | 462.42 | 492.50 | **484.90** | 445.83 | 467.19 | 493.99 | 417.06 | 482.56 | 4593.23 |
| RE-p | 4 | 454.49 | 398.57 | 471.37 | 491.84 | 484.39 | 445.28 | 470.42 | 495.16 | 416.88 | 483.41 | 4611.81 |
| CutMix | 11 | 457.61 | 390.79 | 472.07 | **492.05** | 483.60 | 440.25 | 469.59 | 493.01 | 418.80 | 476.64 | 4594.41 |
| CutMix-p | 7 | 452.36 | 395.59 | 471.38 | 491.90 | 483.60 | 443.98 | 471.01 | 494.61 | 421.71 | 477.93 | 4604.07 |
| MixUp | 13 | 434.70 | 386.16 | 476.78 | 485.79 | 483.45 | 440.09 | 470.80 | 492.40 | 412.60 | 470.80 | 4553.57 |
| MixUp-p | 9 | 450.34 | 393.68 | 475.81 | 486.40 | 483.64 | 441.76 | 469.20 | 494.52 | 424.32 | 479.42 | 4599.09 |
| HF-p | 14 | 462.71 | 325.80 | 465.37 | 488.65 | 482.17 | 439.15 | 466.78 | 495.16 | 425.47 | 482.46 | 4533.72 |
| VF-p | 12 | 457.69 | 397.53 | 472.98 | 453.45 | 453.53 | 448.10 | 469.50 | 495.68 | 425.81 | 482.75 | 4557.02 |
| RO-p | 3 | 460.86 | 402.31 | 475.44 | 485.30 | 480.09 | 440.37 | 470.26 | **496.39** | **433.63** | 482.35 | 4627.00 |
| SULBA | 1 | **467.82** | **406.98** | 479.01 | 491.36 | 484.20 | 446.42 | 475.17 | 496.02 | 429.14 | 483.62 | 4659.74 |
| SULBA-p | 2 | 466.54 | 401.77 | **480.10** | 491.91 | 484.76 | **447.80** | **477.79** | 495.25 | 431.15 | 482.64 | 4659.71 |
| | | | | | | | | | | | | |
| HF-p + SULBA | 5 | 465.51 | 404.59 | **480.97** | 489.37 | 481.33 | 442.79 | 466.17 | 495.94 | 431.17 | 482.80 | 4640.64 |
| HF-p + SULBA-p | 6 | 462.94 | 403.96 | 474.53 | 489.69 | 481.13 | 443.53 | 470.74 | **496.93** | 433.76 | 481.31 | 4638.52 |
| VF-p + SULBA | 8 | 464.59 | 407.16 | 480.53 | 457.91 | 452.14 | 447.93 | 467.67 | 495.74 | 431.00 | **484.23** | 4588.90 |
| VF-p + SULBA-p | 7 | 467.98 | 406.10 | 475.24 | 458.49 | 456.40 | **447.97** | 472.12 | 495.96 | 429.89 | 481.88 | 4592.03 |
| RO-p + SULBA | 2 | **468.46** | **409.54** | 477.66 | 486.78 | 479.13 | 443.54 | 472.03 | 496.26 | **437.36** | 483.42 | 4654.18 |
| RO-p + SULBA-p | 4 | 468.00 | 408.62 | 472.30 | 486.86 | 480.55 | 438.67 | 469.24 | 496.40 | 433.76 | 482.99 | 4637.39 |
| HF-p + SULBA | 1 | 467.82 | 406.98 | 479.01 | 491.36 | 484.20 | 446.42 | 475.17 | 496.02 | 429.14 | 483.62 | 4659.74 |
| HF-p + SULBA-p | 3 | 466.54 | 401.77 | 480.10 | **491.91** | **484.76** | 447.80 | **477.79** | 495.25 | 431.15 | 482.64 | 4659.71 |

| Supplementary Table 9: Per Dataset Cumulative Scores of Benchmark performance on 2D medical image classification | | | | | | | | | | | |
|---|---|---|---|---|---|---|---|---|---|---|---|
| Swin Transformer (Tiny) Pretrained on ImageNet Cumulative Score) | | | | | | | | | | | |
| **DA** | **Breast MNIST** | **Tissue MNIST** | **Pneumonia MNIST** | **OrganA MNIST** | **OrganC MNIST** | **OrganS MNIST** | **Oct MNIST** | **Blood MNIST** | **Derma MNIST** | **Path MNIST** | **Total** |
| BM | 464.18 | 402.70 | 470.43 | 492.80 | 486.66 | 446.29 | 463.19 | 495.35 | 429.08 | 489.12 | 4639.80 |
| Cutout | 465.41 | 402.94 | 469.99 | 492.80 | 486.82 | 446.99 | 464.68 | 495.70 | 440.09 | 487.68 | 4653.10 |
| Cutout-p | 462.05 | 405.79 | 465.87 | 492.39 | **487.62** | 448.13 | 462.31 | 495.54 | 439.78 | 487.83 | 4647.31 |
| RE | 465.95 | 405.82 | 462.42 | 493.77 | 486.99 | 446.45 | 457.53 | 495.41 | 436.82 | 487.48 | 4638.64 |
| RE-p | 464.33 | 405.94 | 471.37 | **493.33** | 487.23 | 448.34 | 461.19 | 495.81 | 432.66 | 487.62 | 4647.82 |
| CutMix | 451.31 | 389.92 | 472.07 | 492.47 | 485.90 | 446.11 | 468.09 | 496.06 | 433.94 | 484.13 | 4620.00 |
| CutMix-p | 452.65 | 392.04 | 471.38 | 491.54 | 485.19 | 446.92 | 453.90 | 495.57 | 432.47 | 487.33 | 4608.99 |
| MixUp | 458.95 | 384.25 | 476.78 | 492.13 | 483.94 | 446.58 | 461.65 | 495.46 | 431.55 | 485.48 | 4616.77 |
| MixUp-p | 465.66 | 387.31 | 475.81 | 492.93 | 484.89 | 445.63 | 467.35 | 495.65 | 430.53 | 486.59 | 4632.35 |
| HF-p | **472.38** | 407.12 | 465.37 | 491.44 | 485.30 | 445.29 | 466.54 | 496.29 | 437.57 | **488.18** | 4655.48 |
| VF-p | 469.24 | 406.68 | 472.98 | 456.37 | 453.97 | 447.18 | 464.64 | 496.22 | 438.31 | 487.64 | 4593.23 |
| RO-p | 463.39 | 410.33 | 475.44 | 486.99 | 482.51 | 445.49 | 470.29 | 497.09 | **449.03** | 484.12 | 4664.68 |
| SULBA | 470.08 | **413.52** | 479.01 | 492.80 | 486.19 | **448.87** | **473.04** | **497.29** | 446.89 | 487.81 | **4695.50** |
| SULBA-p | 471.31 | 409.70 | **480.10** | 492.75 | 485.67 | 448.73 | 465.09 | 496.73 | 443.50 | 487.14 | 4680.72 |
| | | | | | | | | | | | |
| HF-p + SULBA | 470.49 | 411.38 | 478.77 | 490.80 | 482.95 | 446.99 | 467.06 | 497.09 | 445.15 | **489.03** | 4679.71 |
| HF-p + SULBA-p | **475.89** | 410.68 | 476.39 | **491.12** | **484.29** | **447.36** | **474.89** | 497.14 | 444.95 | 486.74 | **4689.45** |
| VF-p + SULBA | 473.84 | 412.31 | 481.20 | 455.34 | 454.27 | 446.53 | 463.59 | 496.79 | 446.93 | 487.58 | 4618.38 |
| VF-p + SULBA-p | 474.48 | 411.64 | 474.84 | 454.72 | 453.48 | 447.23 | 462.56 | 496.76 | 449.83 | 487.56 | 4613.10 |
| RO-p + SULBA | 469.55 | **414.83** | **482.07** | 487.93 | 481.65 | 445.23 | 457.83 | **497.20** | 446.57 | 487.92 | 4670.78 |
| RO-p + SULBA-p | 472.36 | 414.10 | 476.31 | 487.67 | 482.37 | 445.25 | 464.79 | 497.14 | **449.90** | 485.52 | 4675.41 |

| Supplementary Table 10: Per Architecture Cumulative Scores and Overall Ranking of Benchmark performance on 2D medical image classification | | | | |
|---|---|---|---|---|
| **DA** | **ResNet 18 Pretrained on ImageNet** | **Swin Transfromer (Tiny) Pretrained on ImageNet)** | **Total** | **Rank** |
| BM | 4604.31 | 4639.80 | 9244.11 | 7 |
| Cutout | 4604.33 | 4653.10 | 9257.43 | 5 |
| Cutout-p | 4606.91 | 4647.31 | 9254.22 | 6 |
| RE | 4593.23 | 4638.64 | 9231.87 | 8 |
| RE-p | 4611.81 | 4647.82 | 9259.63 | 4 |
| CutMix | 4594.41 | 4620.00 | 9214.41 | 10 |
| CutMix-p | 4604.07 | 4608.99 | 9213.06 | 11 |
| MixUp | 4553.57 | 4616.77 | 9170.34 | 13 |
| MixUp-p | 4599.09 | 4632.35 | 9231.44 | 9 |
| HF-p | 4533.72 | 4655.48 | 9189.20 | 12 |
| VF-p | 4557.02 | 4593.23 | 9150.25 | 14 |
| RO-p | 4627.00 | 4664.68 | 9291.68 | 3 |
| SULBA | **4659.74** | **4695.50** | **9355.24** | **1** |
| SULBA-p | 4659.71 | 4680.72 | 9340.43 | 2 |
| | | | | |
| HF-p + SULBA | 4640.64 | 4679.71 | 9320.35 | 3 |
| HF-p + SULBA-p | 4638.52 | **4689.45** | **9327.97** | **1** |
| VF-p + SULBA | 4588.90 | 4618.38 | 9207.28 | 5 |
| VF-p + SULBA-p | 4592.03 | 4613.10 | 9205.13 | 6 |
| RO-p + SULBA | **4654.18** | 4670.78 | 9324.96 | 2 |
| RO-p + SULBA-p | 4637.39 | 4675.41 | 9312.8 | 4 |

## Supplementary Tables 11 - 16: Benchmark performance on 3D medical image classification

(a) **DA =** Data Augmentation, **P** = Augmentation probability of 0.5, **BM** = Base Model, **ED** = Elastic Deformation, **SULBA** = Stepwise Upper and Lower Boundaries Augmentation, **A** = Accuracy, **Sen** = Sensitivity, **Sp** = Specificity, **AU** = AUROC, **F1** = F1 Score.

(b) Best performing method is represented in bold values

(c) Second best performing method is represented in underlined values

**Supplementary Table 11: Benchmark performance on 3D medical image classification**

**R(2+1)D-18 Pretrained on Kinetics-400 natural video dataset (image size = 64 x 64 x 64)**

| DA | OrganMNIST3D | | | | | SynapseMNIST3D | | | | | VesselMNIST3D | | | | |
|---|---|---|---|---|---|---|---|---|---|---|---|---|---|---|---|
| | A | Sen | Sp | AU | F1 | A | Sen | Sp | AU | F1 | A | Sen | Sp | AU | F1 |
| BM | 96.89 | 97.41 | 99.68 | 99.84 | 96.88 | 86.65 | 96.11 | 61.05 | 86.46 | 91.31 | 95.03 | 65.12 | 98.82 | 95.79 | 74.67 |
| Anisotropy | 95.90 | 96.59 | 99.58 | 99.77 | 95.90 | 88.35 | 96.89 | 65.26 | 86.96 | 92.39 | 95.81 | 69.77 | 99.12 | 90.96 | 78.95 |
| Anisotropy-p | 95.90 | 96.39 | 99.58 | 99.79 | 95.89 | 86.93 | 94.16 | 67.37 | 88.19 | 91.32 | 95.81 | 72.09 | 98.82 | 96.01 | 79.49 |
| Noise | 96.39 | 97.01 | 99.63 | 99.67 | 96.38 | 86.65 | 95.72 | 62.11 | 88.41 | 91.28 | 97.12 | 76.74 | **99.71** | 96.34 | 85.71 |
| Noise-p | 97.54 | 97.96 | 99.75 | 99.79 | 97.54 | 88.92 | **97.67** | 65.26 | **92.37** | 92.79 | 96.60 | 84.40 | 98.53 | 98.44 | 84.34 |
| BiasField | 96.56 | 97.15 | 99.65 | 99.77 | 96.57 | 87.22 | 97.28 | 60.00 | 89.44 | 91.74 | 95.55 | 81.40 | 97.35 | 98.35 | 80.46 |
| BiasField-p | 96.07 | 96.72 | 99.60 | 99.81 | 96.06 | 87.22 | 97.28 | 60.00 | 89.44 | 91.74 | 95.55 | 72.09 | 98.53 | 97.22 | 78.48 |
| Blur | 94.59 | 95.51 | 99.45 | 99.75 | 94.55 | 84.38 | 93.00 | 61.05 | 84.71 | 89.68 | 96.60 | 76.74 | 99.12 | 96.84 | 83.54 |
| Blur-p | 96.23 | 96.88 | 99.61 | 99.79 | 96.22 | 84.66 | 91.05 | 67.37 | 87.20 | 89.66 | 96.86 | 81.40 | 98.82 | 95.90 | 85.37 |
| ED | 96.72 | 97.27 | 99.67 | 99.79 | 96.70 | 83.81 | 96.50 | 49.47 | 84.62 | 89.69 | 96.34 | 76.74 | 98.82 | 97.28 | 82.50 |
| ED-p | 96.72 | 97.27 | 99.67 | 99.90 | 96.72 | 85.80 | 94.94 | 61.05 | 84.62 | 90.71 | 96.86 | 76.74 | 99.41 | 97.09 | 84.62 |
| Gamma | 97.05 | 97.53 | 99.70 | 99.83 | 97.04 | 85.51 | 94.94 | 60.00 | 87.51 | 90.54 | 95.55 | 76.74 | 97.94 | 96.90 | 79.52 |
| Gamma-p | 96.56 | 97.15 | 99.65 | 99.74 | 96.55 | 87.22 | 94.94 | 66.32 | 88.82 | 91.56 | 95.81 | 67.44 | 99.41 | 95.52 | 78.38 |
| Ghosting | 96.23 | 96.68 | 99.61 | 99.84 | 96.25 | 86.93 | 97.28 | 58.59 | 88.03 | 91.58 | 96.34 | 79.07 | 98.53 | 96.07 | 82.93 |
| Ghosting-p | 97.05 | 97.55 | 99.70 | 99.93 | 97.04 | 86.36 | 95.33 | 62.11 | 88.61 | 91.08 | 96.07 | 74.42 | 98.82 | 95.84 | 81.01 |
| Spike | 95.74 | 96.48 | 99.56 | 99.80 | 95.73 | 85.80 | 96.50 | 56.84 | 86.82 | 90.84 | 96.07 | 79.07 | 98.23 | 94.12 | 81.93 |
| Spike-p | 96.23 | 96.69 | 99.61 | 99.74 | 96.22 | 84.38 | 95.33 | 54.74 | 85.39 | 89.91 | 96.07 | 74.42 | 98.82 | 97.63 | 81.01 |
| Flip | 85.25 | 87.42 | 98.50 | 98.29 | 85.11 | 87.50 | 94.55 | 68.42 | 88.62 | 91.70 | 95.81 | 79.07 | 97.94 | 96.11 | 80.95 |
| Flip-p | 88.03 | 89.36 | 98.78 | 99.21 | 88.01 | 86.93 | 93.00 | **70.53** | 89.02 | 91.22 | 95.29 | 76.74 | 97.64 | 96.85 | 78.57 |
| SULBA | 97.54 | 97.98 | 99.75 | 99.94 | 97.54 | 88.64 | 95.72 | 69.47 | 89.56 | 92.48 | **98.17** | **86.05** | **99.71** | **98.84** | **91.36** |
| SULBA-p | **97.87** | **98.22** | **99.78** | **99.97** | **97.87** | **89.77** | 97.28 | 69.47 | 88.86 | **93.28** | 97.12 | **86.05** | 98.53 | 98.26 | 87.06 |

| **Supplementary Table 12: Benchmark performance on 3D medical image classification** | | | | | | | | | | | | | | | |
|---|---|---|---|---|---|---|---|---|---|---|---|---|---|---|---|
| **3D Swin Transformer Tiny Pretrained on Kinetics-400 natural video dataset (image size = 64 x 64 x 64)** | | | | | | | | | | | | | | | |
| **DA** | **OrganMNIST3D** | | | | | **SynapseMNIST3D** | | | | | **VesselMNIST3D** | | | | |
| | **A** | **Sen** | **Sp** | **AU** | **F1** | **A** | **Sen** | **Sp** | **AU** | **F1** | **A** | **Sen** | **Sp** | **AU** | **F1** |
| BM | 93.44 | 94.60 | 99.33 | 99.58 | 93.40 | 77.27 | 93.00 | 34.74 | 73.21 | 85.66 | 93.72 | 58.14 | 98.23 | 86.45 | 67.57 |
| Anisotropy | 94.26 | 95.27 | 99.41 | 99.59 | 94.27 | 80.40 | 91.05 | 51.58 | 78.02 | 87.15 | 95.29 | 62.79 | 99.41 | 92.28 | 75.00 |
| Anisotropy-p | 93.77 | 94.84 | 99.36 | 99.38 | 93.79 | 82.39 | 94.16 | 50.53 | 78.13 | 88.64 | 95.03 | 58.14 | **99.71** | 91.49 | 72.46 |
| Noise | 93.77 | 94.88 | 99.36 | 99.55 | 93.71 | 79.55 | **97.28** | 31.58 | 74.53 | 87.41 | 93.46 | 67.44 | 96.76 | 91.57 | 69.88 |
| Noise-p | 92.95 | 93.94 | 99.28 | 99.39 | 98.93 | 76.70 | 96.89 | 22.11 | 73.36 | 85.86 | 93.72 | 55.81 | 98.53 | 88.18 | 66.67 |
| BiasField | 93.44 | 94.37 | 99.33 | 99.37 | 93.43 | 80.97 | 87.94 | 62.11 | 82.57 | 87.09 | 93.98 | 48.84 | **99.71** | 87.41 | 64.62 |
| BiasField-p | 95.08 | 95.92 | 99.50 | 99.58 | 95.05 | 77.56 | 94.16 | 32.36 | 76.31 | 85.97 | 94.24 | 74.42 | 96.76 | 91.35 | 74.42 |
| Blur | 92.62 | 93.86 | 99.25 | 99.47 | 92.58 | 79.26 | 91.83 | 45.26 | 75.17 | 86.61 | 96.07 | 67.44 | **99.71** | 94.79 | 79.45 |
| Blur-p | 93.77 | 94.86 | 99.36 | 99.61 | 93.73 | 76.70 | 94.16 | 29.47 | 72.16 | 85.51 | 94.50 | 65.12 | 98.23 | 95.14 | 72.73 |
| ED | 93.93 | 94.40 | 99.38 | 99.57 | 93.92 | 79.55 | 94.55 | 38.95 | 79.47 | 87.10 | 95.03 | 60.47 | 99.41 | 94.85 | 73.24 |
| ED-p | 92.62 | 93.53 | 99.25 | 99.29 | 92.56 | 76.42 | 94.16 | 28.42 | 71.15 | 85.36 | 94.50 | 58.14 | 99.12 | 93.18 | 70.42 |
| Gamma | 92.95 | 94.19 | 99.28 | 99.42 | 92.97 | 76.99 | 90.27 | 41.05 | 71.96 | 85.14 | 93.72 | 58.14 | 98.23 | 86.45 | 67.57 |
| Gamma-p | 93.77 | 94.85 | 99.36 | 99.63 | 93.77 | 79.55 | 95.33 | 36.84 | 76.36 | 87.19 | 93.46 | 53.49 | 98.53 | 92.61 | 64.79 |
| Ghosting | 94.26 | 95.27 | 99.42 | 99.51 | 94.19 | 77.84 | 95.33 | 30.53 | 73.73 | 86.27 | 94.50 | 60.47 | 98.82 | 93.27 | 71.23 |
| Ghosting-p | 93.44 | 94.58 | 99.33 | 99.53 | 93.40 | 80.40 | 96.16 | 37.89 | 74.47 | 87.74 | 94.50 | 65.12 | 98.23 | 92.30 | 72.73 |
| Spike | 94.10 | 95.13 | 99.40 | 99.58 | 94.07 | 80.68 | 87.55 | 62.11 | 79.91 | 86.87 | 93.72 | 62.79 | 97.64 | 90.35 | 69.23 |
| Spike-p | 94.26 | 95.27 | 99.41 | 99.59 | 94.30 | 76.70 | 92.96 | 33.68 | 66.89 | 85.30 | 93.46 | 51.16 | 98.82 | 88.05 | 63.77 |
| Flip | 83.93 | 86.25 | 98.36 | 97.82 | 83.86 | 81.53 | 96.50 | 41.05 | 79.30 | 88.41 | 94.50 | 69.77 | 97.64 | 92.38 | 74.07 |
| Flip-p | 83.77 | 86.29 | 98.34 | 97.89 | 83.64 | 79.83 | 95.72 | 36.84 | 76.89 | 87.39 | 94.76 | 60.47 | 99.12 | 92.03 | 72.22 |
| SULBA | **95.74** | **96.47** | **99.56** | 99.72 | **95.73** | **83.24** | 90.66 | **63.16** | **84.01** | **88.76** | 96.07 | 74.42 | 98.82 | **96.82** | 81.01 |
| SULBA-p | 94.92 | 95.81 | 99.48 | **99.78** | 94.88 | 82.95 | 92.22 | 57.89 | 81.96 | **88.76** | **96.34** | **79.07** | 98.53 | 96.74 | **82.93** |

| **Supplementary Table 13: Benchmark performance on 3D medical image classification** | | | | | | | | | | | | | | | |
|---|---|---|---|---|---|---|---|---|---|---|---|---|---|---|---|
| **R(2+1)D-18 Pretrained on Kinetics-400 natural video dataset (image size = 64 x 64 x 64)** | | | | | | | | | | | | | | | |
| **DA** | **AdrenalMNIST3D** | | | | | **FractureMNIST3D** | | | | | **NoduleMNIST3D** | | | | |
| | **A** | **Sen** | **Sp** | **AU** | **F1** | **A** | **Sen** | **Sp** | **AU** | **F1** | **A** | **Sen** | **Sp** | **AU** | **F1** |
| BM | 84.23 | 52.17 | 93.89 | 85.67 | 60.50 | 52.50 | 46.96 | 74.17 | 61.94 | 51.10 | 88.39 | 65.62 | 94.31 | 94.14 | 70.00 |
| Anisotropy | 83.22 | 46.38 | 94.32 | 82.15 | 56.14 | 53.75 | 48.64 | 74.63 | 63.53 | 52.76 | 90.32 | 62.50 | 97.56 | 91.69 | 72.73 |
| Anisotropy-p | 83.89 | 56.52 | 92.14 | 87.26 | 61.90 | 53.75 | 48.63 | 75.16 | 64.85 | 52.91 | 88.71 | 65.62 | 94.72 | 89.04 | 70.59 |
| Noise | 86.24 | 44.93 | **98.69** | 82.57 | 60.19 | 54.58 | 52.40 | 75.79 | 68.13 | 53.92 | 88.39 | 53.12 | 97.56 | 86.94 | 65.38 |
| Noise-p | 85.23 | 50.72 | 95.63 | 84.84 | 61.40 | 54.17 | 48.90 | 75.14 | 64.18 | 53.33 | 89.68 | **76.56** | 93.09 | 89.88 | 75.38 |
| BiasField | 83.89 | 59.42 | 91.27 | 84.20 | 63.08 | 57.08 | 53.23 | 76.66 | 68.39 | 56.71 | 88.71 | 62.50 | 95.53 | 89.33 | 69.57 |
| BiasField-p | 85.23 | 44.93 | 97.38 | 83.57 | 58.49 | 55.42 | 51.18 | 76.07 | 63.88 | 55.01 | 88.06 | 50.00 | **97.97** | 86.95 | 63.33 |
| Blur | 85.23 | 60.87 | 92.58 | 87.74 | 65.62 | 56.67 | 53.75 | 76.78 | 67.14 | 55.83 | 89.35 | 73.44 | 93.50 | 89.31 | 74.02 |
| Blur-p | 84.23 | 47.83 | 95.20 | 87.40 | 58.41 | 56.25 | 52.07 | 76.75 | 68.07 | 55.57 | 88.39 | 70.31 | 93.09 | 89.45 | 71.43 |
| ED | 84.23 | 53.62 | 93.45 | 84.49 | 61.16 | 53.33 | 46.45 | 74.33 | 64.40 | 51.54 | 88.06 | 73.44 | 91.87 | 92.63 | 71.76 |
| ED-p | 83.89 | 49.28 | 94.32 | 84.04 | 58.62 | 54.58 | 49.72 | 75.72 | 66.53 | 53.17 | 88.71 | 65.62 | 94.72 | 88.81 | 70.59 |
| Gamma | 84.56 | 62.32 | 91.27 | 83.94 | 65.15 | 56.67 | 52.08 | 76.82 | 65.84 | 55.67 | 88.39 | 57.81 | 96.34 | 88.64 | 67.27 |
| Gamma-p | 84.23 | 44.93 | 96.07 | 84.25 | 56.88 | 53.75 | 49.65 | 75.46 | 61.97 | 53.69 | 88.71 | 70.31 | 93.50 | 91.01 | 72.00 |
| Ghosting | 84.56 | 52.17 | 94.32 | 84.98 | 61.02 | 54.58 | 50.89 | 75.84 | 64.26 | 54.26 | **90.97** | 64.06 | **97.97** | 88.47 | 74.55 |
| Ghosting-p | 85.57 | 49.28 | 96.51 | 83.32 | 61.26 | 53.33 | 48.47 | 74.64 | 63.00 | 52.46 | 89.35 | 59.38 | 97.15 | 91.44 | 69.72 |
| Spike | 85.91 | 65.22 | 92.14 | 88.37 | 68.18 | 57.08 | 51.50 | 76.90 | 62.70 | 55.90 | 89.68 | 75.00 | 93.50 | 88.80 | 75.00 |
| Spike-p | 85.23 | 47.83 | 96.51 | 85.81 | 60.00 | 56.66 | 52.95 | 77.05 | 68.97 | 56.46 | 88.39 | 59.38 | 95.93 | 88.57 | 67.86 |
| Flip | 85.91 | 44.93 | 98.25 | 85.62 | 59.62 | 56.25 | 52.17 | 76.71 | 69.05 | 55.96 | 89.35 | 62.50 | 96.34 | 92.90 | 70.80 |
| Flip-p | 85.23 | 65.22 | 91.27 | 87.96 | 67.16 | 57.08 | 48.82 | 76.02 | 62.62 | 53.95 | 90.32 | 67.19 | 96.34 | **94.25** | 74.14 |
| SULBA | **87.58** | **71.01** | 92.58 | 87.28 | **72.59** | 57.08 | **54.73** | 77.09 | **70.18** | 56.92 | **90.97** | 75.00 | 95.12 | 92.79 | **77.42** |

| | | | | | | | | | | | | | | | |
|---|---|---|---|---|---|---|---|---|---|---|---|---|---|---|---|
| SULBA-p | 86.91 | 62.32 | 94.32 | **90.65** | 68.80 | **59.18** | 54.42 | **77.76** | 67.63 | **58.38** | 90.00 | 75.00 | 93.90 | 91.57 | 75.59 |

**Supplementary Table 14: Benchmark performance on 3D medical image classification**

| 3D Swin Transformer Pretrained on Kinetics-400 natural video dataset (image size = 64 x 64 x 64) | | | | | | | | | | | | | | | |
|---|---|---|---|---|---|---|---|---|---|---|---|---|---|---|---|
| **DA** | **AdrenalMNIST3D** | | | | | **FractureMNIST3D** | | | | | **NoduleMNIST3D** | | | | |
| | **A** | **Sen** | **Sp** | **AU** | **F1** | **A** | **Sen** | **Sp** | **AU** | **F1** | **A** | **Sen** | **Sp** | **AU** | **F1** |
| BM | 82.89 | 56.52 | 90.83 | 80.52 | **60.47** | 57.50 | 48.84 | 75.98 | 64.47 | 54.03 | 88.06 | 60.94 | 95.12 | 84.06 | 67.83 |
| Anisotropy | 81.54 | 27.54 | **97.82** | 82.41 | 40.86 | 57.92 | 46.84 | 75.68 | 68.11 | 51.15 | 87.42 | 60.94 | 94.31 | 88.12 | 66.67 |
| Anisotropy-p | 82.21 | 53.62 | 90.83 | 82.07 | 58.27 | 57.50 | 47.02 | 75.73 | 66.82 | 51.36 | 89.03 | 62.50 | 95.93 | 83.94 | 70.18 |
| Noise | 81.54 | 40.58 | 93.89 | 75.53 | 50.45 | 52.08 | 44.73 | 73.36 | 64.45 | 49.62 | 89.03 | 67.19 | 94.72 | 85.50 | 71.67 |
| Noise-p | 81.88 | 34.78 | 96.07 | 76.54 | 47.06 | 56.25 | 45.86 | 74.93 | 66.61 | 50.16 | 87.10 | 53.12 | 95.93 | 85.86 | 62.96 |
| BiasField | 81.88 | 43.48 | 93.45 | 73.90 | 52.63 | 56.25 | 46.21 | 75.16 | 65.00 | 50.31 | 88.39 | 64.06 | 94.72 | 88.68 | 69.69 |
| BiasField-p | 81.21 | **60.87** | 87.34 | 79.06 | 60.00 | 54.58 | 44.38 | 74.04 | 65.56 | 48.57 | 86.45 | 43.75 | **97.56** | 85.16 | 57.14 |
| Blur | **83.89** | 50.72 | 93.89 | 81.73 | 59.32 | 56.25 | 45.91 | 75.02 | 64.15 | 50.13 | 87.74 | 57.81 | 95.53 | 89.85 | 66.07 |
| Blur-p | 81.88 | 39.13 | 94.76 | 74.85 | 50.00 | 52.92 | 47.28 | 74.90 | 67.73 | 51.70 | 88.39 | 54.69 | 97.15 | 84.97 | 66.04 |
| ED | 83.56 | 43.48 | 95.63 | 81.00 | 55.05 | 53.75 | 48.33 | 74.89 | 65.38 | 52.32 | 88.39 | 57.81 | 96.34 | 84.15 | 67.27 |
| ED-p | 82.55 | 42.03 | 94.76 | 77.52 | 52.73 | 54.58 | 44.23 | 73.95 | 63.85 | 48.18 | 86.45 | 68.75 | 91.06 | 88.29 | 67.69 |
| Gamma | 82.89 | 56.52 | 90.83 | 80.52 | **60.47** | 54.58 | 46.39 | 74.62 | 67.12 | 50.43 | 86.77 | 54.69 | 95.12 | 86.87 | 63.06 |
| Gamma-p | **83.89** | 42.03 | 96.51 | 76.30 | 54.72 | 58.75 | 51.42 | 77.09 | 66.11 | 56.30 | 86.77 | 48.44 | 96.75 | 80.41 | 60.79 |
| Ghosting | 80.87 | 28.99 | 96.51 | 79.92 | 41.24 | 49.58 | 40.73 | 71.47 | 61.84 | 44.45 | 87.74 | 51.56 | 97.15 | 80.84 | 63.46 |
| Ghosting-p | 82.21 | 43.48 | 93.45 | 81.37 | 52.63 | 54.58 | 44.03 | 73.76 | 64.39 | 47.89 | 87.10 | 57.81 | 94.72 | 88.91 | 64.91 |
| Spike | 81.88 | 43.48 | 93.45 | 81.37 | 52.63 | 53.75 | 43.24 | 73.25 | 63.46 | 46.83 | 88.06 | 56.25 | 96.34 | 86.53 | 66.06 |
| Spike-p | 82.21 | 39.13 | 95.20 | 80.44 | 50.47 | 56.67 | 51.84 | 76.54 | 67.27 | 55.92 | 89.03 | 65.62 | 95.12 | 87.18 | 71.19 |
| Flip | 81.21 | 33.33 | 95.63 | 73.77 | 45.10 | 53.75 | 44.49 | 73.87 | 66.27 | 48.07 | 87.74 | **75.00** | 91.06 | 88.23 | 71.64 |
| Flip-p | 81.54 | 40.58 | 93.89 | 82.30 | 50.45 | 55.42 | 46.83 | 75.11 | 65.14 | 50.55 | 89.35 | 62.50 | 96.34 | 88.95 | 70.80 |
| SULBA | 83.56 | 49.28 | 93.89 | **84.24** | 58.12 | **61.25** | 59.00 | **79.23** | **73.56** | **61.12** | 89.35 | 68.75 | 94.72 | **91.56** | 72.73 |
| SULBA-p | 82.89 | 49.28 | 93.01 | 76.66 | 57.14 | 60.00 | **59.91** | 79.01 | 73.23 | 60.07 | **90.00** | 68.75 | 95.55 | 85.62 | **73.95** |

**Supplementary Table 15: Per Dataset Cumulative Scores of Benchmark performance on 3D medical image classification**

| **DA** | **R(2+1)D-18 Pretrained on Kinetics-400 natural video dataset** | | | | | | Total |
|---|---|---|---|---|---|---|---|
| | Adrenal MNIST3D | Fracture MNIST3D | Nodule MNIST3D | Organ MNIST3D | Synapse MNIST3D | Vessel MNIST3D | |
| BM | 376.46 | 286.67 | 396.01 | 490.70 | 421.58 | 429.43 | 2400.85 |
| Anisotropy | 362.21 | 293.31 | 397.46 | 487.74 | 429.85 | 434.61 | 2405.18 |
| Anisotropy-p | 381.71 | 295.30 | 401.58 | 487.55 | 427.97 | 442.22 | 2436.33 |
| Noise | 372.62 | 304.82 | 408.11 | 489.08 | 424.17 | 455.62 | 2454.42 |
| Noise-p | 377.82 | 295.72 | 384.97 | 492.58 | 437.01 | 462.31 | 2450.41 |
| BiasField | 381.86 | 312.07 | 405.54 | 489.70 | 425.68 | 453.11 | 2467.96 |
| BiasField-p | 369.60 | 301.56 | 370.06 | 488.26 | 425.68 | 441.87 | 2397.03 |
| Blur | 392.04 | 310.17 | 397.00 | 483.85 | 412.82 | 452.84 | 2448.72 |
| Blur-p | 373.07 | 308.71 | 391.24 | 488.73 | 419.94 | 458.35 | 2440.04 |
| ED | 376.95 | 290.05 | 393.96 | 490.15 | 404.09 | 451.68 | 2406.88 |
| ED-p | 370.15 | 299.72 | 402.24 | 490.28 | 417.12 | 454.72 | 2434.23 |
| Gamma | 387.24 | 307.08 | 386.51 | 491.15 | 418.50 | 446.65 | 2437.13 |
| Gamma-p | 366.36 | 294.52 | 373.16 | 489.65 | 428.86 | 436.56 | 2389.11 |
| Ghosting | 377.05 | 299.83 | 380.75 | 488.61 | 422.41 | 452.94 | 2421.59 |
| Ghosting-p | 375.94 | 291.90 | 393.45 | 491.27 | 423.49 | 446.16 | 2422.21 |
| Spike | 399.82 | 304.08 | 393.24 | 487.31 | 416.80 | 449.42 | 2450.67 |
| Spike-p | 375.38 | 312.09 | 408.14 | 488.49 | 409.75 | 447.95 | 2441.80 |
| Flip | 374.33 | 310.14 | 413.67 | 454.57 | 430.79 | 449.88 | 2433.38 |
| Flip-p | 396.84 | 298.49 | 407.94 | 463.39 | 430.70 | 445.09 | 2442.45 |
| SULBA | **411.04** | 316.00 | **417.11** | 492.75 | 435.87 | **474.13** | **2546.90** |

| **DA** | **3D Swin Transformer Pretrained on Kinetics-400 natural video dataset** | | | | | | **Total** |
|---|---|---|---|---|---|---|---|
| | Adrenal MNIST3D | Fracture MNIST3D | Nodule MNIST3D | Organ MNIST3D | Synapse MNIST3D | Vessel MNIST3D | |
| BM | 371.23 | 300.82 | 396.01 | 480.35 | 363.88 | 404.11 | 2316.40 |
| Anisotropy | 330.17 | 299.70 | 397.46 | 482.80 | 388.20 | 424.77 | 2323.10 |
| Anisotropy-p | 367.00 | 298.43 | 401.58 | 481.14 | 393.85 | 416.83 | 2358.83 |
| Noise | 341.99 | 284.24 | 408.11 | 481.27 | 370.35 | 419.11 | 2305.07 |
| Noise-p | 336.33 | 293.81 | 384.97 | 484.49 | 354.92 | 402.91 | 2257.43 |
| BiasField | 345.34 | 292.93 | 405.54 | 479.94 | 400.68 | 394.56 | 2318.99 |
| BiasField-p | 368.48 | 287.13 | 370.06 | 485.13 | 366.36 | 431.19 | 2308.35 |
| Blur | 369.55 | 291.46 | 397.00 | 477.78 | 378.13 | 437.46 | 2351.38 |
| Blur-p | 340.62 | 294.53 | 391.24 | 481.33 | 358.00 | 425.72 | 2291.44 |
| ED | 358.72 | 294.67 | 393.96 | 481.20 | 379.62 | 423.00 | 2331.17 |
| ED-p | 349.59 | 284.79 | 402.24 | 477.25 | 355.51 | 415.36 | 2284.74 |
| Gamma | 371.23 | 293.14 | 386.51 | 478.81 | 365.41 | 404.11 | 2299.21 |
| Gamma-p | 353.45 | 309.67 | 373.16 | 481.38 | 375.27 | 402.88 | 2295.81 |
| Ghosting | 327.53 | 268.07 | 380.75 | 482.65 | 363.70 | 418.29 | 2240.99 |
| Ghosting-p | 353.14 | 284.65 | 393.45 | 480.28 | 376.66 | 422.88 | 2311.06 |
| Spike | 352.81 | 280.53 | 393.24 | 482.28 | 397.12 | 413.73 | 2319.71 |
| Spike-p | 347.45 | 308.24 | 408.14 | 482.83 | 355.53 | 395.26 | 2297.45 |
| Flip | 329.04 | 286.45 | 413.67 | 450.22 | 386.79 | 428.36 | 2294.53 |
| Flip-p | 348.76 | 293.05 | 407.94 | 449.93 | 376.67 | 418.60 | 2294.95 |
| SULBA | **369.09** | **334.16** | **417.11** | **487.22** | **409.83** | 447.14 | **2464.55** |

| | | | | | | | | | | | | | | |
|---|---|---|---|---|---|---|---|---|---|---|---|---|---|---|
| SULBA-p | 403.00 | **317.37** | 413.87 | **493.71** | **438.66** | 467.02 | 2533.63 | | 358.98 | 332.22 | 413.87 | 484.87 | 403.78 | **453.61** | 2447.33 |

**Supplementary Table 16: Per Architecture Cumulative Scores and Overall Rankingof Benchmark performance on 3D medical image classification**

| **DA** | **R(2+1)D-18 Pretrained on Kinetics-400 natural video dataset** | **3D Swin Transformer Pretrained on Kinetics-400 natural video dataset** | **Total** | **Rank** |
|---|---|---|---|---|
| BM | 2400.85 | 2316.40 | 4717.25 | 17 |
| Anisotropy | 2405.18 | 2323.10 | 4728.28 | 14 |
| Anisotropy-p | 2436.33 | 2358.83 | 4795.16 | 4 |
| Noise | 2454.42 | 2305.07 | 4759.49 | 7 |
| Noise-p | 2450.41 | 2257.43 | 4707.84 | 18 |
| BiasField | 2467.96 | 2318.99 | 4786.95 | 5 |
| BiasField-p | 2397.03 | 2308.35 | 4705.38 | 19 |
| Blur | 2448.72 | 2351.38 | 4800.10 | 3 |
| Blur-p | 2440.04 | 2291.44 | 4731.48 | 13 |
| ED | 2406.88 | 2331.17 | 4738.05 | 9 |
| ED-p | 2434.23 | 2284.74 | 4718.97 | 16 |
| Gamma | 2437.13 | 2299.21 | 4736.34 | 11 |
| Gamma-p | 2389.11 | 2295.81 | 4684.92 | 20 |
| Ghosting | 2421.59 | 2240.99 | 4662.58 | 21 |
| Ghosting-p | 2422.21 | 2311.06 | 4733.27 | 12 |
| Spike | 2450.67 | 2319.71 | 4770.38 | 6 |
| Spike-p | 2441.80 | 2297.45 | 4739.25 | 8 |
| Flip | 2433.38 | 2294.53 | 4727.91 | 15 |
| Flip-p | 2442.45 | 2294.95 | 4737.40 | 10 |
| SULBA | **2546.90** | **2464.55** | **5011.45** | **1** |
| SULBA-p | 2533.63 | 2447.33 | 4980.96 | 2 |

## Supplementary Tables 17-22: Benchmark performance on 2D medical image Segmentation

(a) **DA =** Data Augmentation, **P** = Augmentation probability of 0.5, **BM** = Base Model, **RE** = Random Erasing, **HF** = Random Horizontal Flip, **VF** = Random Vertical Flip, **RO** = Random Rotation (up to $360^{o}$), **SULBA** = Stepwise Upper and Lower Boundaries Augmentation, **IoU** = Intersection over Union, **F1** = F1 Score.

(b) Best performing method is represented in bold values

(c) Second best performing method is represented in underlined values

**Supplementary Table 17: Benchmark performance on 2D medical image Segmentation**

**U-Net with ImageNet-pretrained ResNet18 encoder**

**(image size = 128 x 128 x 128)**

| DA | AbdomenUSMSBench | | | | Bkai-Igh-MSBench | | | | Promise12MSBench | | | | MosMedPlusMSBench | | | |
|---|---|---|---|---|---|---|---|---|---|---|---|---|---|---|---|---|
| | IoU | Precision | Recall | F1 | IoU | Precision | Recall | F1 | IoU | Precision | Recall | F1 | IoU | Precision | Recall | F1 |
| BM | 65.46 | 85.34 | 73.09 | 68.65 | 72.94 | 90.73 | 78.93 | 75.51 | 82.69 | 89.33 | 91.94 | 89.84 | 65.29 | 81.11 | 76.70 | 75.86 |
| Cutout | 64.59 | 88.69 | 69.18 | 67.05 | 73.92 | 89.74 | 78.48 | 76.25 | 83.15 | 90.46 | 91.48 | 90.21 | 65.21 | 77.91 | 79.65 | 75.87 |
| Cutout-p | 64.76 | 86.80 | 71.96 | 67.58 | 74.32 | **92.12** | 78.06 | 76.40 | 83.03 | 89.46 | 91.91 | 90.05 | 65.16 | 79.69 | 76.90 | 75.82 |
| RE | 64.29 | **88.75** | 69.83 | 66.85 | 74.63 | 91.52 | 79.27 | 76.89 | 83.40 | 89.58 | 92.32 | 90.34 | 65.98 | 81.47 | 76.85 | 76.50 |
| RE-p | 63.93 | 87.52 | 69.33 | 66.42 | 73.06 | 88.78 | 78.23 | 75.83 | 83.54 | 89.93 | **92.48** | 90.47 | 65.32 | 80.08 | 77.36 | 75.97 |
| CutMix | 63.73 | 85.47 | 72.30 | 66.90 | 70.64 | 91.22 | 74.66 | 73.00 | 82.93 | 89.47 | 92.10 | 90.09 | 65.98 | 81.06 | 77.18 | 76.45 |
| CutMix-p | 64.15 | 87.40 | 71.34 | 67.14 | 71.34 | 89.27 | 74.77 | 73.94 | 82.51 | 89.42 | 91.73 | 89.67 | 65.87 | 81.35 | 77.38 | 76.45 |
| MixUp | 63.02 | 88.52 | 68.92 | 65.81 | 71.24 | 90.81 | 75.32 | 73.90 | 80.71 | 86.97 | 91.74 | 88.43 | 59.44 | **85.24** | 68.69 | 69.99 |
| MixUp-p | 64.65 | 86.61 | 71.31 | 67.43 | 74.69 | 91.45 | 78.30 | 76.94 | 81.33 | 89.52 | 89.96 | 88.87 | 62.56 | 78.51 | 75.36 | 73.33 |
| HF-p | 65.99 | 86.85 | 72.48 | 68.83 | 74.21 | 87.92 | 81.88 | 76.68 | 83.52 | 90.44 | 91.85 | 90.47 | 65.87 | 80.77 | 77.87 | 76.62 |
| VF-p | 65.27 | 87.98 | 71.10 | 68.02 | 74.37 | 90.72 | 79.08 | 76.44 | 83.11 | 90.36 | 91.40 | 90.18 | 65.07 | 81.30 | 76.38 | 75.65 |
| RO-p | **68.24** | 86.32 | **75.89** | **71.37** | **76.63** | 88.71 | **83.17** | **78.98** | 83.39 | 90.69 | 91.60 | 90.41 | 65.70 | 81.87 | 77.16 | 76.58 |
| SULBA | 68.04 | 86.27 | 75.75 | 71.24 | 75.42 | 91.41 | 79.91 | 77.65 | **84.16** | **90.86** | 92.22 | **90.92** | **67.28** | 79.56 | **80.28** | **77.81** |
| SULBA-p | 67.07 | 84.49 | **75.89** | 70.31 | 75.99 | 91.80 | 80.49 | 78.28 | 83.78 | 90.26 | 92.35 | 90.60 | 67.19 | 80.55 | 79.12 | 77.62 |
| | | | | | | | | | | | | | | | | |
| HF-p + SULBA | **69.25** | **90.33** | 74.43 | **72.31** | 75.28 | 90.72 | 79.60 | 77.24 | 83.66 | **91.21** | 91.43 | 90.61 | 66.24 | 82.28 | 77.76 | 76.94 |
| HF-p + SULBA-p | 68.79 | 87.48 | 75.65 | 72.03 | 75.49 | 89.61 | 80.92 | 77.67 | **84.05** | 90.29 | **92.80** | **90.88** | **67.09** | 82.94 | **78.77** | **77.72** |
| VF-p + SULBA | 67.29 | 85.26 | 75.88 | 70.58 | 75.47 | 90.05 | 80.17 | 77.55 | 83.77 | 90.91 | 91.76 | 90.67 | 66.62 | 83.37 | 76.58 | 77.33 |
| VF-p + SULBA-p | 66.21 | 86.01 | 73.74 | 69.23 | 75.73 | **91.35** | 79.75 | 77.97 | 83.26 | 90.96 | 91.16 | 90.28 | 67.06 | 81.93 | 78.26 | 77.63 |
| RO-p + SULBA | 68.63 | 86.22 | **76.64** | 71.95 | **76.70** | 88.90 | **82.19** | **78.84** | 83.53 | 90.44 | 92.03 | 90.42 | 65.07 | 82.05 | 77.59 | 75.84 |
| RO-p + SULBA-p | 69.11 | 87.93 | 75.28 | 72.25 | 76.34 | 90.75 | 81.13 | 78.71 | 83.51 | 90.88 | 91.66 | 90.46 | 64.94 | **85.16** | 73.78 | 75.62 |

**Supplementary Table 18: Benchmark performance on 2D medical image Segmentation**

**SegFormer model with ImageNet-pretrained MiT-B1**

**(image size = 128 x 128 x 128)**

| DA | AbdomenUSMSBench | | | | Bkai-Igh-MSBench | | | | Promise12MSBench | | | | MosMedPlusMSBench | | | |
|---|---|---|---|---|---|---|---|---|---|---|---|---|---|---|---|---|
| | IoU | Precision | Recall | F1 | IoU | Precision | Recall | F1 | IoU | Precision | Recall | F1 | IoU | Precision | Recall | F1 |
| BM | 65.34 | 86.41 | 72.25 | 68.39 | 71.68 | 89.69 | 76.92 | 74.29 | 81.89 | 88.84 | 91.56 | 89.29 | 58.44 | 77.79 | 73.00 | 69.98 |
| Cutout | 64.37 | 86.04 | 71.77 | 67.31 | 73.05 | 91.25 | 76.53 | 75.42 | 82.31 | **90.84** | 89.93 | 89.74 | 59.03 | 75.52 | 73.02 | 70.45 |
| Cutout-p | 63.54 | 85.01 | 71.54 | 66.42 | 73.26 | 90.32 | 76.76 | 75.74 | 82.21 | 88.70 | 91.81 | 89.57 | 57.79 | 79.57 | 68.34 | 69.27 |
| RE | 63.65 | 83.62 | 72.29 | 66.78 | 73.24 | 88.84 | 78.91 | 75.96 | 82.47 | 88.82 | 92.17 | 89.73 | **59.60** | 73.02 | **76.43** | **71.26** |
| RE-p | 63.75 | 82.85 | 73.48 | 66.88 | 73.89 | 89.92 | 78.34 | 76.82 | 81.91 | 88.27 | 92.05 | 89.34 | 58.90 | 76.74 | 71.54 | 70.32 |
| CutMix | 67.69 | 88.19 | 75.49 | 69.94 | 72.17 | 89.43 | 76.99 | 74.85 | 81.95 | 90.07 | 90.39 | 89.41 | 56.89 | 70.31 | 76.06 | 68.85 |
| CutMix-p | 67.49 | 86.96 | **75.60** | 69.81 | 71.50 | 90.25 | 75.38 | 74.51 | 81.65 | 88.73 | 91.38 | 89.23 | 57.92 | 78.72 | 69.70 | 69.25 |
| MixUp | 67.66 | 91.12 | 74.17 | 69.81 | 71.58 | 90.91 | 75.75 | 74.49 | 78.59 | 87.84 | 88.53 | 86.98 | 48.18 | **81.76** | 55.40 | 5979 |
| MixUp-p | **68.37** | **94.53** | 72.55 | 70.35 | 71.99 | 87.90 | 78.22 | 74.91 | 79.48 | 86.68 | 91.19 | 87.62 | 54.47 | 70.38 | 71.66 | 66.38 |
| HF-p | 65.73 | 85.81 | 73.64 | 68.85 | 74.78 | **91.51** | 78.62 | 77.32 | 82.47 | 87.93 | **93.14** | 89.71 | 58.67 | 71.77 | 77.24 | 70.66 |
| VF-p | 63.81 | 85.07 | 72.29 | 66.79 | 74.19 | 89.51 | 78.78 | 76.63 | 81.71 | 89.11 | 91.13 | 89.25 | 57.46 | 77.81 | 69.84 | 68.87 |
| RO-p | 67.04 | 86.72 | 74.15 | 69.87 | 74.64 | 86.74 | **80.45** | 76.96 | **83.37** | 89.36 | 92.86 | **90.42** | 57.04 | 76.62 | 70.33 | 68.32 |
| SULBA | 67.71 | 86.78 | 74.53 | **70.89** | **75.29** | 90.01 | 80.08 | **77.75** | 83.20 | 90.63 | 91.26 | 90.33 | 57.08 | 75.63 | 71.06 | 68.78 |
| SULBA-p | 67.42 | 86.51 | 74.36 | 70.60 | 74.64 | 90.36 | 78.39 | 77.16 | 82.85 | 90.00 | 91.49 | 90.10 | 59.33 | 73.25 | 76.22 | 71.03 |
| | | | | | | | | | | | | | | | | |
| HF-p + SULBA | **69.43** | **88.68** | **75.25** | **72.59** | 74.78 | 91.51 | 78.62 | 77.32 | 82.73 | 90.19 | 91.02 | 89.92 | 57.46 | **74.72** | 72.70 | 69.23 |
| HF-p + SULBA-p | 68.28 | 88.23 | 74.35 | 71.46 | 74.98 | **92.71** | 78.09 | 77.51 | 82.93 | 88.15 | **93.59** | 90.09 | 57.12 | 71.42 | 76.49 | 69.15 |
| VF-p + SULBA | 66.80 | 86.71 | 74.10 | 69.88 | 76.61 | 90.41 | **81.29** | 79.22 | 82.41 | 89.64 | 91.28 | 89.83 | 57.44 | 69.41 | **77.57** | **69.64** |
| VF-p + SULBA-p | 66.57 | 87.30 | 73.23 | 69.70 | **76.96** | 91.52 | 80.32 | **79.43** | 83.04 | 88.79 | 93.08 | 90.24 | **57.61** | 74.62 | 72.72 | 69.34 |
| RO-p + SULBA | 68.40 | 88.63 | 74.13 | 71.48 | 74.67 | 87.68 | **81.29** | 77.18 | 82.66 | **91.42** | 89.82 | 89.96 | 56.63 | 72.66 | 73.74 | 68.68 |
| RO-p + SULBA-p | 68.63 | 87.69 | 74.93 | 71.77 | 74.39 | 87.29 | 80.96 | 76.96 | **83.51** | 89.54 | 92.82 | **90.56** | 55.27 | 73.60 | 69.72 | 67.20 |

**Supplementary Table 19: Benchmark performance on 2D medical image Segmentation**

**U-Net with ImageNet-pretrained ResNet18 encoder**

| DA | FHPsAOPMSBench (image size = 128 x 128 x 128) | | | | CystoFluidMSBench (image size = 128 x 128 x 128) | | | | DeepbacsMSBench (image size = 512 x 512 x 512) | | | |
|---|---|---|---|---|---|---|---|---|---|---|---|---|
| | IoU | Precision | Recall | F1 | IoU | Precision | Recall | F1 | IoU | Precision | Recall | F1 |
| BM | 93.35 | 96.24 | 96.81 | 96.44 | 75.33 | 86.69 | 85.25 | 85.88 | 87.24 | 95.57 | 90.92 | 93.18 |
| Cutout | 93.57 | 96.50 | 96.74 | 96.55 | 75.32 | 86.12 | 85.84 | 85.89 | 86.92 | 94.76 | 91.30 | 93.00 |
| Cutout-p | 93.56 | 96.52 | 96.75 | 96.56 | 75.45 | 83.72 | **88.59** | 85.97 | 86.87 | 94.42 | 91.58 | 92.97 |
| RE | 93.58 | 96.60 | 96.68 | 96.56 | 75.41 | 86.07 | 86.00 | 85.95 | 87.54 | 94.16 | 92.57 | 93.35 |
| RE-p | 93.45 | 96.52 | 96.60 | 96.49 | 75.09 | 85.85 | 85.80 | 85.72 | 87.63 | **94.88** | 91.99 | 93.41 |
| CutMix | 92.53 | 96.17 | 95.94 | 95.93 | 76.41 | 85.54 | 87.85 | 86.58 | 87.82 | 94.85 | 92.23 | 93.51 |
| CutMix-p | 92.42 | 96.13 | 95.90 | 95.87 | 75.86 | 85.15 | 87.54 | 86.24 | 87.42 | 94.23 | 92.38 | 93.28 |
| MixUp | 90.43 | 95.19 | 94.64 | 94.61 | 72.50 | 84.86 | 83.46 | 83.79 | 88.04 | 94.93 | 92.40 | 93.64 |
| MixUp-p | 91.75 | 95.85 | 95.35 | 95.43 | 74.33 | 82.75 | 88.00 | 85.21 | 88.40 | 95.63 | 92.13 | 93.84 |
| HF-p | 93.80 | 96.52 | **96.99** | 96.70 | **77.68** | **87.42** | 87.84 | **87.81** | 88.00 | 94.13 | 93.13 | 93.61 |
| VF-p | 93.51 | 96.35 | 96.84 | 96.52 | 75.85 | 84.52 | 88.15 | 86.22 | 87.86 | 95.35 | 91.80 | 93.53 |
| RO-p | **94.00** | **96.81** | 96.93 | **96.82** | 76.49 | 86.22 | 87.24 | 86.64 | **88.88** | 94.69 | **93.55** | **94.11** |
| SULBA | 93.76 | 96.65 | 96.80 | 96.68 | 77.07 | 87.11 | 87.06 | 87.02 | 88.70 | 94.54 | 93.50 | 94.01 |
| SULBA-p | 93.73 | 96.68 | 96.76 | 96.66 | 76.76 | 85.82 | 88.04 | 86.82 | 88.68 | 95.37 | 92.69 | 94.00 |
| HF-p + SULBA | 93.88 | 96.91 | 96.68 | 96.75 | 76.62 | 88.17 | 87.23 | 86.62 | 88.52 | 95.29 | 92.58 | 93.90 |
| HF-p + SULBA-p | 93.81 | 96.75 | 96.77 | 96.70 | 76.65 | 87.91 | **87.99** | 86.86 | 88.51 | **95.79** | 92.10 | 93.90 |
| VF-p + SULBA | 93.86 | 96.58 | **97.01** | 96.74 | **76.84** | 88.03 | 85.94 | **86.87** | 88.44 | 94.97 | **92.80** | 93.86 |

| DA | IoU | Precision | Recall | F1 | IoU | Precision | Recall | F1 | IoU | Precision | Recall | F1 |
|---|---|---|---|---|---|---|---|---|---|---|---|---|
| VF-p + SULBA-p | 93.92 | 96.71 | 96.93 | 96.77 | 76.82 | 86.81 | 87.08 | 86.86 | 88.41 | 94.98 | 92.76 | 93.85 |
| RO-p + SULBA | **94.02** | 96.99 | 96.76 | **96.83** | 76.78 | 85.91 | **87.99** | 86.84 | **88.57** | 95.75 | 92.22 | **93.94** |
| RO-p + SULBA-p | 93.92 | **97.02** | 96.64 | 96.78 | 76.68 | **88.27** | 85.57 | 86.77 | 88.41 | 94.98 | 92.76 | 93.85 |

**Supplementary Table 20: Benchmark performance on 2D medical image Segmentation**

SegFormer model with ImageNet-pretrained MiT-B1

| DA | FHPsAOPMSBench (image size = 128 x 128 x 128) | | | | CystoFluidMSBench (image size = 128 x 128 x 128) | | | | DeepbacsMSBench (image size = 512 x 512 x 512) | | | |
|---|---|---|---|---|---|---|---|---|---|---|---|---|
| | IoU | Precision | Recall | F1 | IoU | Precision | Recall | F1 | IoU | Precision | Recall | F1 |
| BM | 92.90 | 95.90 | 96.58 | 96.15 | 58.96 | 72.89 | 75.48 | 74.00 | 62.91 | 77.46 | 77.34 | 77.06 |
| Cutout | 93.23 | 96.24 | 96.62 | 96.34 | 59.50 | 71.98 | 77.37 | 74.47 | 63.06 | 76.01 | 79.38 | 77.21 |
| Cutout-p | 93.10 | 96.28 | 96.43 | 96.28 | 59.29 | 74.00 | 74.71 | 74.31 | 63.27 | 76.67 | 79.79 | 77.36 |
| RE | 93.19 | 96.19 | 96.62 | 96.33 | 59.98 | 73.53 | 76.42 | 74.85 | 62.79 | 73.78 | 81.27 | 76.99 |
| RE-p | 93.35 | 96.30 | 96.71 | 96.44 | 59.54 | 72.78 | 76.42 | 74.85 | 63.19 | 77.20 | 78.15 | 77.27 |
| CutMix | 93.23 | 96.44 | 96.42 | 96.37 | 60.17 | 73.85 | 76.33 | 75.01 | 61.66 | 77.02 | 76.46 | 75.99 |
| CutMix-p | 93.20 | 96.37 | 96.47 | 96.36 | 59.48 | 70.46 | **79.18** | 74.49 | 61.58 | 70.38 | **83.51** | 75.89 |
| MixUp | 90.80 | 95.59 | 94.55 | 94.94 | 55.99 | **75.46** | 68.47 | 71.61 | 62.57 | 76.22 | 78.59 | 76.79 |
| MixUp-p | 91.77 | 95.79 | 95.46 | 95.50 | 58.01 | 72.49 | 74.34 | 73.29 | 61.95 | 77.26 | 76.09 | 76.24 |
| HF-p | 93.30 | 96.25 | 96.65 | 96.38 | **60.98** | 74.06 | 78.51 | 75.16 | 63.49 | 73.61 | 82.76 | 77.55 |
| VF-p | 93.41 | **96.48** | 96.61 | 96.48 | 58.73 | 74.26 | 73.66 | 73.85 | 63.62 | 76.30 | 79.97 | 77.60 |
| RO-p | **93.61** | 96.42 | **96.85** | **96.58** | 60.12 | 71.77 | 78.63 | 74.97 | **64.40** | **77.66** | 79.76 | **78.20** |
| SULBA | 93.45 | 96.31 | 96.79 | 96.49 | 60.85 | 74.29 | 77.00 | **75.55** | 63.27 | 77.02 | 78.35 | 77.34 |
| SULBA-p | 93.35 | 96.35 | 96.64 | 96.43 | 60.52 | 73.72 | 77.12 | 75.28 | 63.66 | 77.65 | 78.13 | 77.62 |
| | | | | | | | | | | | | |
| HF-p + SULBA | **93.69** | **96.58** | 96.78 | **96.64** | **61.68** | 71.45 | 77.06 | 75.16 | 64.34 | 76.64 | **80.37** | 78.17 |
| HF-p + SULBA-p | 93.68 | 96.35 | **97.00** | 96.61 | 61.39 | 73.70 | 78.22 | **75.70** | 62.84 | 80.59 | 74.63 | **79.96** |
| VF-p + SULBA | 93.53 | 96.53 | 96.66 | 96.54 | 60.00 | 71.77 | **78.49** | 74.88 | 63.89 | **81.72** | 74.95 | 77.74 |
| VF-p + SULBA-p | 93.62 | 96.44 | 96.84 | 96.59 | 59.76 | **74.94** | 74.67 | 74.69 | 63.01 | 76.77 | 78.56 | 77.17 |
| RO-p + SULBA | 93.57 | 96.57 | 96.68 | 96.58 | 60.26 | 74.18 | 76.25 | 75.09 | 64.03 | 79.79 | 76.95 | 77.87 |
| RO-p + SULBA-p | 93.57 | 96.37 | 96.82 | 96.55 | 60.36 | 73.00 | 77.71 | 75.14 | **64.36** | 77.23 | 79.93 | 78.16 |

**Supplementary Table 21: Per Dataset Cumulative Score of Benchmark performance on 2D medical image Segmentation**

| DA | U-Net with ImageNet-pretrained ResNet18 encoder | | | | | | | SegFormer model with ImageNet-pretrained MiT-B1 | | | | | | |
|---|---|---|---|---|---|---|---|---|---|---|---|---|---|---|
| | Abdomenus MSBench | BkaiIgh MSBench | Promise12 MSBench | MosMedPlus MSBench | FHPsAO MSBench | CystoFluid MSBench | Deepbacs MSBench | Abdomenus MSBench | BkaiIgh MSBench | Promise12 MSBench | MosMedPlus MSBench | FHPsAOP MSBench | CystoFluid MSBench | Deepbacs MSBench |
| BM | 292.54 | 318.11 | 353.80 | 298.96 | 382.84 | 333.15 | 366.91 | 292.39 | 312.58 | 351.58 | 279.21 | 381.53 | 281.33 | 348.16 |
| Cutout | 289.51 | 318.39 | 355.30 | 298.64 | 383.36 | 333.17 | 365.98 | 289.49 | 316.25 | 352.82 | 278.02 | 382.43 | 283.32 | 345.61 |
| Cutout-p | 291.10 | 320.90 | 354.45 | 297.57 | 383.39 | 333.73 | 365.84 | 286.51 | 316.08 | 352.29 | 274.97 | 382.09 | 282.31 | 344.80 |
| RE | 289.72 | 322.31 | 355.64 | 300.80 | 383.42 | 333.43 | 367.62 | 286.34 | 316.95 | 353.19 | 280.31 | 382.33 | 284.78 | 349.75 |
| RE-p | 287.20 | 315.90 | 356.42 | 298.73 | 383.06 | 332.46 | 367.91 | 286.96 | 318.97 | 351.57 | 277.50 | 382.8 | 283.59 | 346.72 |
| CutMix | 288.40 | 309.52 | 354.59 | 300.67 | 380.57 | 336.38 | 368.41 | 301.31 | 313.44 | 351.82 | 272.11 | 382.46 | 285.36 | 346.11 |
| CutMix-p | 290.03 | 309.32 | 353.33 | 301.05 | 380.32 | 334.79 | 367.31 | 299.86 | 311.64 | 350.99 | 275.59 | 382.40 | 283.61 | 345.89 |
| MixUp | 286.27 | 311.27 | 347.85 | 283.36 | 374.87 | 324.61 | 369.01 | 302.76 | 312.73 | 341.94 | 245.13 | 375.88 | 271.53 | 337.16 |
| MixUp-p | 290.00 | 321.38 | 349.68 | 289.76 | 378.38 | 330.29 | 370.00 | 305.80 | 313.02 | 344.97 | 262.89 | 378.52 | 278.13 | 335.76 |
| HF-p | 294.15 | 320.69 | 356.28 | 301.13 | 384.01 | 340.75 | 368.87 | 294.03 | 322.23 | 353.25 | 278.34 | 382.58 | 288.71 | 349.13 |
| VF-p | 292.37 | 320.61 | 355.05 | 298.40 | 383.22 | 334.74 | 368.54 | 287.96 | 319.11 | 351.20 | 273.98 | 382.98 | 280.50 | 350.17 |
| RO-p | 301.82 | 327.49 | 356.09 | 301.31 | 384.56 | 336.59 | 371.23 | 297.78 | 318.79 | 356.01 | 272.31 | 383.46 | 285.49 | 350.01 |
| SULBA | 301.30 | 324.39 | 358.16 | 304.93 | 383.89 | 338.26 | 370.75 | 299.91 | 323.13 | 355.42 | 272.55 | 383.04 | 287.69 | 352.53 |
| SULBA-p | 297.76 | 326.56 | 356.99 | 304.48 | 383.83 | 337.44 | 370.74 | 298.89 | 320.55 | 354.44 | 279.83 | 382.77 | 286.64 | 348.58 |
| | | | | | | | | | | | | | | |
| HF-p + SULBA | 306.32 | 322.84 | 356.91 | 303.22 | 384.22 | 338.64 | 370.29 | 305.95 | 322.23 | 353.86 | 274.11 | 383.69 | 285.35 | 348.13 |
| HF-p + SULBA-p | 303.95 | 323.69 | 358.02 | 306.52 | 384.03 | 339.41 | 370.30 | 302.32 | 323.29 | 354.76 | 274.18 | 383.64 | 289.01 | 349.64 |

| | | | | | | | | | | | | | | | |
|---|---|---|---|---|---|---|---|---|---|---|---|---|---|---|---|
| VF-p + SULBA | 299.01 | 323.24 | 357.11 | 303.9 | 384.19 | 337.68 | 370.07 | | 297.49 | 327.53 | 353.16 | 274.06 | 383.26 | 285.14 | 350.05 |
| VF-p + SULBA-p | 295.19 | 324.80 | 355.66 | 304.88 | 384.33 | 337.57 | 370.00 | | 296.80 | 328.23 | 355.15 | 274.29 | 383.49 | 284.06 | 348.88 |
| RO-p + SULBA | 303.44 | 326.63 | 356.42 | 300.55 | 384.60 | 337.52 | 370.48 | | 302.64 | 320.82 | 353.86 | 271.71 | 383.40 | 285.78 | 350.17 |
| RO-p + SULBA-p | 304.57 | 326.93 | 356.51 | 299.50 | 384.36 | 337.29 | 370.00 | | 303.02 | 319.60 | 356.43 | 274.11 | 384.36 | 286.21 | 351.50 |

**Supplementary Table 22: Per Architecture Cumulative Score and Overall Ranking of Benchmark performance on 2D medical image Segmentation**

| **DA** | **U-Net with ImageNet-pretrained ResNet18 encoder** | **SegFormer model with ImageNet-pretrained MiT-B1** | **Total** | **Rank** |
|---|---|---|---|---|
| BM | 2346.31 | 2246.78 | 4593.09 | 7 |
| Cutout | 2344.35 | 2247.94 | 4592.29 | 8 |
| Cutout-p | 2346.98 | 2239.05 | 4586.03 | 12 |
| RE | 2352.94 | 2253.65 | 4606.59 | 5 |
| RE-p | 2341.68 | 2248.11 | 4589.79 | 10 |
| CutMix | 2338.54 | 2252.61 | 4591.15 | 9 |
| CutMix-p | 2336.15 | 2249.98 | 4586.13 | 11 |
| MixUp | 2297.24 | 2187.13 | 4484.37 | 14 |
| MixUp-p | 2329.49 | 2219.09 | 4548.58 | 13 |
| HF-p | 2365.88 | 2268.27 | 4634.15 | 4 |
| VF-p | 2352.93 | 2245.90 | 4598.83 | 6 |
| RO-p | 2379.09 | 2263.85 | 4642.94 | 3 |
| SULBA | **2381.68** | **2274.27** | **4655.95** | **1** |
| SULBA-p | 2377.80 | 2271.70 | 4649.50 | 2 |
| | | | | |
| HF-p + SULBA | 2382.44 | 2273.32 | 4655.76 | 2 |
| HF-p + SULBA-p | **2385.92** | **2276.84** | **4662.76** | **1** |
| VF-p + SULBA | 2375.20 | 2270.69 | 4645.89 | **5** |
| VF-p + SULBA-p | 2372.43 | 2270.9 | 4643.33 | **6** |
| RO-p + SULBA | 2379.64 | 2268.38 | 4648.02 | **4** |
| RO-p + SULBA-p | 2379.16 | 2275.23 | 4654.39 | **3** |

**Supplementary Tables 23 - 26: Benchmark performance on 3D medical image Segmentation**

(a) MSD = Medical Segmentation Decathlon, **DA =** Data Augmentation, **P** = Augmentation probability of 0.5, **BM** = Base Model, **ED** = Elastic Deformation, **SULBA** = Stepwise Upper and Lower Boundaries Augmentation, **IoU** = Intersection over Union, **F1** = F1 Score.

(b) Best performing method is represented in bold values

(c) Second best performing method is represented in underlined values

| **Supplementary Table 23: Benchmark performance on 3D medical image Segmentation** | | | | | | | | | | | | |
|---|---|---|---|---|---|---|---|---|---|---|---|---|
| **3D U-Net** | | | | | | | | | | | | |
| **DA** | **IXITiny (image size = 32 x 32 x 32)** | | | | **Hippocampus MSD (image size = 64 x 64 x 64)** | | | | **Heart MSD (image size = 64 x 64 x 64)** | | | |
| | **IoU** | **Precision** | **Recall** | **F1** | **IoU** | **Precision** | **Recall** | **F1** | **IoU** | **Precision** | **Recall** | **F1** |
| BM | 95.23 | 97.57 | 97.54 | 97.56 | 72.96 | 84.11 | 84.60 | 84.35 | 51.85 | 70.61 | 64.94 | 67.66 |
| Anisotropy | 95.14 | 97.59 | 97.43 | 97.51 | 72.21 | 83.48 | 84.24 | 84.84 | 50.82 | 70.51 | 62.96 | 66.52 |
| Anisotropy-p | 95.24 | <u>97.76</u> | 97.37 | 97.56 | 71.66 | 83.56 | 83.39 | 83.46 | 50.41 | 66.90 | 66.07 | 66.47 |
| Noise | 95.31 | <u>97.76</u> | 97.44 | 97.60 | 72.32 | 84.35 | 83.53 | 83.92 | 50.58 | 66.99 | 66.44 | 66.68 |
| Noise-p | 95.34 | 97.59 | 97.64 | 97.62 | 72.68 | 84.41 | 83.99 | 84.16 | 49.47 | 65.94 | 65.59 | 65.74 |
| BiasField | 95.22 | 97.50 | 97.60 | 97.55 | 70.24 | 81.42 | 83.60 | 82.48 | 48.55 | 64.29 | 65.03 | 64.60 |
| BiasField-p | 95.27 | 97.64 | 97.51 | 97.58 | 72.23 | 84.05 | 83.68 | 83.85 | 50.26 | 64.82 | 67.39 | 66.03 |
| Blur | 95.09 | 97.56 | 97.41 | 97.48 | 72.23 | 84.44 | 83.30 | 83.86 | 51.35 | 72.19 | 62.49 | 66.98 |
| Blur-p | 95.24 | 97.73 | 97.39 | 97.56 | 71.60 | 83.19 | 83.69 | 83.43 | 48.40 | 68.20 | 61.35 | 64.59 |
| ED | 95.38 | 97.34 | <u>97.93</u> | 97.63 | 73.94 | 85.44 | 84.58 | 85.00 | 58.04 | **79.86** | 67.94 | 73.38 |
| ED-p | 95.39 | 97.70 | 97.58 | 97.64 | 73.17 | 84.74 | 84.29 | 84.49 | 54.06 | <u>77.88</u> | 63.42 | 69.91 |
| Gamma | 95.21 | 97.56 | 97.54 | 97.55 | 72.87 | 83.94 | 84.67 | 84.28 | 51.06 | 70.30 | 64.27 | 67.12 |
| Gamma-p | 95.21 | 97.44 | 97.65 | 97.55 | 72.82 | 84.06 | 84.45 | 84.25 | 50.83 | 69.24 | 63.82 | 66.41 |
| Ghosting | 95.30 | 97.69 | 97.40 | 97.59 | 72.74 | 84.55 | 83.90 | 84.20 | 52.31 | 74.02 | 62.68 | 67.87 |
| Ghosting-p | 95.14 | 97.40 | 97.62 | 97.51 | 71.93 | 83.79 | 83.54 | 83.65 | 49.43 | 61.63 | 69.85 | 65.47 |
| Spike | 95.28 | 97.47 | 97.69 | 97.58 | 73.15 | 84.04 | 84.95 | 84.48 | 48.62 | 70.10 | 59.57 | 64.41 |
| Spike-p | 95.31 | 97.54 | 97.66 | 97.60 | 73.24 | 83.90 | 85.20 | 84.53 | 50.83 | 67.52 | 66.44 | 66.96 |
| Flip | 95.29 | 97.69 | 97.49 | 97.59 | 73.39 | 84.07 | 85.24 | 84.64 | 50.68 | 72.22 | 62.30 | 66.81 |
| Flip-p | 95.30 | 97.54 | 97.64 | 97.59 | 73.32 | 84.38 | 84.83 | 84.59 | 47.90 | 66.98 | 60.90 | 63.76 |
| SULBA | **95.83** | **97.94** | 97.80 | **97.87** | **75.43** | <u>85.32</u> | **86.65** | **85.97** | **58.87** | 73.05 | **75.19** | **73.94** |
| SULBA-p | <u>95.79</u> | 97.68 | **98.02** | <u>97.85</u> | <u>75.09</u> | **85.89** | <u>85.62</u> | <u>85.75</u> | <u>58.72</u> | 73.91 | <u>73.88</u> | <u>73.85</u> |

**Supplementary Table 24: Benchmark performance on 3D medical image Segmentation**

| SwinUNETR | | | | | | | | | | | | |
|---|---|---|---|---|---|---|---|---|---|---|---|---|
| **DA** | **IXITiny (image size = 32 x 32 x 32)** | | | | **Hippocampus MSD (image size = 64 x 64 x 64)** | | | | **Heart MSD (image size = 64 x 64 x 64)** | | | |
| | **IoU** | **Precision** | **Recall** | **F1** | **IoU** | **Precision** | **Recall** | **F1** | **IoU** | **Precision** | **Recall** | **F1** |
| BM | 95.64 | 97.60 | 97.94 | 97.77 | 70.13 | 82.85 | 82.01 | 82.41 | 56.42 | 74.52 | 69.08 | 71.69 |
| Anisotropy | 95.39 | 97.65 | 97.63 | 97.64 | 68.83 | 82.74 | 80.34 | 81.51 | 52.23 | 67.27 | 68.53 | 67.89 |
| Anisotropy-p | 95.78 | 97.93 | 97.76 | 97.84 | 69.06 | 81.19 | 82.23 | 81.68 | 52.22 | 70.58 | 65.55 | 68.12 |
| Noise | 95.73 | 97.62 | 98.02 | 97.82 | 69.39 | 83.50 | 80.46 | 81.90 | 52.60 | 73.88 | 63.91 | 68.37 |
| Noise-p | 95.86 | 97.86 | 97.91 | 97.88 | 69.54 | 81.66 | 82.39 | 82.01 | 56.91 | 70.84 | 73.52 | 72.15 |
| BiasField | 95.67 | 97.87 | 97.70 | 97.79 | 69.15 | 81.20 | 82.28 | 81.72 | 53.11 | 69.15 | 68.98 | 69.02 |
| BiasField-p | 95.62 | 97.65 | 97.87 | 97.76 | 69.44 | 81.11 | 82.79 | 81.92 | 54.20 | 67.89 | 71.82 | 69.78 |
| Blur | 95.38 | 97.74 | 97.54 | 97.64 | 68.84 | 81.32 | 81.77 | 81.52 | 55.18 | 68.68 | 72.39 | 70.49 |
| Blur-p | 95.83 | 97.78 | 97.96 | 97.87 | 69.40 | 81.66 | 82.23 | 81.90 | 55.39 | 71.83 | 69.65 | 70.70 |
| ED | 95.77 | 97.63 | 98.06 | 97.84 | 70.72 | 83.73 | 81.96 | 82.81 | 61.72 | 77.71 | 74.82 | 76.23 |
| ED-p | 95.86 | 97.73 | 98.04 | 97.88 | 70.73 | 83.07 | 82.62 | 82.83 | 61.13 | 73.79 | 77.92 | 75.83 |
| Gamma | 95.76 | 97.54 | 98.13 | 97.83 | 70.14 | 81.48 | 83.42 | 82.42 | 53.80 | 69.47 | 69.46 | 69.29 |
| Gamma-p | 95.79 | 97.85 | 97.85 | 97.85 | 69.87 | 82.30 | 82.22 | 82.24 | 54.91 | 69.48 | 71.81 | 70.49 |
| Ghosting | 95.72 | 97.81 | 97.82 | 97.81 | 70.05 | 81.66 | 83.10 | 82.35 | 56.75 | 67.35 | 77.66 | 72.09 |
| Ghosting-p | 95.78 | 97.67 | 98.01 | 97.84 | 70.10 | 82.05 | 82.78 | 82.39 | 52.48 | 67.55 | 68.81 | 68.16 |
| Spike | 95.72 | 97.84 | 97.79 | 97.81 | 69.82 | 81.82 | 82.60 | 82.19 | 54.52 | 72.95 | 67.30 | 69.95 |
| Spike-p | 95.71 | 97.90 | 97.71 | 97.81 | 70.16 | 83.18 | 71.75 | 82.43 | 54.62 | 71.13 | 69.19 | 70.13 |
| Flip | 95.73 | 97.79 | 97.87 | 97.82 | 70.23 | 81.40 | 83.61 | 82.48 | 52.95 | 73.39 | 64.19 | 68.48 |
| Flip-p | 95.74 | 97.71 | 97.93 | 97.82 | 70.04 | 80.35 | 84.47 | 82.34 | 52.58 | 67.41 | 69.84 | 68.60 |
| SULBA | **96.30** | **98.05** | 98.19 | **98.12** | **73.79** | **85.05** | 84.76 | **84.89** | **65.74** | 76.76 | **78.78** | **79.26** |
| SULBA-p | 96.20 | 97.92 | **98.20** | 98.06 | 72.92 | 82.10 | **86.68** | 84.31 | 63.45 | **78.96** | 76.14 | 77.52 |

**Supplementary Table 25: Per Dataset Cumulative Score of Benchmark performance on 3D medical image Segmentation**

| **DA** | **3D U-Net** | | | | **SwinUNETR** | | |
|---|---|---|---|---|---|---|---|
| | **IXITiny** | **Hippocampus MSD** | **Heart MSD** | | **IXITiny** | **Hippocampus MSD** | **Heart MSD** |
| BM | 387.90 | 326.02 | 255.06 | | 388.95 | 317.40 | 271.71 |
| Anisotropy | 387.67 | 324.77 | 250.81 | | 388.31 | 313.42 | 255.92 |
| Anisotropy-p | 387.93 | 322.07 | 249.85 | | 389.31 | 314.16 | 256.47 |
| Noise | 388.11 | 324.12 | 250.69 | | 389.19 | 315.25 | 258.76 |
| Noise-p | 388.19 | 325.24 | 246.74 | | 389.51 | 315.60 | 273.42 |
| BiasField | 387.87 | 317.74 | 242.47 | | 389.03 | 314.35 | 260.26 |
| BiasField-p | 388.00 | 323.81 | 248.50 | | 388.90 | 315.26 | 263.69 |
| Blur | 387.54 | 323.83 | 253.01 | | 388.30 | 313.45 | 266.74 |
| Blur-p | 387.92 | 321.91 | 242.54 | | 389.44 | 315.19 | 267.57 |
| ED | 388.28 | 328.96 | 279.22 | | 389.30 | 319.22 | 290.48 |
| ED-p | 388.31 | 326.69 | 265.27 | | 389.51 | 319.25 | 288.67 |
| Gamma | 387.86 | 325.76 | 252.75 | | 389.26 | 317.46 | 262.02 |
| Gamma-p | 387.85 | 325.58 | 250.30 | | 389.34 | 316.63 | 266.69 |
| Ghosting | 387.98 | 325.39 | 256.88 | | 389.16 | 317.16 | 273.85 |
| Ghosting-p | 387.67 | 322.91 | 246.38 | | 389.30 | 317.32 | 257.00 |
| Spike | 388.02 | 326.62 | 242.70 | | 389.16 | 316.43 | 264.72 |
| Spike-p | 388.11 | 326.87 | 251.75 | | 389.13 | 307.52 | 265.07 |
| Flip | 388.06 | 327.34 | 252.01 | | 389.21 | 317.72 | 259.01 |
| Flip-p | 388.07 | 327.12 | 239.54 | | 389.20 | 317.20 | 258.43 |
| SULBA | **389.44** | **333.37** | **281.05** | | **390.66** | **328.49** | **300.54** |

| | | | | | | | |
|---|---|---|---|---|---|---|---|
| SULBA-p | 389.34 | 332.35 | 280.36 | | 390.38 | 326.01 | 296.07 |

| **Supplementary Table 26: Per Architecture Cumulative Score of Benchmark performance on 3D medical image Segmentation** | | | | |
|---|---|---|---|---|
| DA | **3D U-Net** | **SwinUNETR** | **Total** | **Rank** |
| BM | 968.98 | 978.06 | 1947.04 | 6 |
| Anisotropy | 963.25 | 957.65 | 1920.90 | 17 |
| Anisotropy-p | 959.85 | 959.94 | 1919.79 | 19 |
| Noise | 962.92 | 963.20 | 1926.12 | 15 |
| Noise-p | 960.17 | 978.53 | 1938.70 | 7 |
| BiasField | 948.08 | 963.64 | 1911.72 | 21 |
| BiasField-p | 960.31 | 967.85 | 1928.16 | 13 |
| Blur | 964.38 | 968.49 | 1932.87 | 11 |
| Blur-p | 952.37 | 972.20 | 1924.57 | 16 |
| ED | 996.46 | 999.00 | 1995.46 | 3 |
| ED-p | 980.27 | 997.43 | 1977.70 | 4 |
| Gamma | 966.37 | 968.74 | 1935.11 | 9 |
| Gamma-p | 963.73 | 972.66 | 1936.39 | 8 |
| Ghosting | 970.25 | 980.17 | 1950.42 | 5 |
| Ghosting-p | 956.96 | 963.62 | 1920.58 | 18 |
| Spike | 957.34 | 970.31 | 1927.65 | 14 |
| Spike-p | 966.73 | 961.72 | 1928.45 | 12 |
| Flip | 967.41 | 965.94 | 1933.35 | 10 |
| Flip-p | 954.73 | 964.83 | 1919.56 | 20 |
| SULBA | **1003.86** | **1019.69** | **2023.55** | **1** |
| SULBA-p | 1002.05 | 1012.46 | 2014.51 | 2 |

**Supplementary Table 27-29: Generalization performance of DA Techniques across diverse neural architectures using the PneumoniaNIST (Train set) and Chest X-ray Pneumonia Dataset (Test Set)**

(a) **DA =** Data Augmentation, **P** = Augmentation probability of 0.5, **BM** = Base Model, **RE** = Random Erasing, **HF** = Random Horizontal Flip, **VF** = Random Vertical Flip, **RO** = Random Rotation (up to 360°), **SULBA** = Stepwise Upper and Lower Boundaries Augmentation, **A** = Accuracy, **Sen** = Sensitivity, **Sp** = Specificity, **AU** = AUROC, **F1** = F1 Score.

(b) Best performing method is represented in bold values

(c) Second best performing method is represented in underlined values

**Supplementary Table 27: Generalization performance of DA Techniques across diverse neural architectures using the PneumoniaNIST (Train set) and Chest X-ray Pneumonia Dataset (Test Set)**

**With ImageNet-pretrained weights**

| DA | ResNet-18 | | | | | Swin Transformer Tiny | | | | | MobileNetV3 small | | | | | MobileVit_xxs Transformer Base | | | | |
|---|---|---|---|---|---|---|---|---|---|---|---|---|---|---|---|---|---|---|---|---|
| | A | Sen | Sp | AU | F1 | A | Sen | Sp | AU | F1 | A | Sen | Sp | AU | F1 | A | Sen | Sp | AU | F1 |
| BM | 92.79 | 93.33 | 91.88 | 97.80 | 94.18 | 94.39 | 96.41 | 91.03 | 97.59 | 95.55 | 86.70 | 89.74 | 81.62 | 92.36 | 89.40 | 92.95 | 97.18 | 85.90 | 97.18 | 94.51 |
| Cutout | 94.23 | **98.97** | 86.32 | 98.20 | 95.54 | 94.07 | 97.18 | 88.89 | 97.95 | 95.35 | 89.42 | 92.82 | 83.76 | 94.52 | 91.65 | 91.19 | 94.62 | 85.47 | 96.68 | 93.06 |
| Cutout-p | 93.59 | 96.41 | 88.89 | 97.58 | 94.95 | 92.79 | 96.15 | 87.18 | 97.92 | 94.34 | 88.94 | 90.51 | 86.32 | 94.36 | 91.10 | 90.71 | 93.08 | 86.75 | 96.07 | 92.60 |
| RE | 94.07 | 94.87 | **92.74** | 98.12 | 95.24 | 93.75 | 96.92 | 88.46 | 96.68 | 95.09 | 90.06 | 92.05 | **86.75** | 96.29 | 92.05 | 91.99 | 95.90 | 85.47 | 95.73 | 93.73 |
| RE-p | 93.75 | 95.38 | 91.03 | 98.01 | 95.02 | 93.91 | **97.95** | 87.18 | 97.87 | 95.26 | 90.22 | 92.56 | 86.32 | 95.28 | 92.21 | 93.27 | 97.69 | 85.90 | 97.45 | 94.78 |
| CutMix | 93.91 | 97.95 | 87.18 | 98.08 | 95.26 | 93.43 | 95.64 | 89.74 | 97.51 | 94.79 | 89.74 | 92.82 | 84.62 | 94.98 | 91.88 | **93.75** | 96.15 | 89.74 | **97.84** | **95.06** |
| CutMix-p | 94.87 | 97.18 | 91.03 | 97.73 | 95.95 | 94.07 | 97.69 | 88.03 | 97.35 | 95.37 | 90.87 | 95.13 | 83.76 | 95.76 | 92.87 | 91.83 | 96.92 | 83.33 | 97.28 | 93.68 |
| MixUp | 93.11 | 94.36 | 91.03 | 97.53 | 94.48 | 93.43 | 95.64 | 89.74 | 97.44 | 94.79 | 86.70 | 95.90 | 71.37 | 92.92 | 91.88 | 92.47 | 97.18 | 84.62 | 96.11 | 94.16 |
| MixUp-p | 94.87 | 96.67 | 91.88 | 98.08 | 95.93 | 93.59 | 97.18 | 87.61 | 97.37 | 94.99 | 89.78 | 92.82 | 84.62 | 96.23 | 91.88 | 90.71 | 91.03 | **90.17** | 96.11 | 92.45 |
| HF-p | 93.75 | 94.87 | 91.88 | 98.01 | 94.99 | 93.43 | 96.15 | 88.89 | 98.01 | 94.82 | 88.94 | 94.62 | 79.49 | 94.91 | 91.45 | 92.79 | 95.38 | 88.46 | 97.56 | 93.30 |
| VF-p | 94.23 | 98.72 | 86.75 | 97.80 | 95.53 | 93.75 | 96.15 | 89.74 | 97.27 | 95.06 | 86.70 | 95.64 | 71.79 | 91.85 | 89.99 | 91.19 | 96.67 | 82.05 | 96.69 | 93.20 |
| RO-p | 94.39 | 96.92 | 90.17 | 98.27 | 95.58 | 94.23 | 96.41 | 90.60 | 97.79 | 95.43 | 89.90 | **96.15** | 79.49 | 95.74 | 92.25 | 92.31 | 93.59 | **90.17** | 97.24 | 93.83 |
| SULBA | **95.19** | 98.46 | 89.74 | **98.67** | **96.24** | **95.19** | 97.18 | **91.88** | **98.40** | **96.19** | 90.87 | 95.13 | 83.76 | **96.40** | 92.87 | 93.48 | **98.72** | 84.62 | 97.41 | 94.94 |
| SULBA-p | 95.03 | 96.92 | 92.31 | 98.23 | 96.18 | 94.87 | **97.95** | 89.74 | 98.28 | 95.98 | **91.03** | 94.87 | 84.62 | 95.27 | **92.96** | 92.31 | 94.36 | 88.89 | 96.72 | 93.88 |

**Supplementary Table 28: Generalization performance of DA Techniques across diverse neural architectures using the PneumoniaNIST (Train set) and Chest X-ray Pneumonia Dataset (Test Set)**

**With Randomly Initialized Weights (standard Networks)**

| DA | ResNet-18 | | | | | Swin Transformer Tiny | | | | | MobileNetV3 small | | | | | MobileVit_xxs Transformer Base | | | | |
|---|---|---|---|---|---|---|---|---|---|---|---|---|---|---|---|---|---|---|---|---|
| | A | Sen | Sp | AU | F1 | A | Sen | Sp | AU | F1 | A | Sen | Sp | AU | F1 | A | Sen | Sp | AU | F1 |
| BM | 87.50 | 89.74 | 83.76 | 94.60 | 89.97 | 81.41 | 84.62 | 76.07 | 89.84 | 85.05 | 79.33 | 92.05 | 58.12 | 85.65 | 84.77 | 80.61 | 84.87 | 73.50 | 89.08 | 84.55 |
| Cutout | 84.94 | 86.92 | 81.62 | 93.68 | 87.32 | 81.89 | 84.62 | 77.35 | 88.96 | 85.38 | 83.01 | 87.18 | 76.07 | 88.99 | 86.51 | 82.69 | 83.08 | 82.05 | 90.96 | 85.71 |
| Cutout-p | 87.02 | 89.23 | 83.33 | 95.23 | 89.54 | 83.81 | 90.77 | 72.22 | 92.70 | 87.52 | 84.94 | 89.23 | 77.78 | 89.82 | 88.10 | 77.56 | 78.97 | 75.21 | 86.07 | 81.48 |
| RE | 84.13 | 77.95 | **94.44** | 95.37 | 86.00 | 79.81 | 74.36 | 88.89 | 91.68 | 82.15 | 87.50 | **95.02** | 79.91 | 92.02 | 90.20 | 82.21 | 83.33 | 80.34 | 87.43 | 85.41 |
| RE-p | 88.94 | 92.82 | 82.48 | 95.48 | 91.30 | 83.49 | 82.82 | 84.62 | 91.06 | 86.25 | 85.45 | 91.54 | 75.21 | 90.29 | 88.70 | 81.25 | 88.21 | 69.66 | 88.92 | 85.47 |
| CutMix | 90.22 | 91.03 | 88.89 | 95.72 | 92.09 | 84.46 | 80.00 | 91.88 | 93.45 | 86.55 | 85.42 | 87.44 | 82.05 | 92.60 | 88.23 | 85.26 | 88.72 | 79.49 | 92.91 | 88.27 |
| CutMix-p | 87.34 | 83.85 | 93.16 | 95.36 | 89.22 | 83.01 | 76.92 | **93.16** | 93.71 | 84.99 | 84.94 | 94.36 | 69.23 | 91.47 | 88.67 | 79.97 | 78.46 | 82.48 | 88.75 | 83.04 |
| MixUp | 89.26 | 88.21 | 91.03 | 95.97 | 91.13 | 78.85 | 74.36 | 86.32 | 90.77 | 81.46 | 80.45 | 88.75 | 66.67 | 84.21 | 85.01 | 82.05 | 81.28 | 83.33 | 90.39 | 84.99 |
| MixUp-p | 86.54 | 90.77 | 79.49 | 94.49 | 89.39 | 82.53 | 85.64 | 77.35 | 90.42 | 85.97 | 83.65 | 91.03 | 71.37 | 88.73 | 87.44 | 70.19 | 54.36 | **96.58** | 85.67 | 69.51 |
| HF-p | 88.14 | 91.54 | 82.48 | 94.49 | 90.61 | 82.21 | 81.03 | 84.19 | 90.40 | 85.06 | 85.10 | 93.08 | 71.79 | 91.01 | 88.64 | 81.89 | 87.18 | 73.08 | 89.00 | 85.75 |
| VF-p | 83.97 | 80.77 | 89.32 | 94.13 | 86.30 | 81.09 | 77.95 | 86.32 | 90.48 | 83.75 | 80.93 | 84.36 | 75.21 | 85.56 | 84.68 | 82.85 | 83.59 | 81.62 | 89.45 | 85.90 |
| RO-p | 85.26 | 82.31 | 90.17 | 94.36 | 87.47 | 86.06 | 82.82 | 91.45 | 94.15 | 88.13 | 86.06 | 90.26 | 79.06 | 92.61 | 89.00 | 84.29 | 83.85 | 85.04 | 92.28 | 86.97 |
| SULBA | **91.51** | **98.21** | 80.34 | **97.76** | **93.53** | 87.66 | 85.64 | 91.03 | 96.15 | 89.66 | **89.10** | 91.03 | **85.90** | **94.92** | **91.26** | **89.10** | **98.36** | 80.34 | **95.46** | **91.54** |

| SULBA-p | 91.35 | 94.36 | 86.32 | 96.69 | 93.16 | **90.87** | **91.79** | 89.32 | **96.62** | **92.63** | 87.82 | 91.97 | 81.20 | 93.65 | 90.40 | 88.46 | 93.33 | 80.34 | 92.44 | 91.00 |
|---|---|---|---|---|---|---|---|---|---|---|---|---|---|---|---|---|---|---|---|---|

**Supplementary Table 29: Per Architecture Cumulative Score of the Generalization performance of DA Techniques across diverse neural architectures using the PneumoniaNIST (Train set) and Chest X-ray Pneumonia Dataset (Test Set)**

| DA | With ImageNet-pretrained Networks | Randomly Initialized Networks | Total | Rank |
|---|---|---|---|---|
| BM | 1852.49 | 1675.09 | 3527.58 | 13 |
| Cutout | 1859.89 | 1698.93 | 3558.82 | 9 |
| Cutout-p | 1850.24 | 1700.53 | 3550.77 | 10 |
| RE | 1865.96 | 1718.15 | 3584.11 | 7 |
| RE-p | 1871.04 | 1723.96 | 3595.00 | 5 |
| CutMix | 1870.07 | 1764.68 | 3634.75 | 3 |
| CutMix-p | 1870.70 | 1722.09 | 3592.79 | 6 |
| MixUp | 1844.86 | 1694.49 | 3539.35 | 11 |
| MixUp-p | 1863.97 | 1661.12 | 3525.09 | 14 |
| HF-p | 1861.70 | 1716.67 | 3578.37 | 8 |
| VF-p | 1840.77 | 1688.23 | 3529.00 | 12 |
| RO-p | 1870.46 | 1751.60 | 3622.06 | 4 |
| SULBA | **1885.34** | **1818.50** | **3703.84** | **1** |
| SULBA-p | 1880.40 | 1813.72 | 3694.12 | 2 |

**Supplementary Tables 30 and 31: Datasets and Training Details**

**NTr** = Number of Train Set Samples, **Nte** = Number of Test Set Samples, **IS** = Image Size, **E** = Number of Train Epoch, **LR** = Learning rate, **BS** = Batch Size, **MSD** = Medical Segmentation Decathlon, **NC** = Number of Classes

| **Supplementary Table 30: Datasets and Training Details** | | | | | | | |
|---|---|---|---|---|---|---|---|
| **Dataset** | **NTr** | **NTe** | **IS** | **E** | **LR** | **BS** | **NC** |
| 2D Classification | | | | | | | |
| BloodMNIST | 11,959 | 3,421 | 64 × 64 | 100 | $1 \times 10^{-4}$ | 32 | 8 |
| BreastMNIST | 546 | 456 | 64 × 64 | 100 | $1 \times 10^{-4}$ | 32 | 2 |
| DermaMNIST | 7,007 | 2,005 | 64 × 64 | 100 | $1 \times 10^{-4}$ | 32 | 7 |
| OctMNIST | 97,477 | 1,000 | 64 × 64 | 100 | $1 \times 10^{-4}$ | 32 | 4 |
| OrganAMNIST | 34,561 | 17,778 | 64 × 64 | 100 | $1 \times 10^{-4}$ | 32 | 11 |
| OrganCMNIST | 12,975 | 8,216 | 64 × 64 | 100 | $1 \times 10^{-4}$ | 32 | 11 |
| OrganSMNIST | 13,932 | 8,827 | 64 × 64 | 100 | $1 \times 10^{-4}$ | 32 | 11 |
| PathMNIST | 89,996 | 7,180 | 64 × 64 | 100 | $1 \times 10^{-4}$ | 32 | 9 |
| PneumoniaMNIST | 4,708 | 624 | 64 × 64 | 100 | $1 \times 10^{-4}$ | 32 | 2 |
| TissueMNIST | 165,466 | 47,280 | 64 × 64 | 100 | $1 \times 10^{-4}$ | 32 | 8 |
| 3D Classification | | | | | | | |
| AdrenalMNIST3D | 1188 | 298 | 64 × 64 × 64 | 100 | $1 \times 10^{-4}$ | 32 | 2 |
| FractureMNIST3D | 1027 | 240 | 64 × 64 × 64 | 100 | $1 \times 10^{-4}$ | 32 | 3 |
| NoduleMNIST3D | 1158 | 310 | 64 × 64 × 64 | 100 | $1 \times 10^{-4}$ | 32 | 2 |
| OrganMNIST3D | 971 | 610 | 64 × 64 × 64 | 100 | $1 \times 10^{-4}$ | 32 | 11 |
| SynapseMNIST3D | 1230 | 352 | 64 × 64 × 64 | 100 | $1 \times 10^{-4}$ | 32 | 2 |
| VesselMNIST3D | 1335 | 382 | 64 × 64 × 64 | 100 | $1 \times 10^{-4}$ | 32 | 2 |
| 2D Segmentation | | | | | | | |
| AbdomenUSMSBench | 569 | 293 | 128 × 128 | 100 | $1 \times 10^{-3}$ | 16 | 9 |
| Bkai-Igh-MSBench | 700 | 200 | 128 × 128 | 100 | $1 \times 10^{-3}$ | 16 | 3 |
| CystoFluidMSBench | 703 | 202 | 128 × 128 | 100 | $1 \times 10^{-3}$ | 16 | 2 |
| DeepbacsMSBench | 17 | 15 | 512 × 512 | 100 | $1 \times 10^{-3}$ | 2 | 2 |
| FHPsAOPMSBench | 280 | 80 | 128 × 128 | 100 | $1 \times 10^{-3}$ | 16 | 3 |
| MosMedPlusMSBench | 1,910 | 547 | 128 × 128 | 100 | $1 \times 10^{-3}$ | 16 | 2 |
| Promise12MSBench | 1,031 | 295 | 128 × 128 | 100 | $1 \times 10^{-3}$ | 16 | 2 |
| 3D Segmentation | | | | | | | |
| IXITiny | 396 | 170 | 32 × 32 × 32 | 100 | $1 \times 10^{-4}$ | 16 | 2 |
| MSD (Heart) | 14 | 6 | 32 × 32 × 32 | 100 | $1 \times 10^{-4}$ | 4 | 2 |
| MSD (Hippocampus) | 182 | 78 | 32 × 32 × 32 | 100 | $1 \times 10^{-3}$ | 16 | 3 |
| Cross Dataset Generalization | | | | | | | |
| PneumoniaMNIST | 4,708 | - | 64 × 64 | 100 | $1 \times 10^{-4}$ | 32 | 2 |
| chest X-ray pneumonia | - | 624 | 64 × 64 | - | $1 \times 10^{-4}$ | 32 | 2 |

| Supplementary Table 31: Datasets Sources | |
|---|---|
| Dataset | Source |
| BloodMNIST | Yang J, Shi R, Wei D, Liu Z, Zhao L, Ke B, Pfister H, Ni B. Medmnist v2-a large-scale lightweight benchmark for 2d and 3d biomedical image classification. Scientific Data. 2023 Jan 19;10(1):41. |
| BreastMNIST | |
| DermaMNIST | |
| OctMNIST | |
| OrganAMNIST | |
| OrganCMNIST | |
| OrganSMNIST | |
| PathMNIST | |
| PneumoniaMNIST | |
| TissueMNIST | |
| AdrenalMNIST3D | |
| FractureMNIST3D | |
| NoduleMNIST3D | |
| OrganMNIST3D | |
| SynapseMNIST3D | |
| VesselMNIST3D | |
| | |
| AbdomenUSMSBench | Kuş Z, Aydin M. MedSegBench: A comprehensive benchmark for medical image segmentation in diverse data modalities. Scientific Data. 2024 Nov 25;11(1):1283. |
| Bkai-Igh-MSBench | |
| CystoFluidMSBench | |
| DeepbacsMSBench | |
| FHPsAOPMSBench | |
| MosMedPlusMSBench | |
| Promise12MSBench | |
| | |
| MSD (Heart) | Antonelli M, Reinke A, Bakas S, Farahani K, Kopp-Schneider A, Landman BA, Litjens G, Menze B, Ronneberger O, Summers RM, Van Ginneken B. The medical segmentation decathlon. Nature communications. 2022 Jul 15;13(1):4128. |
| MSD (Hippocampus) | |
| | |
| IXITiny | Pérez-García F, Sparks R, Ourselin S. TorchIO: a Python library for efficient loading, preprocessing, augmentation and patch-based sampling of medical images in deep learning. Computer methods and programs in biomedicine. 2021 Sep 1;208:106236. |
| | |
| chest X-ray pneumonia | Kermany DS, Goldbaum M, Cai W, Valentim CC, Liang H, Baxter SL, McKeown A, Yang G, Wu X, Yan F, Dong J. Identifying medical diagnoses and treatable diseases by image-based deep learning. cell. 2018 Feb 22;172(5):1122-31. |
